# Data for validation of osteometric methods in forensic anthropology

**DOI:** 10.1016/j.dib.2018.04.148

**Published:** 2018-05-07

**Authors:** Natalie R. Langley, Lee Meadows Jantz, Shauna McNulty, Heli Maijanen, Stephen D. Ousley, Richard L. Jantz

**Affiliations:** a Department of Anatomy, Mayo Clinic College of Medicine and Science, Mayo Clinic School of Medicine Arizona Campus, 13400 E. Shea Blvd., Scottsdale, AZ 85259, USA; b Department of Anthropology, University of Tennessee, 502 Strong Hall, 1621 Cumberland Ave., Knoxville, TN 37996, USA; c Science Department, Umpqua Community College, 1140 Umpqua College Road, Roseburg, OR 97470, USA; d University of Oulu, University of Oulu, Archaeology, P.O. Box 1000, 90014, Finland; e Department of Mathematics and Information Technology, Mercyhurst University, 501 E. 38th Street, Erie, PA 16546, USA

## Abstract

Many techniques in forensic anthropology employ osteometric data, although little work has been done to investigate the intrinsic error in these measurements. These data were collected to quantify the reliability of osteometric data used in forensic anthropology research and case analyses. Osteometric data (n = 99 measurements) were collected on a random sample of William M. Bass Donated Collection skeletons (n = 50 skeletons). Four observers measured the left elements of 50 skeletons. After the complete dataset of 99 measurements was collected on each of the 50 skeletons, each observer repeated the process for a total of four rounds. The raw data is available on Mendeley Data (***DCP Osteometric Data, Version 1.** DOI: 10.17632/6xwhzs2w38.1*). An example of the data analyses performed to evaluate and quantify observer error is provided for the variable GOL (maximum cranial length); these analyses were performed on each of the 99 measurements. Two-way mixed ANOVAs and repeated measures ANOVAs with pairwise comparisons were run to examine intraobserver and interobserver error, and relative and absolute technical error of measurement (TEM) was calculated to quantify the observer variation. This data analysis supported the dissemination of a free laboratory manual of revised osteometric definitions (*Data Collection Procedures 2.0*[1], pdf available at https://fac.utk.edu/wp-content/uploads/2016/03/DCP20_webversion.pdf) and an accompanying instructional video (https://www.youtube.com/watch?v=BtkLFl3vim4). This manual is versioned and updatable as new information becomes available. Similar validations of scientific data used in forensic methods would support the ongoing effort to establish valid and reliable methods and protocols for proficiency testing, training, and certification.

**Specifications Table**

**Table:** 

Subject area	*Biological Anthropology*
More specific subject area	*Forensic Anthropology*
Type of data	*Tables and figures*
How data was acquired	*GPM spreading and sliding calipers from a small anthropometric kit, GPM mandibulometer, Paleotech laboratory osteometric board, cloth tape measure*
Data format	*Raw and examples of analyzed data*
Experimental factors	*Four repeated rounds of osteometric data was collected by four observers to quantify intraobserver and interobserver error of 99 measurements.*
Experimental features	*Two-way mixed ANOVAs and repeated measures ANOVAs with pairwise comparisons were run in SPSS 23. Relative and absolute technical error of measurement (TEM) was calculated in Microsoft Excel (Version 15.32).*
Data source location	*William M. Bass Donated Skeletal Collection, Department of Anthropology, University of Tennessee, Knoxville, TN, USA*
Data accessibility	*The raw data is published on Mendeley Data at*https://data.mendeley.com/datasets/6xwhzs2w38/1
	*Langley NR, Jantz RL, Meadows Jantz L, Maijanen H, McNulty S, Ousley SD. **DCP 2.0 Osteometric Data, Version 1. DOI:** 10.17632/6xwhzs2w38.1*

**Value of the data**

• These data were collected to quantify the reliability of osteometric data used in forensic anthropology research and case analyses.
• Examples of the data analysis are provided for anyone who desires to replicate the analyses on our raw data or on their own data.
• Similar validations of scientific data used in forensic methods would support the ongoing effort to establish valid and reliable methods and protocols for proficiency testing, training, and certification.
• This data analysis supported the dissemination of a free laboratory manual of revised osteometric definitions (*Data Collection Procedures 2.0* [1], pdf available at https://fac.utk.edu/wp-content/uploads/2016/03/DCP20_webversion.pdf) and an accompanying instructional video (https://www.youtube.com/watch?v=BtkLFl3vim4). This manual is versioned and updatable as new information becomes available.

## 1 Data

**Fig. 1:**
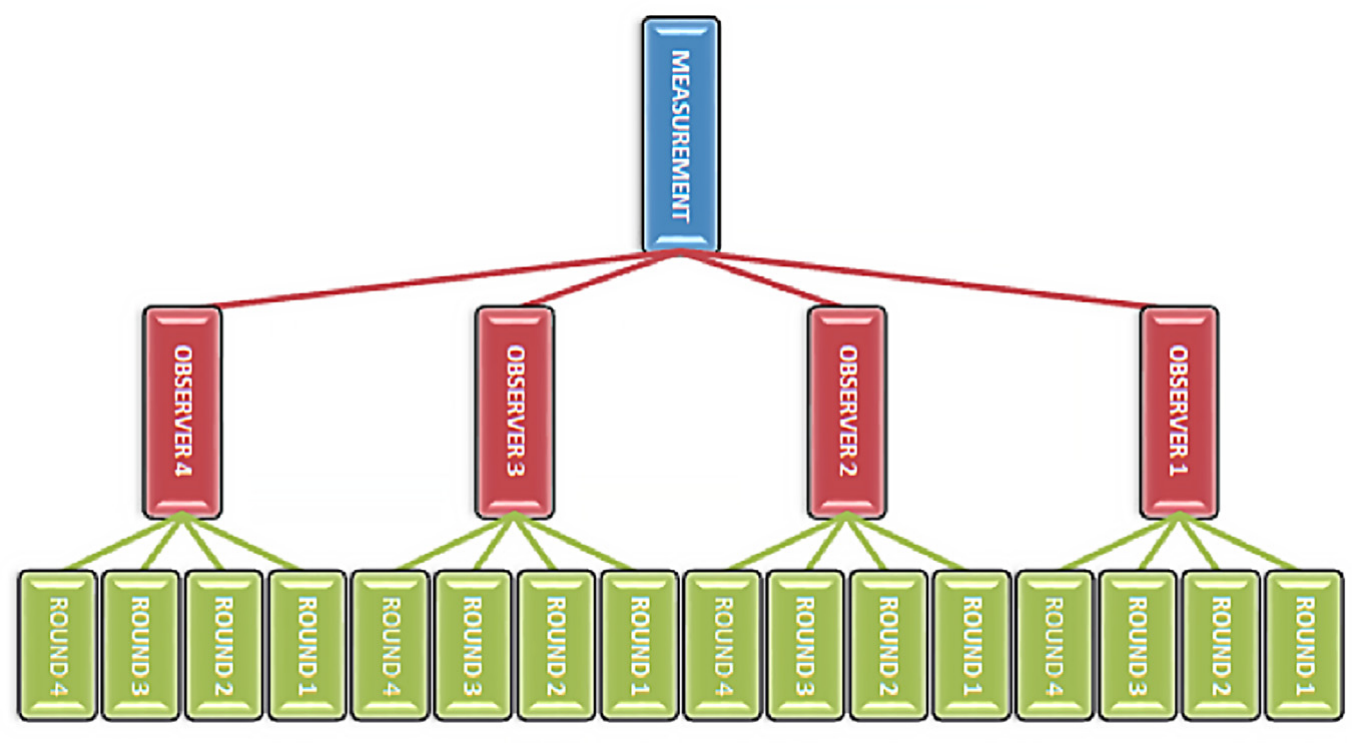
Schematic representation of data collection design for each measurement.

Osteometric data (n = 99 measurements) were collected on a random sample of William M. Bass Donated Collection skeletons (n = 50 skeletons). Four observers measured the left elements of 50 skeletons. After the complete dataset of 99 measurements was collected on each of the 50 skeletons, each observer repeated the process for a total of four rounds. Fig. 1 provides a schematic of the data collection design for each measurement (n = 99 measurements). Two-way mixed ANOVAs and repeated measures ANOVAs with pairwise comparisons were run to examine intraobserver and interobserver error, and relative and absolute technical error of measurement (TEM) was calculated for measurements with significant ANOVA results. The raw data is available on Mendeley Data (see Specifications Table).

## 2 Experimental design, materials and methods

Seventy-eight measurements (34 cranial and 44 postcranial) from *Data Collection Procedures for Forensic Skeletal Material, 3*^*rd*^ *edition* [2] were recorded by 4 observers on 50 William M. Bass Donated Collection skeletons from the following elements: cranium, mandible, clavicle, scapula, humerus, radius, ulna, femur, tibia, fibula, os coxa, sacrum, and calcaneus. Twenty-one additional measurements were also measured (see Supplementary Material), for a total of 99 measurements. The observers measured the left elements of 50 skeletons unless the left was unavailable, in which case the right side was substituted. The four observers were assigned numbers based on experience level, with Observer 1 (L) having the most experience (27 years) and Observer 4 (S) having the least experience (3 years); Observer 2 (H) had 14 years of experience, and Observer 3 (N) had 10 years. Measurements were taken on each skeleton using the instrument specified in the measurement definition in *Data Collection Procedures for Forensic Skeletal Material,* 3rd edition [2] (e.g. spreading calipers, digital sliding calipers, tape measure, osteometric board, mandibulometer). Once all 50 skeletons were measured, the process was repeated for a total of four rounds. Observers were provided copies of *Data Collection Procedures for Forensic Skeletal Material* [2] and *Cranial Variation in Man* [3]; the latter describes how to locate cranial landmarks if sutures are obliterated, Wormian or apical bones are present, etc. Instruments were calibrated with calibration rods before each measuring session, and the following conditions were modeled to establish the repeatability of the measurements according to the National Institute of Standards and Technology's *Guidelines for Evaluating and Expressing the Uncertainty of NIST Measurement Results* [4]:

1. The measurement procedure was performed the same each time.
2. The same observer performed each measurement with the same measuring instrument.

**Fig. 2:**
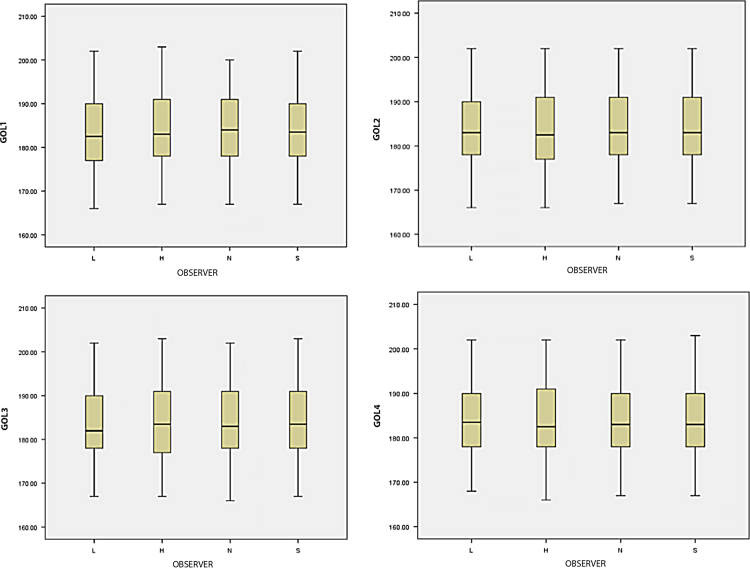
**Box and Whisker Plots.** Box and whisker plots for each measurement round of variable GOL used to screen the data for extreme outliers.

**Fig. 3:**
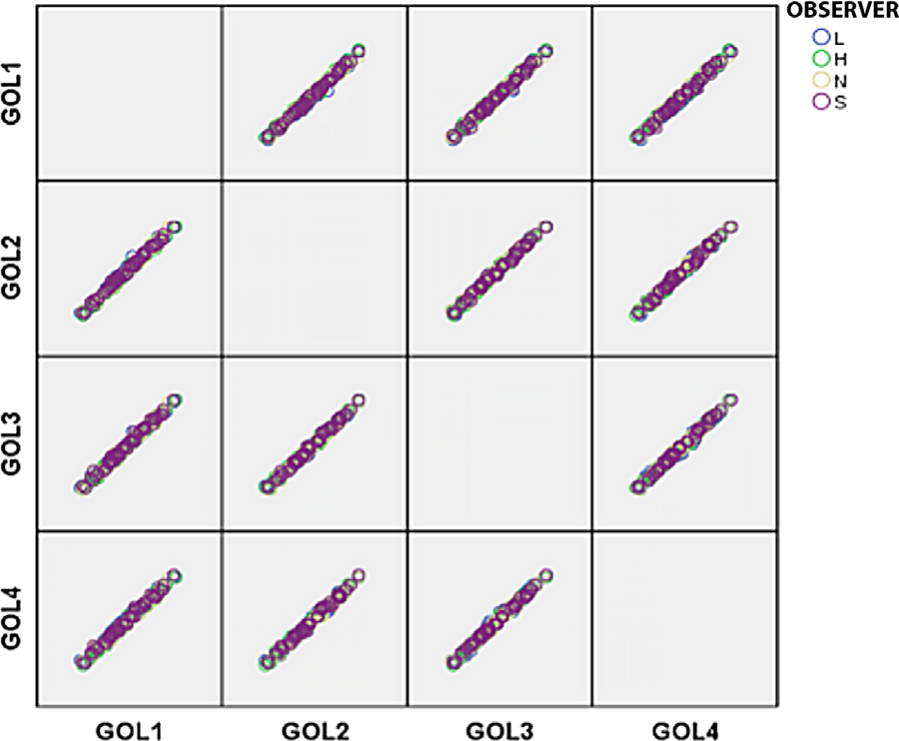
**Scatterplot Matrix for Variable GOL.** Used to examine data for extreme outliers.

**Fig. 4:**
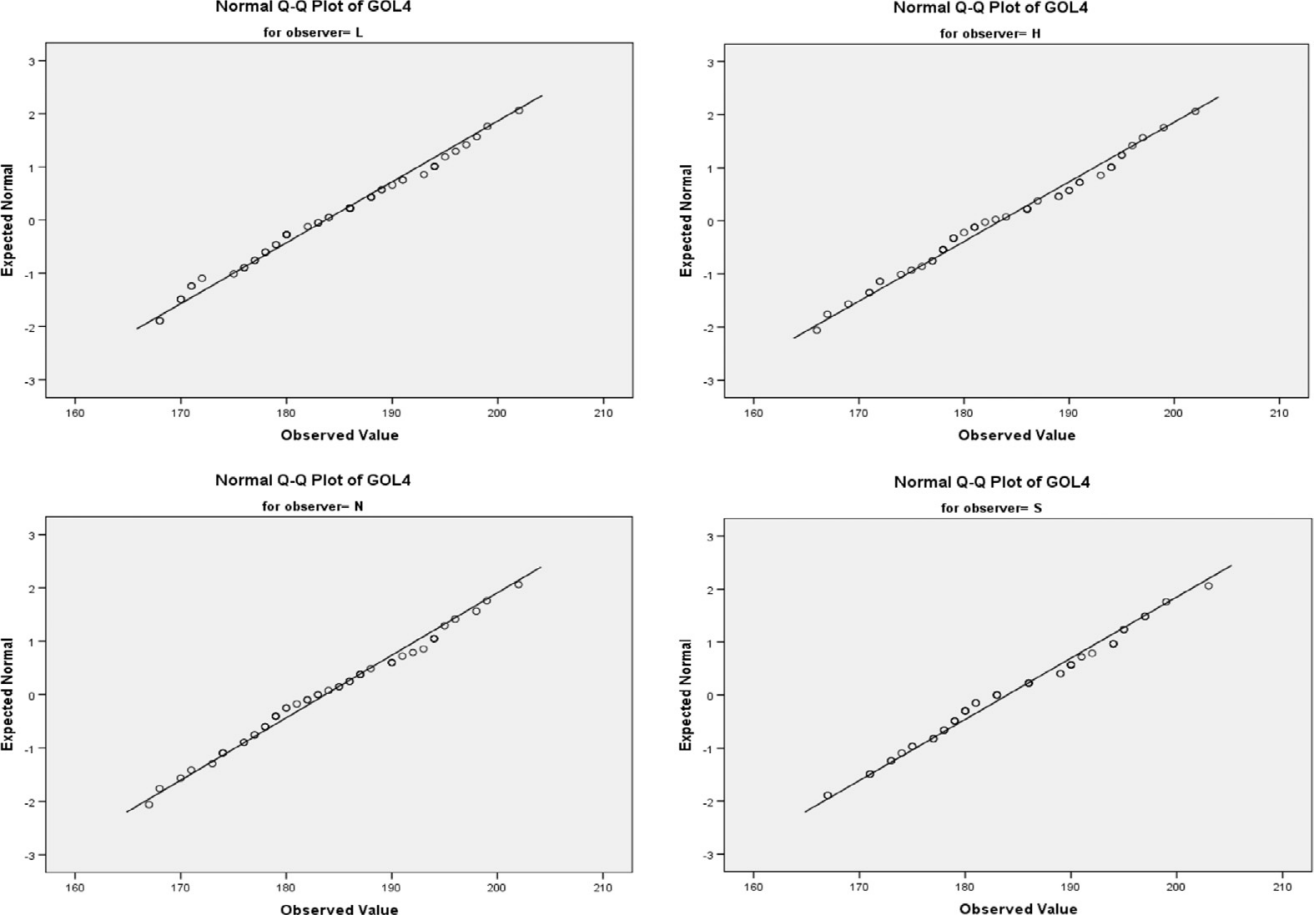
**Q-Q Plots of Variable GOL for Measurement Round #4.** Example of normally distributed data for variable GOL.

An example of the data analyses performed to evaluate and quantify observer error is provided for the variable GOL (maximum cranial length). Box and whisker plots, scatter plot matrices, and Q-Q plots were constructed to screen the data for extreme outliers and check for normality (Fig. 2, Fig. 3, Fig. 4). Each variable was checked for homogeneity of variances (Levene's test, Table 1) and the equality of covariance matrices (Box's test, Table 2). Two-way mixed ANOVAs and repeated measures ANOVAs were run in SPSS 23 [5] to examine intraobserver (within-subjects; factor=repeated measurements) and interobserver (between-subjects; factor=observer) variability [6]. Mauchly's test of sphericity was used to test the equality of variances between the within-subjects factors (e.g. repeated measures) and decide how to proceed with the ANOVA (Table 3).

**Table 1:** **Levene's Test of Homogeneity of Variances.** There was homogeneity of variances among the observer data for each round of data collection for the variable GOL (p > 0.05).

Measurement variable & (Measurement round)	F	df1	df2	Sig. (α = 0.05)
GOL(1)	.012	3	196	.998
GOL(2)	.077	3	196	.972
GOL(3)	.030	3	196	.993
GOL(4)	.061	3	196	.980

**Table 2:** **Box's Test of the Equality of Covariances**. There was homogeneity of covariances for the variable GOL across all groups, as assessed by Box's test (p=.063).

Box's M	44.283
F	1.422
df1	30
df2	105621.079
Sig. (α = 0.05)	.063

**Table 3:** **Mauchly's Test of Sphericity**. The assumption of sphericity was met for the variable GOL (p = .293), so a Greenhouse-Geisser correction is not needed.

Within subjects effect	Mauchly's W	Approx. chi-square	df	Sig. (α = 0.05)	Greenhouse-geisser
Observer	.969	6.140	5	.293	.980

Greenhouse-Geisser corrections were used for variables that failed Mauchly's test of sphericity. Simple main effects were run for variables with significant interactions between the between- and within-subjects factors (Table 4), and pairwise comparisons were examined for variables with significant main effects. Though no main effects were not significant for the variable GOL, the pairwise comparisons between observers and repeated measurement rounds are shown here as an example of this analysis (Table 5, Table 6). These comparisons are useful for examining patterns and elucidating if the issue is with one observer's interpretation of a measurement definition or if the error is more widely dispersed across the dataset, indicating potential problems with a landmark. Issues with a single observer may be remedied by clarifying a measurement definition; more widely dispersed issues may indicate that a measurement is more problematic and therefore unreliable.

**Table 4:** **Tests of Within- and Between-Subjects Effects for GOL**. There was no statistically significant effect of repeated measurements (i.e. intraobserver variation) for the variable GOL (p = .698) and no statistically significant difference between observers (i.e. interobserver variation) for the variable GOL (p = .993).

**Within-Subjects Effects**					
Source	Type III sum of squares	df	Mean square	F	Sig. (α = 0.05)
Repeated measurement (GOL)	.550	3	.183	.477	.698
**Between-Subjects Effects**					
Observer	26.020	3	8.673	.029	.993

**Table 5:** **Pairwise Comparisons of Measurement Rounds.** P-values adjusted for multiple comparisons using a Bonferroni adjustment. There is no statistically significant difference between observers for the measurement GOL.

Observer		Mean difference	Std. error	Sig. (α = 0.05)	95% Confidence interval for difference		
					Lower bound		Upper bound
L	H	−.0300	1.74146	1.000	−4.5425		4.4825
	N	−.3700	1.74146	.997	−4.8825		4.1425
	S	−.3800	1.74146	.996	−4.8925		4.1325
H	L	.0300	1.74146	1.000	−4.4825		4.5425
	N	−.3400	1.74146	.997	−4.8525		4.1725
	S	−.3500	1.74146	.997	−4.8625		4.1625
N	L	.3700	1.74146	.997	−4.1425		4.8825
	H	.3400	1.74146	.997	−4.1725		4.8525
	S	−.0100	1.74146	1.000	−4.5225		4.5025
S	L	.3800	1.74146	.996	−4.1325		4.8925
	H	.3500	1.74146	.997	−4.1625		4.8625
	N	.0100	1.74146	1.000	−4.5025		4.5225

**Table 6:** **Pairwise Comparisons of Measurement Rounds.** P-values adjusted for multiple comparisons using a Bonferroni adjustment. There is no statistically significant difference between repeated measurement rounds.

					95% Confidence interval for difference	
Measurement round		Mean difference	Std. error	Sig. (α = 0.05)	Lower bound	Upper bound
1	2	−.010	.061	1.000	−.172	.152
	3	−.045	.066	1.000	−.220	.130
	4	−.065	.066	1.000	−.240	.110
2	1	.010	.061	1.000	−.152	.172
	3	−.035	.057	1.000	−.188	.118
	4	−.055	.061	1.000	−.218	.108
3	1	.045	.066	1.000	−.130	.220
	2	.035	.057	1.000	−.118	.188
	4	−.020	.061	1.000	−.183	.143
4	1	.065	.066	1.000	−.110	.240
	2	.055	.061	1.000	−.108	.218
	3	.020	.061	1.000	−.143	.183

Absolute and relative technical error of measurement (TEM) was calculated to quantify observer error. TEM was calculated to examine the variability among a single observer repeating a measurement multiple times (e.g. repeatability or intraobserver error), as well as the variability between multiple observers (interobserver error). Absolute TEM is calculated as

$$\sqrt{\frac{\sum_{1}^{N}\left[\sum_{1}^{K}{M(n)}^{2}-\frac{{\left(\sum_{1}^{K}M(n)\right)}^{2}}{K}\right]}{N(K-1)}}$$

where N is the sample size (N=50 skeletons), K is the number of observers or the number of repeated rounds per observer (K=4), M is the measurement, and M(n) is the n^th^ repetition of the measurement [7]. Relative TEM is calculated by dividing absolute TEM by the mean and multiplying by 100. Relative TEM is a measure of precision (or imprecision) unaffected by scale or sample size that allows for the direct comparison of measurements of different scales [7], [8]. Acceptable ranges for the relative, or percent, TEM in anthropometry are <1.5% for intra-examiner error and <2% for inter-examiner error [8]. To calculate intraobserver relative TEM, the relative TEM was calculated for the four measurement rounds on one skeleton, and the average of the 50 relative TEM values was used as the relative TEM. To calculate interobserver relative TEM, relative TEM was calculated for each measurement round using the data from all four observers; the mean relative TEM from all four rounds was used as the relative TEM. The Supplementary Materials file TEM CALCULATION EXAMPLES.xls provides an example of TEM calculations for the variable GOL (the Excel workbook contains one spreadsheet for intraobserver TEM and one spreadsheet for interobserver TEM calculations).
