# Supplementary material for: Data for validation of osteometric methods in forensic anthropology
Source: Data Brief. 2018 May 7;19:21–8. doi: 10.1016/j.dib.2018.04.148 (PMC5992973; doi:10.1016/j.dib.2018.04.148)
Supplement: Supplementary file 3 — Supplementary material [file mmc3.docx]

Table 1. Additional Measurements. These measurements are not included in the 3^rd^ edition of *Data Collection Procedures* but were evaluated in this study as alternatives to potentially error-prone measurements and/or useful additions to current standards.

CRANIUM

1. **Biasterion breadth (ASB)**: the distance between right and left asterion. Asterion is a landmark at the junction of lambdoidal, parietomastoid, and occipitomastoid sutures. (Howells 1973; Martin and Knussmann 1988)

2. **Zygoorbitale breadth (ZOB)**: the distance between right and left zygoorbitale. Zygoorbitale is a landmark at the junction of the zygomatic bone and the maxilla (i.e. the zygomatico-maxillary suture) at the orbital border. (Jantz and Ousley 2005)

1. **Bimaxillary breadth (ZMB)**: The breadth across the maxillae, from the left to right zygomaxillare anterior (zma). The endpoints of the measurement are located on the facial surface and not on the inferior aspect of the zygomaxillary suture. (Howells 1973)

4. **Mastoid height (MDH)**: The direct distance between porion and mastoidale. Place the fixed arm of the caliper on porion and move the movable arm until it touches mastoidale. This may be most easily accomplished by holding the calipers in a coronal plane. (Jantz and Ousley 2005)

CLAVICLE

5. **Maximum diameter at midshaft**: *Instrument*: sliding caliper. Find midshaft using an osteometric board, mark with a pencil, and rotate calipers around the shaft until the maximum is reached. This is frequently found in the sagittal dimension. (Shirley 2009)

6. **Minimum diameter at midshaft**: *Instrument*: sliding caliper. Find midshaft using an osteometric board, mark with a pencil, and rotate calipers around the shaft until the minimum is found. (Shirley 2009)

SCAPULA

7. **Glenoid Cavity Breadth**: *Instrument:* sliding caliper. Taken at a point just below the constriction of the ventral border. Measured across the breadth of the glenoid cavity from the ventral to the dorsal margin. (Corruccini and Ciochon 1976)

8. **Glenoid Cavity Height**: *Instrument:* sliding caliper. Taken from the superior to the inferior margin of the glenoid cavity, being sure that the measurement is taken perpendicular to glenoid cavity breadth. (Corruccini and Ciochon 1976)

RADIUS

9. **Maximum diameter at midshaft**: *Instrument*: sliding caliper. Find midshaft using an osteometric board, mark with a pencil, and rotate calipers around the shaft until the maximum is found.

10. **Minimum diameter at midshaft**: *Instrument*: sliding caliper. Find midshaft using an osteometric board, mark with a pencil, and rotate calipers around the shaft until the maximum is found.

11. **Maximum Diameter of the Head**: *Instrument:* sliding caliper. Taken from a point on the edge of the articular surface of the bone across to the opposite side. The bone is rotated until the maximum distance is obtained. (Trotter and Gleser 1952)

ULNA

12. **Maximum Diameter at Midshaft**: *Instrument*: sliding caliper. Find midshaft using an osteometric board, mark with a pencil, and rotate calipers around the shaft until the maximum is found.

13. **Minimum Diameter at Midshaft**: *Instrument*: sliding caliper. Find midshaft using an osteometric board, mark with a pencil, and rotate calipers around the shaft until the minimum is found.

14. **Maximum Breadth of the Olecranon Process**: *Instrument:* sliding caliper. Measured from the medial and lateral margins of the olecrenon process’ articular surface at its greatest breadth. (McHenry et al. 1976)

15. **Minimum Breadth of the Olecranon Process**: *Instrument:* sliding caliper. Measured from the medial and lateral margins of the olecrenon process’ articular surface where the constriction on the medial margin becomes apparent. (Zobeck 1983)

16. **Olecranon Process to Coronoid Process Length**: *Instrument:* sliding caliper. From the most anteriorly projecting point on the olecrenon process to the peak of the coronoid process. (McHenry et al. 1976)

FEMUR

17. **Anterior-Posterior Diameter of the Lateral Condyle**: *Instrument:* sliding caliper. The projected distance between the most posterior point on the lateral condyle and lip of the patellar surface taken perpendicular to the axis on the shaft. (Montagu 1960)

18. **Anterior-Posterior Diameter of the Medial Condyle**: *Instrument:* sliding caliper. The projected distance between the most anterior point on the joint surface and the most posterior point on the medial condyle and the lip of the patellar surface taken perpendicular to the axis of the shaft. (Montagu 1960)

SACRUM

19. **Anterior-Posterior Diameter of S1**: *Instrument:* sliding caliper. Maximum possible diameter of the first sacral vertebra measured by taking one point on the antero-superior border and the other point on the postero-superior border. (Mishra et al. 2003)

TIBIA

20. **Maximum Diameter at Midshaft**: *Instrument*: sliding caliper. The maximum diameter of the tibial shaft at midshaft. This measurement is instrumentally determined but usually located in the anterior-posterior orientation.

21. **Minimum Diameter at Midshaft**: *Instrument*: sliding caliper. The minimum diameter of the tibial shaft at midshaft. This measurement is instrumentally determined but usually located in the medial-lateral orientation.
