# Supplementary material for: Data for validation of osteometric methods in forensic anthropology
Source: Data Brief. 2018 May 7;19:21–8. doi: 10.1016/j.dib.2018.04.148 (PMC5992973; doi:10.1016/j.dib.2018.04.148)
Supplement: Supplementary file 4 — Supplementary material [file mmc4.pdf]

DATA COLLECTION PROCEDURES FOR FORENSIC SKELETAL MATERIAL 2.0

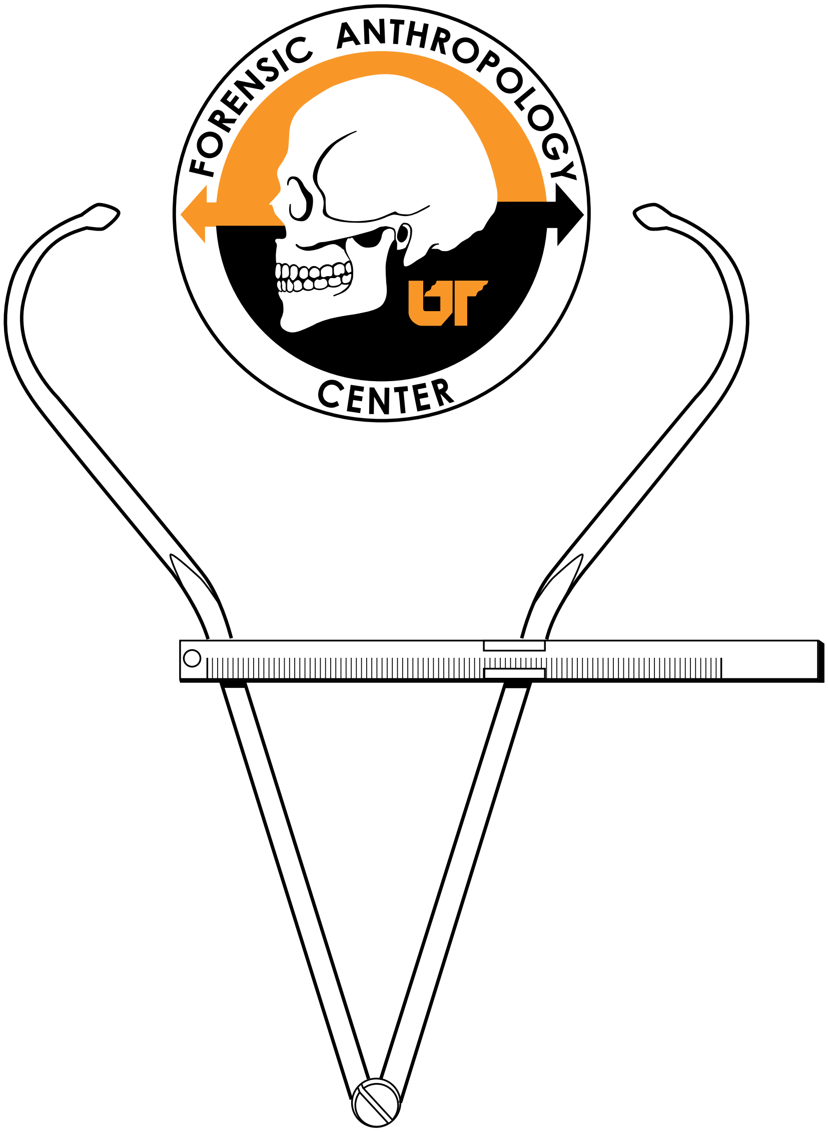

Natalie R. Langley
Lee Meadows Jantz
Stephen D. Ousley
Richard L. Jantz
George Milner

DATA COLLECTION PROCEDURES FOR FORENSIC SKELETAL MATERIAL 2.0

Forensic Anthropology Center
Department of Anthropology
The University of Tennessee
Knoxville, Tennessee

Anatomy Department
Lincoln Memorial University
DeBusk College of Osteopathic Medicine

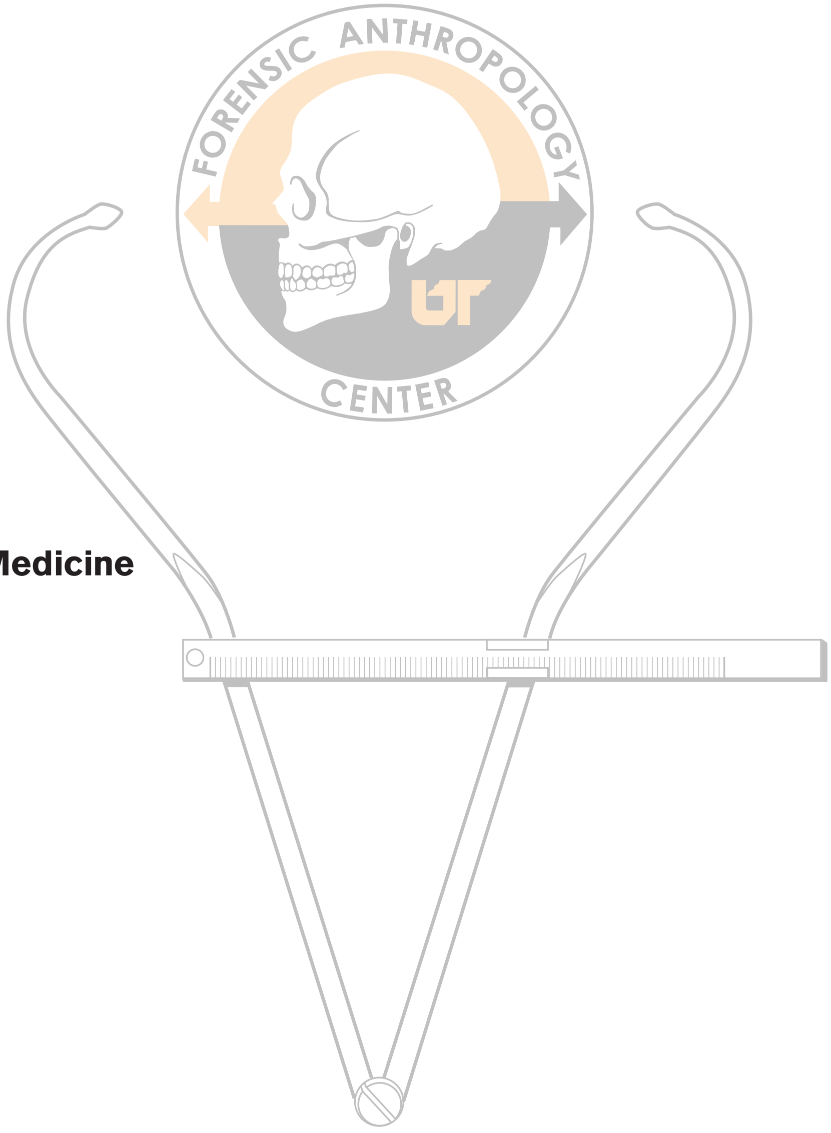

2016

Natalie R. Langley
Lee Meadows Jantz
Stephen D. Ousley
Richard L. Jantz
George Milner

Forensic data bank forms are included in Appendix A. Once completed, the forms in association with any additional information provided should be emailed/mailed to Richard Jantz at the following address. Data may also be submitted digitally using the Fordisc program.

Cover design, interior layout, and line illustrations by Neil O. Ward Designs. Workbook set in Akzidenz Grotesk.

Department of Anthropology
The University of Tennessee
250 South Stadium Hall
Knoxville, TN 37996-0720
Office: 865-974-4408
FAX: 865-974-2686
E-Mail: rjantz@utk.edu

TO ORDER WORKBOOK CONTACT:
The University of Tennessee
Forensic Anthropology Center
252 South Stadium Hall
Knoxville, TN 37996-0729

Phone: (865) 974-4408

Table of Contents

6. PREFACE
- 7 Preface to Data Collection Procedures 2.0
- 9 Preface to Data Collection Procedures, 3rd edition
- 10 Preface to Data Collection Procedures, 2nd edition
- 11 Preface to Data Collection Procedures, 1st edition
- 12 DCP 2.0: Amendments to Data Collection Procedures, 3rd Edition

14. INTRODUCTION

16. SECTION 1 : EPIPHYSEAL UNION
- 17 Epiphyseal closure

18. SECTION 2 : TRANSITION ANALYSIS
- 21 **Pubic symphysis**
  - 22 Symphyseal relief
  - 25 Dorsal symphyseal texture
  - 27 Superior protuberance
  - 30 Ventral symphyseal margin
  - 34 Dorsal symphyseal margin
- 37 **Sacroiliac joint**
  - 38 Superior demiface topography
  - 40 Inferior demiface topography
  - 42 Superior surface characteristics
  - 44 Middle surface characteristics
  - 46 Inferior surface characteristics
  - 48 Inferior surface texture
  - 49 Superior posterior iliac exostoses
  - 51 Inferior posterior iliac exostoses
  - 53 Posterior exostoses
- 55 **Cranial sutures**
  - 56 Coronal pterica, Sagittal obelica, Lambdoidal asterica, Zygomaticomaxillary
  - 59 Interpalatine

60. SECTION 3 : FORENSIC MEASUREMENTS
- 61 Definition of cranial landmarks
- 65 Cranial measurements
- 71 Mandibular measurements
- 73 Postcranial measurements

83. REFERENCES

87. CASE INFORMATION

88. GENERAL INFORMATION

90. INVENTORY

91. APPENDIX A - FORENSIC RECORDING FORMS
Forensic recording forms, Forensic case information, Forensic inventory, Forensic morphological observations, Forensic measurements

99. APPENDIX B - DENTAL RECORDING FORMS
Dental chart, Decidious dental record

102. APPENDIX C - MEASUREMENT ERROR TABLES
Measurement Error Tables

PREFACE

Data Collection Procedures For Forensic Skeletal Material 2.0

PREFACE TO DATA COLLECTION PROCEDURES FOR FORENSIC SKELETAL MATERIAL 2.0

The *Data Collection Procedures (DCP)* manual has served the forensic anthropology community since its inception in 1986. A grant from the National Institute of Justice (85-IJ-CX-0021) provided the funds to begin the work, resulting in the first edition of the DCP. The DCP established a means to amass a centralized data base of skeletal data on modern humans (the Forensic Data Bank, or FDB). These data provided the basis for deriving standards to determine age, sex, ancestry, and stature of unknown remains. Ultimately, the FDB became the reference database for the Fordisc software program used to estimate sex, ancestry, and stature. As of this writing, the FDB has just over 4,000 cases.

The DCP recording forms have served as a means of submitting cases to the FDB since the publication of the first edition, and many practitioners use the manual as a laboratory reference guide for osteometric definitions. The last update to the DCP was the third edition, released in 1994. Fordisc 3 was released in 2005. The most recent version of Fordisc is 3.1.307 (October 16, 2015), and the software is updated regularly as the reference sample increases. The 20-year interval since the DCP was last updated and the most recent Fordisc version led to an accumulation of inconsistencies between the DCP forms and the osteometric data used by Fordisc. The next release of the software will include an age estimation module, which will not be derived from the information in the DCP third edition. All of these factors, plus a growing concern for the reliability and repeatability of some of the FDB measurements, provided the impetus for a *substantial* revision of data collection protocols.

The National Institute of Justice funded the research effort that led to the significant revisions herein (Grant Number 2013-DN-BX-K038). Four observers took the 78 standard measurements (34 cranial and 44 postcranial) from *Data Collection Procedures for Forensic Skeletal Material, 3rd edition* plus 20 additional measurements on a sample of 50 William M. Bass Donated Collection skeletons with standard osteometric equipment (sliding calipers, spreading calipers, an osteometric board, and a mandibulometer). The skeletons were measured four times by each observer. Observers measured all 50 skeletons once, then repeated a second round, followed by a third and fourth. Repeated measures ANOVA, scaled error index, and technical error of measurement were used to evaluate reliability and repeatability of the measurements. The results pinpointed a number of measurements requiring close examination of the landmarks and/or definitions. The new osteometric data protocols in DCP 2.0 are the product of this investigative effort.

Data Collection Procedures 2.0 is the first version of many to come. The DCP will be versioned, like the Fordisc software. Changes in newly released versions will be detailed in the introductory section of the manual. The DCP will correspond with the Fordisc software so that practitioners may use the manual in the field to collect data, seamlessly input the data into the software, and then send the data to the FDB (either via Fordisc or the DCP recording forms). The DCP is also accompanied by an instructional video. Links to the video and manual are available on the University of Tennessee's Forensic Anthropology Center webpage and on the Fordisc page of Mercyhurst Archaeological Institute's website. The results of the observer error calculations are available in Appendix C.

The amendments contained in the DCP 2.0 are listed at the beginning of this manual. They include changes to the measurements, references, and age estimation section. The age estimation materials were provided by George Milner and are designed to correspond to the Transition Analysis method incorporated in the next iteration of the Fordisc software program. Line drawings have been simplified and streamlined, and obvious measurements have been removed from figures. We urge the user to read the definitions and not rely solely on illustrations. The accompanying video is also a useful resource. In keeping with tradition we have included the prefaces to the first three editions of the DCP, as they provide an interesting history.

Adams and Byrd (2002) recognized the importance of this type of contribution in their study of measurement error in select postcranial measurements, observing that:

1. Procedures using skeletal measurements should favor measurements that are relatively easy to take.
2. Clear definitions of the measurements should be provided in any publications.
3. Problematic measurements such as pubis length are invalid due to the problem of locating a particular landmark (i.e. the center of the acetabulum); these measurements should not be used in analyses.
4. University training in osteometrics promotes continuity in data collection. Beyond the university, forensic laboratories should include detailed measurement descriptions in their standard operating procedures and provide osteometric training to new staff.
5. On account of the significant implications that the results

of these metric analyses hold (e.g. the identification or exclusion of an unknown individual), ***it is of utmost importance that measurements used by forensic anthropologists can be accurately and reliably taken and that they are replicable between observers***.

6. Significant interobserver measurement variation could compromise pooled datasets compiled from multiple researchers and, in turn, bias research based on these data. Interobserver error in reference data (such as that in the Forensic Data Bank) will increase the standard error and introduce potential bias in models to estimate stature, sex, or ancestry.

Ultimately, *DCP 2.0* addresses demands put forward by the National Academy of Sciences (NAS) Report *Strengthening Forensic Science in the United States: A Path Forward* (2009): forensic disciplines must critically evaluate, validate, and establish error rates for methodologies commonly used to produce material for forensic case reports and conduct "rigorous systematic research to validate the discipline's basic premises and techniques" (p. 22). Many of the basic techniques in forensic anthropology are based on osteometric data., and this manual utilizes osteometric data that has been tested for repeatability and reliability.

An undertaking of this magnitude prompts us to thank many individuals. Heli Maijanen and Shauna McNulty tirelessly collected osteometric data. We are also grateful to Charlene Weaver in the University of Tennessee Anthropology Department and Carolyn Gulley and Melissa Miracle in the Lincoln Memorial University grants office for their assistance with administrating the grant. A huge thanks to Neil Ward, the graphic artist who did line drawings from bones and bone images, designed the layout of the manual, and met with us countless times in the dingy, poorly lit, and sometimes cold depths of Neyland Stadium. We appreciate his patience, creativity, and diligence. We also thank the unnamed individuals who donate their remains to the Forensic Anthropology Center and to the persistent practitioners who submit data to the Forensic Data Bank. We welcome your comments and questions as we move forward with this effort.

PREFACE TO DATA COLLECTION PROCEDURES, THIRD EDITION

The Forensic Data Bank at the University of Tennessee, Knoxville, now has information from over 1200 cases. Almost 900 of these are of certain race and sex. We believe that this guide and the accompanying forms are a sound basis for your own data collection as well as for contributions to the Forensic Data Bank. Analyses using this data base have been presented at the AAFS meetings (Meadows and Jantz 1992; Ousley and Jantz 1992, 1993; Willcox *et al* 1992; Marks 1990; Moore-Jansen 1991). Most of the discriminate functions from Ousley and Jantz (1992, 1993) are found in Bennett (1993) and are also available upon request. Data from the Forensic Data Bank helped in the identification of remains from Operation Desert Storm and the Branch Davidian compound in Waco, Texas. The Forensic Data Bank is the foundation for FORDISC 1.0 (Jantz and Ousley 1993), a computer program that generates custom discriminant functions based on available cranial measurements. FORDISC 2.0 will include postcranial race (Black vs. White) and sex functions and stature estimation procedures based on the latest information (Meadows and Jantz 1992; Jantz 1994; Ousley 1994).

Recent works have questioned the metric and morphological standards derived from 19th century collections such at the Terry and Hamann-Todd collections when applied to modern Americans (Ayers *et al.* 1989; Erickson 1982; Jantz and Moore-Jansen 1988, 1992; Meadows and Jantz 1992; Murray 1990; Ousley and Jantz 1992, 1993). The skeletal biology of Americans is changing due to secular changes, migrations, and gene flow. In the absence of large modern collections, a data bank is necessary to keep pace with the changing U.S. population. A data bank does naturally impose certain limits on research, since all possible data cannot be collected. But it does preserve data that would otherwise be lost when buried, and has the added possibility of including individuals from around the country and arond the world. We hope that you will share your case information with us so that we can assemble more comprehensive data sets and dvelop better methods of identifying skeletal materials. Sales of FORDISC and this manual have enabled data collection from several widespread locations.

In this edition of the manual, we have changed landmarks used for orbit breadth, biorbital breadth, and interorbital breadth. For orbital measurements, we now use dacryon and a definition of ectoconchion for Howells (1973) because these are easier to find and measure from. We have added more measurement tips and tried to make the measurement descriptions more clear. For example, *Sagittal* in many cases has been changed to *Anterior-Posterior*.

The vast majority of cases have come from our own efforts, though we thank especially Douglas Ubelaker and Ted Rathbun for continuing to contribute many cases. We also thank Michael Pietrusewsky, who contributed data from Vietnamese and Chinese individuals. We are especially grateful to William M. Bass for his continued enthusiastic support of the Forensic Data Bank.

PREFACE TO DATA COLLECTION PROCEDURES, SECOND EDITION

The development of a National Forensic Data Base at the University of Tennessee has been under way for six years and is now a significant resource for skeletal biology research. Our purpose was to assemble a large enough data base which could provide a significant research data base for forensic anthropology. Once established, the data base would be maintained and increased by annual contributions of new case reports/data records from practitioners of forensic anthropology across the nation.

Our initial task was to standardize recording procedures and general recording formats. We feel the present manuscript has accomplished this. Our protocol is not meant to limit anyone from additional or alternate procedures. It has, however, established a standardized "core" data set which will be available to researchers upon request. A computerized data entry format was designed and distributed to several institutions who are currently applying it to their personal or institutional data collecting. It is currently under revision. It should be noted that the protocol (Moore-Jansen and Jantz 1986) was submitted to all members of the Physical Anthropology section of the American Academy of Forensic Sciences for comments. The present edition is the result of our efforts to address resulting comments and questions.

Our major objective, yet to be fully acheived, is the participation of all practitioners and potential researchers in the field of forensic anthropology. At present the data base exceeds 850 records, of which about 60% are documetned forensic cases. All individuals were born during the 20th century and the vast majority died after 1960. To maintain and improve the data base, we rely entirely on the cooperation of those who observe and record forensic cases. In the last few years, contributions have been less than 30 per year. Exceptions include a one-time contribution of Larry Angel's forensic cases. Contributions have come from around the country, with major contributions coming from the Smithsonian Institution, Washington D.C., the Human Indentification Lab at the University of Arizona in Tuscon, and the Forensic Anthropology Center at the University of Tennessee, Knoxville.

At Knoxville, the main goal of our data colelction effort is to develop new identification methods for skeletal remains. We have already developed a discriminant function program for cranial and postcranial data (Moore-Jansen and Jantz 1989). We have also developed custom discriminant functions on several occasions to help forensics specialists identify remains. By Fall 1991, we also plant to make available a customizable discriminant function program that will run on any IBM compatible computer. To aid our efforts in computer-aided anthropology, the department of Anthropology at UT will soon purchase an 80386 computer, Paradox 3.0 database software, PC SAS statistical analysis software, and several programming languages packages. These hardware and software modernizations will help us to develop and process statistical analyses more rapidly.

In order to enhance these results and continue to improve the research facility that is now firmly established for forensic anthropology, we must encourage more contributions to the project as well as request for data. We are certain that the more the data is shared, the more practitioners will appreciate the importance of contributing to the data base. Up to now, virtually all research using the data has been conducted at the University of Tennessee. We are currently investigating various ways of making information available to researchers via modem, BITNET, and other electronic means.

With the help and guidance of a large number of individuals, we have established a significant and useful source of data, and feel that the success and enthusiasm for the project will continue. We wish to extend our deepest gratitude to all those who have participated in the data bank project over the past several years through suggestions and contributions. We wish again to acknowledge some of those people by name. We are especially indebted to William M. Bass for his financial support and encouragement since the project was first conceived. We are most grateful to Douglas H Ubelaker who has provided comments and suggestions for the project and contributed a great many case records. We especially appreciate his application and testing of our research conclusions in skeletal identification. We extend our sincere thanks to Judy Suchey and Michael Hoffman who responded immediately to our request for comments and questions on the previous edition. We thank Judy Suchey and Margaret Burch for providing us with copies of the templates for again pubic symphysis.

PREFACE TO DATA COLLECTION PROCEDURES, FIRST EDITION

The notion od systematically collecting information from forensic cases has been talked about for several years. Central to this idea is that data obtained from forensic cases would provide a more adequate basis for deriving standards, primarily for race, stature, sex and age determination, than the anatomical collections upon which many of the current standards rest. Accordingly, a committee was appointed under the Finnegan administration of the Physical Anthropology section of the American Association of Forensic Sciences consisting of Clyde Snow chair, Dick Jantz, Stan Rhine and Larry Angel. This committee met during the AAFS meeting in Orlando to discuss feasibility and direction in general terms. The committee (now consisting of Dick Jantz, chair, Stan Rhine, Larry Angel and Doug Ubelaker) met the following year in Cincinnati and drew up a tentative list of measurements and observations that might be incorporated into a forensic data bank.

At this point it became apparent that we would require funding. Bill Bass provided some start-up funds from the Department of Anthropology, University of Tennessee, enabling us to hire Peer H. Moore-Jansen, a Ph.D. student. Together we prepared a proposal to be submitted to the National Institute of Justice in their unsolicited research program. The proposal was funded and the project officially began September 1, 1986. The grant contains funds to purchase computer hardware and software and salary to support data base design and computerization. Peer Moore-Jansen has assumed the major responsibility for developing the protocol set forth here.

The present document is aimed at the recording of skeletal material and soft tissue cases of all ages, although specific instructions for recording young children are currently lacking. Until such guidelines are provided, we encourage everyone who chooses to follow the guidelines presented here, to apply them appropriately in each case as outlined in the instructions.

We have attempted to include measurements and observations which can readily be made on skeletonized remains and which are more or less standard. We have used Martin (1956;1957) as the reference for defining landmarks and measurements; the definitions which appear here are our translations. We have discovered that the definitions given in Singh and Bhasin (1968) are also essentially translations from Martin. Observations on age changes have been keyed to a variety of sources and are explained in the text.

The present document is a revised version of a preliminary draft distributed at the 1986 AAFS meeting in New Orleans. Although we regard the present document as a relatively permanent document it is certainly open to future revisions as they are deemed appropriate. Two important areas which are currently being considered for future revisions include 1) a more specific approach for recording skeletal data for young children, and 2) expansion of dental recording procedures.

The basic idea is that information available from forensic cases will be recorded on the data sheets and returned to us for entry onto the computerized data bank. We are prepared to make the information available to anyone who contributes. The information can be made available in a variety of forms appropriate to specific computer facilities.

We thank the members of the committee, Stan Rhine, Doug Ubelaker and the late Larry Angel for reading and commenting on a previous draft of this manuscript. Thanks also to Judy Myers Suchey, Sheilagh Brooks, Diane France, Frank Saul, P.S. Willey, Steven A. Symes and Henry W. Case for reading earlier drafts and providing comments. We are especially grateful to William Bass for providing support, both monetary and moral, which led to the realization of this project. The National Institute of Justice, grant number 85-IJ-CX-0021, provided the funds and we are indebted to Mr. Joe Kochanski for his help. Finally, our special gratitude goes to Ms. Kim Johnson who devoted herculean efforts to the typing and arrangement of this manuscript and to Mary Joe Hinton, who typed the revisions and additions to the second edition. FIgures 1,2,6,7, and 12-17 are reproduced with the permission of Alan R. Liss, Inc., New York. Figures 4 and 5 are reproduces courtesy of M. Yasar Iscan and Susan R. Loth. Figure 8 is reproduced courtesy of Jusy Myers Suchey and Margaret Burch. Appendix B is reproduced with minor modifications courtesy of Walter Birkby.

AMENDMENTS

DCP 2.0: Amendments To Data Collection Procedures, 3rd Edition

On account of the extensive changes in DCP 2.0 this list is not exhaustive. However, it is fairly detailed and calls the user's attention to pertinent issues.

The manual is arranged in 3 sections:

1. **EPIPHYSEAL UNION**
2. **TRANSITION ANALYSIS**
3. **FORENSIC MEASUREMENTS**

The age estimation section has been changed completely:

- Cranial sutures removed
- Suchey-Brooks, McKern and Stewart, Gilbert and McKern, and Todd pubic symphysis removed
- Lovejoy auricular surface removed
- Iscan ribs removed
- Dorsal pubic pitting omitted
- Transition Analysis aging method added

Osteometric definitions come from Martin and Knussman (1988) and Howells (1973).

CRANIAL LANDMARKS:

- Alare removed (this is a soft tissue landmark)
- Asterion added
- Auriculare replaced with radiculare. The latter is the true point to which the measurement AUB is taken. Martin and Knussman (1988) note that it is the point that Howells (1973) uses for AUB. However, auriculare is located on the lateral portion of the temporal superior to the external acoustic meatus (Martin and Knussman 1988). We have therefore deleted auriculare and added radiculare.
- Basion definition changed
- Bregma definition expanded for clarification
- Dacryon definition changed
- Ectoconchion definition expanded for clarification
- Lambda definition expanded for clarification
- Mastoidale added (used for MDH)
- Nasion clarified (nasion is on the frontal bone)
- Nasospinale removed (this landmark is no longer used for NLH)
- Porion added (used for MDH)
- Prosthion changed (anterior prosthion from Howells)
- Zygomaxillare anterior added
- Zygoorbitale added

CRANIAL MEASUREMENTS:

- Nasio-occipital length (NOL) added
- BBH changed
- BNL changed
- BPL changed
- Landmarks for AUB changed to radiculare (the measurement has not changed, only the name of the landmark)
- NPH: use anterior prosthion
- NLH definition changed (no longer using nasospinale—please read revised definition carefully)
- NLB changed (no longer defined with alare)
- OBB: note definition of dacryon
- DKB: note definition of dacryon
- FOL definition changed (no longer uses basion)
- MDH changed (read definition carefully; also read definitions of porion and mastoidale)
- ASB added
- ZMB added
- ZOB added

MANDIBULAR MEASUREMENTS

- Minimum ramus breadth changed (Martin #71 to Martin #71a; no longer taken perpendicular to maximum ramus height)
- Maximum ramus breadth deleted

POSTCRANIAL MEASUREMENTS

- Clavicle:
  - Sagittal and vertical diameters at midshaft replaced with maximum and minimum midshaft diameters
- Scapula:
  - Two measurements of the glenoid cavity added: glenoid cavity breadth and glenoid cavity height.
- Radius:
  - Sagittal and transverse midshaft diameters changed to minimum and maximum midshaft diameters
  - Maximum diameter of the radial head added
- Ulna:
  - Dorso-volar and transverse diameters changed to maximum and minimum midshaft diameters
  - Physiological length definition clarified (proximal measurement is taken from the guiding ridge)
  - Olecranon breadth added

POSTCRANIAL MEASUREMENTS

- Sacrum:
  - Transverse diameter of S1 clarified
  - AP Diameter of S1 added
- Innominate:
  - Innominate height and breadth now specified as maxima
  - Added: minimum iliac breadth, maximum pubis length, minimum pubis length, ischial length, minimum ischial length, maximum ischiopubic ramus length, ASIS to symphysion, PSIS to symphysion, minimum apical border to symphysion. These measurements were taken from Baumgarten et al. (2015).
  - Pubis length deleted
  - Ischium length deleted
- Femur:
  - AP and transverse diameters at midshaft changed to minimum and maximum midshaft diameters
  - Maximum AP length of the lateral condyle added
  - Maximum AP length of the medial condyle added
- Tibia:
  - Maximum distal epiphyseal breadth changed to read "Distal epiphyseal breadth". As defined, this measurement is not a maximum.
  - Nutrient foramen diameters changed to maximum and minimum midshaft diameters
  - Circumference at nutrient foramen changed to circumference at midshaft

Appendix A contains the revised Forensic Recording Forms.

INTRODUCTION

Data Collection Procedures For Forensic Skeletal Material 2.0

INTRODUCTION

The collection of forensic data can be time consuming. This effort must be expended, however, to provide a sound foundation for research in forensic anthropology.

The skeletal observations should be a part of every forensic investigation. With some practice and an assistant, the cranial and postcranial measurements can take less than an hour to complete. The recording form has been compiled and revised by the authors, based in part on the assistance and suggestions of current practitioners in the field. Specific instructions for the completion of each section of the forensic recording sheet are presented in the following pages. A few general guidelines for recording data are discussed below.

- The fastest method involves an observer and a recorder. In preparation for analysis, the available skeletal remains should be arranged in anatomical order. Indicate the side from which the measurement is taken by recording the measurement in the appropriate column. If time is at a premium, it would be better to record all bone measurements from one side rather than both sides on fewer bones. As a standard procedure, the left side measurement is used if only one side is measured. The right side should be substituted for the left when the latter is missing or cannot be observed for other reasons, such as a bone with pathological lesions.

- Measurements should be recorded in millimeters. Round to the nearest mm when a measurement is between two mm markings. For example, a measurement of 41.5 should be recorded as 42. while a reading of 41.4 should be rounded to 41.

- Due to the nature of recovered skeletal material, estimations of measurements are often necessary. Experienced observers can usually provide “reasonable” estimates of osteometric dimensions and correct for minor changes in the bone. For instance, when measuring long bones, older individuals can show a marked degree of arthritic lipping around joint surfaces. These age related changes should not be included in a measurement, and these measurements can be adjusted easily. When a skull is edentulous, however, the position of prosthion can change dramatically, and there are no guidelines for estimation in such cases. We recommend that the observer refrain from making such estimates. When estimates of measurements are made they should be put in parentheses or identified by an asterisk with a footnote. It is difficult to establish a sound standard for qualifying estimates, as such determinations reflect personal bias and experience. If you have serious doubts about any measurement do not write it on the sheet.

- For each relevant postcranial bone, circle the appropriate letter indicating epiphyseal presence (P = present; A =absent) to indicate if measurements are recorded with or without epiphyses (diaphyseal length). This is especially important when measuring sub-adults.

SECTION 1 : EPIPHYSEAL UNION

Epiphyseal Closure

INTRODUCTION

Studies of epiphyseal closure have encountered a variety of problems including inadequate sample sizes, underrepresentation by race and sex, and uncertainty regarding the actual age of the observed specimens. As a result, standards for determining epiphyseal closure exhibit a number of limitations to their application in the aging of skeletal material. Consequently, data collected on epiphyseal union is invaluable to practicing forensic anthropologists. The FDB collects fusion data for 27 epiphyseal regions (Table 1.1).

Three stages are considered for purposes of easily replicable observation of epiphyseal suture closure. Observations are made directly on the bone and each area of closure is scored as falling within one of the three stages of closure.

When recording epiphyseal closure, list the term that most appropriately describes the stage of closure for each observation (Appendix A, Item 38 through 64).

Stages of Epiphyseal Closure:

1- *No Union* (with or without separate epiphyses) -The surface of the metaphysis has grooves and ridges and is granular in appearance (Figure 1.1).

2- *Partial Union* – Part of the epiphysis is attached to the diaphysis of the bone. (Figure 1.1).

3- *Complete Union* - Complete or nearly complete filling in and smoothing over the bone, finely granular in appearance (Figure 1.1).

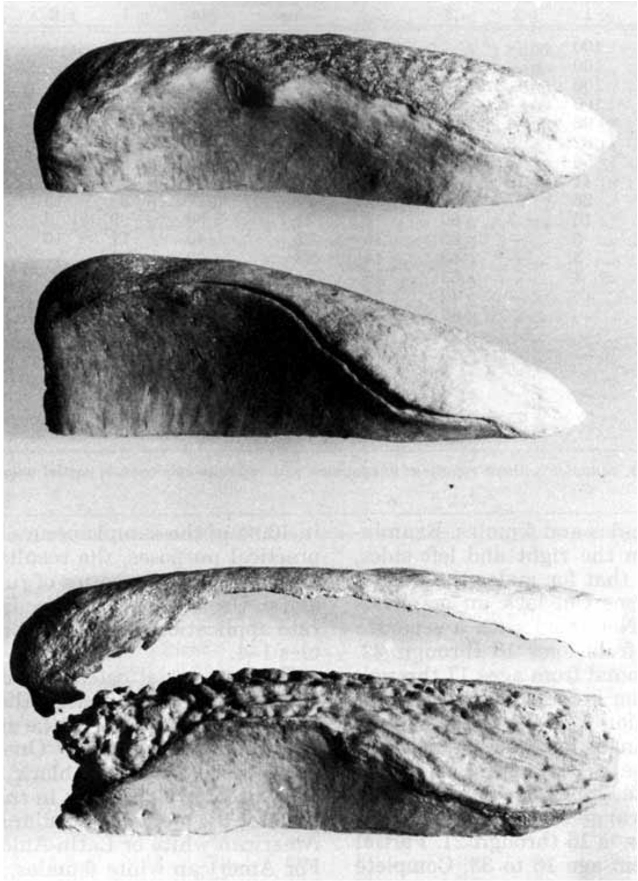

Figure 1.1
Stages of epiphyseal union
(Webb and Suchey 1985:461, Figure 2).

Table 1.1 Selected sites of epiphyseal closure.

38. Basilar Suture	52. Ischial Tuberosity
39. Medial Clavicle	53. Anterior Iliac Crest
40. Atlas-Anterior	54. Proximal Humerus
41. Atlas-Posterior	55. Medial Epic. Hum.
42. Axis-Anterior	56. Proximal Radius
43. Axis-Posterior	57. Distal Radius
44. Cervical Vert. Rim	58. Proximal Ulna
45. Thoracic Vert. Rim	59. Distal Ulna
46. L5 Body-Arch	60. Femur Head
47. Lumbar Vert. Rim	61. Greater Trochanter
48. Sacrum (S1/2)	62. Distal Femur
49. Sacrum (S2/3)	63. Proximal Tibia
50. Sacrum (S3/4)	64. Distal Tibia
51. Innom. Prim. Elem.	

SECTION 2 : TRANSITION ANALYSIS

The Transition Analysis Aging Method

INTRODUCTION

This manual covers how to record three skeletal features used to estimate age in adults with the Transition Analysis method and computer program: the pubic symphysis, iliac portion of the sacroiliac joint, and cranial sutures. Beginning in 1996, we developed new scoring systems for age-progressive changes in bony morphology and examined two known age-at-death skeletal collections (Terry and Coimbra) to estimate the age distribution associated with each of the newly defined stages. The program calculates estimated ages (maximum likelihoods) and confidence intervals for forensic and archaeological skeletons. Further explanation of the procedure can be found in Boldsen et al. (2002), and the results of a validation study in Milner and Boldsen (2012).

Not all skeletal traits have to be present to generate an age estimate. That is, the procedure was designed to accommodate the possibility that only a partial skeleton may be available for observation.

Trait location

It is essential to score only what is happening in the location specified in each of the descriptions, and not be influenced by the appearance of the anatomical unit as a whole. That is because the ages of transition for the young to old stages pertain to specific parts of the anatomical units under consideration. Inattention to adhering to the scoring locations will produce nonsensical results.

Ambiguous skeletal features

On occasion it is difficult or impossible to distinguish between sequential stages in a particular anatomical feature. That can be a result of a pathological process or postmortem erosion. There is still information, however, in such characters, as long as something is visible. You should record whatever is observable, using two or more stage designations, as appropriate (e.g., Stages 3-4). Doing so allows one to take full advantage of the meager information available in the damaged bony structure. For example, a partly observable pubic symphysis ventral margin that has at least 1 cm of rim visible would be scored as *Rim* (6) and *Breakdown* (7). A 6-7 score acknowledges that a rim is present, so an earlier stage is not appropriate. However, there might have been sufficient antemortem marginal erosion to be classified as *Breakdown* (7) in the part of the bone that is missing. The 6-7 designation indicates the individual was at least in Stage 6, and might have been in the next higher one (i.e., Stage 7), but we will never know.

When a particular component cannot be observed it should be coded as unscorable. Usually such situations arise when there is postmortem damage, but can happen as a result of antemortem alterations to normal skeletal structures, as well. For example, large parity pits can eat deeply into the dorsal portion of the pubic symphysis of females, eliminating features that otherwise could be scored.

Figures and Illustrations

Images are from France Casting pubic symphysis casts and bones from several institutions and are coded as follows in the Figures:

- **FC** - France Casting male and female Suchey-Brooks stages (12 each), and male and female instructional casts
- **MC** - Forensic cases from Mercyhurst College
- **T** - Terry Anatomical Collection, Smithsonian Institution
- **B** - William M. Bass Donated Collection, University of Tennessee
- **P** - University of Pretoria
- **NF** - Norris Farms Collection, Illinois State Museum

Each photograph is accompanied by stage name and numerical designation for the stage; the part of interest, if relevant; and specimen designation.

Some images are reversed to make it easier to compare one specimen to the next. For pubis symphysis images, the left side of the image is dorsal, and the right side is ventral. Multiple examples of stages illustrate the variation one might encounter when examining skeletons.

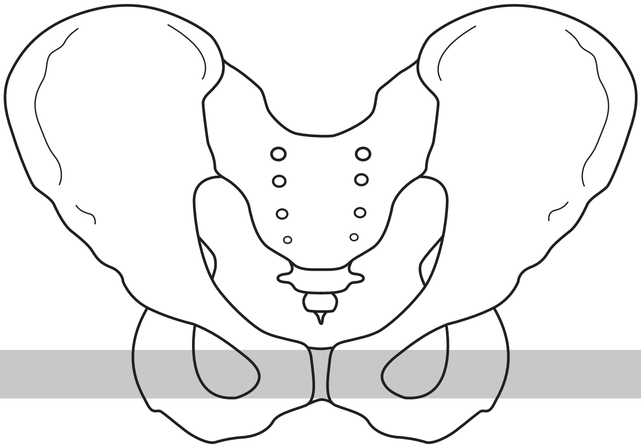

Pubic Symphysis

Five separate components are examined for the pubic symphysis:

- **I. Symphyseal Relief**
- **II. Dorsal Symphyseal Texture**
- **III. Superior Protuberance**
- **IV. Ventral Symphyseal Margin**
- **V. Dorsal Symphyseal Margin**

The various features are based on previous descriptions of bony changes in the pubic bone, especially those of Todd (1920) and McKern and Stewart (1957), supplemented by observations of numerous North American and Danish archaeological skeletons. Many terms used here are derived from this earlier work.

Users of Transition Analysis can gain experience with the changes that take place in the pubic symphysis by closely examining the excellent casts in the McKern-Stewart and Suchey-Brooks pubic bone sets. For sake of convenience, the several parts of the roughly oval symphyseal face are described as superior, inferior, ventral (anterior), and dorsal (posterior), even though such terms are not entirely accurate when the bone is oriented in proper anatomical position.

Opposing pubic bones tend to resemble one another, but they often differ in specific details. Therefore, the left and right sides are scored separately to accommodate that variation.

I. Symphyseal Relief

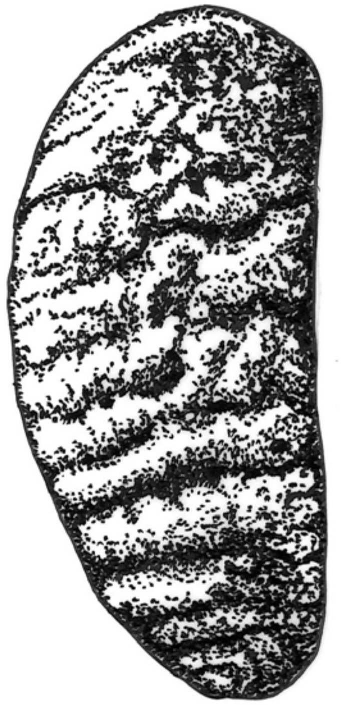

Fig. 2.1 ***Symphyseal Relief***
(Note: dorsal is on the left side, ventral on the right in all pubic symphysis figures)

LOCATION

The terms for this feature generally follow those of McKern and Stewart (1957). Although the entire face should be considered, the billowing is typically most clearly seen in the dorsal half of the symphyseal face (Figure 2.1). In fact, low ridges of bone, the billowing, can be entirely absent from the ventral symphyseal face, beginning as early as the ventral beveling stage.

CHARACTERISTICS [STAGES]

1. Sharp billowing
2. Soft, deep billowing
3. Soft, shallow billowing
4. Residual billowing
5. Flat
6. Irregular

DEFINITIONS

1. *Sharp billowing*: Sharply crested ridges of bone cover at least half of the surface (Figure 2.2). Deep and distinct furrows that extend completely across the symphyseal face separate well-defined ridges. The deepest furrows cut into the ventral and dorsal margins of the symphyseal face, often interrupting the edge of the bone and giving it a jagged appearance. *Sharp Billowing* has only been seen in teenagers and very young adults.

2. *Soft, deep billowing*: Softly crested to low billows separated by deep furrows extend across at least half of the surface (Figure 2.3), typically the dorsal half (demiface). The furrows do not appear as if they have been filled in with bone. The depth between high and low points of adjacent ridges and furrows is 2 mm or more.

3. *Soft, shallow billowing*: Low but clearly visible and discrete billows separated by shallow furrows are present on at least half of the dorsal demiface (Figure 2.4). The remnants of an earlier ridge and furrow system dominate the dorsal demiface, and the furrows look as if they have been filled partially with bone. Billows occupy most or all of the dorsal demiface, and in some individuals they extend anteriorly to reach the ventral margin.

4. *Residual billowing*: Billows are barely visible, being only slightly elevated above the symphyseal face (Figure 2.5). They tend to blend into one another to form low and indistinct raised areas that lack clearly defined furrows between them. The slightly raised areas occupy only part of the symphyseal face, typically about one-third of the middle to inferior portions of the dorsal demiface. Individual billows usually cross only part of the symphyseal face, typically less than one-half its width, being concentrated on the dorsal demiface. There must be two or more adjacent raised areas corresponding to billows to qualify as *Residual Billowing*. A single isolated bony elevation is not sufficient to be classified as *Residual Billowing*; instead, such specimens are considered *Flat* (see Stage 5 below).

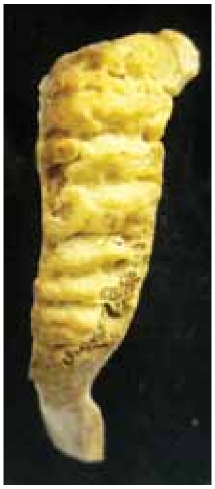

Figure 2.2. ***Sharp billowing of the symphyseal surface*** *[1] (AZ 175).*

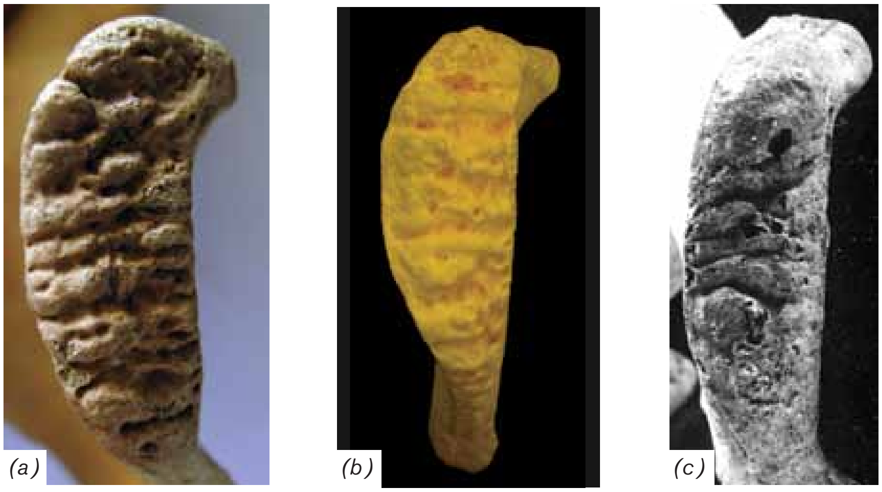

Figure 2.3. ***(a) Soft, deep billowing*** *[2] (P 6192, image reversed),* ***(b) Soft, deep billowing*** *[2] (FC F-I-1), and* ***(c) Soft, deep billowing*** *[2] (T 222R, image reversed).*

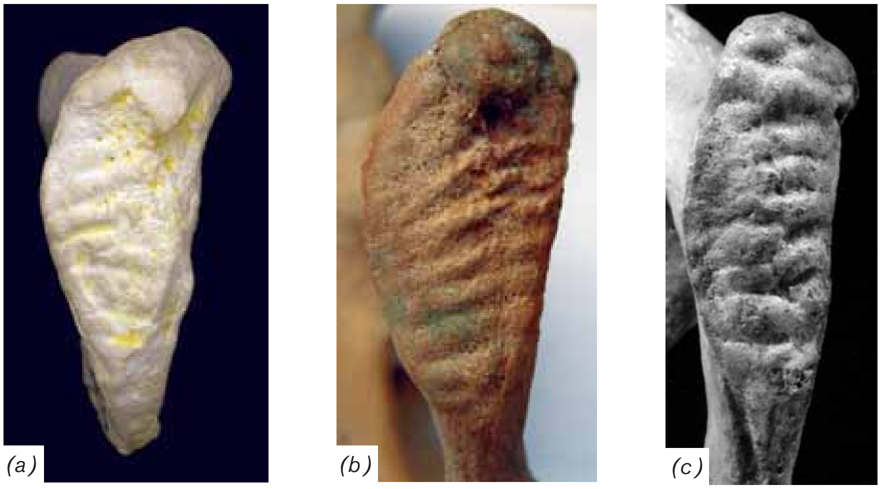

Figure 2.4. ***(a) Soft, shallow billowing*** *[3] (FC M-III-1),* ***(b) Soft, shallow billowing*** *[3] (P 5152, image reversed), and* ***(c) Soft, shallow billowing*** *[3], (T 385, image reversed).*

5. *Flat*: More than one-half of the symphyseal face within well-defined margins is flat or slightly recessed, and is often surrounded by a well-developed rim (Figure 2.6). Occasionally small, low pillows of bone give the otherwise flat surface a slightly bumpy appearance, but the symphyseal face does not conform to *Residual Billowing* (i.e., there is no more than one discrete and well-defined low raised area corresponding to what had been a more extensive series of billows). If there is a gap where the ventral rampart has failed to extend along the entire ventral edge of the pubis (a ventral hiatus), the surface within the gap does not receive a score.

6. *Irregular*: Pitting, which can be deep, covers more than one-half of the symphyseal face, giving it an irregular and disfigured appearance (Figure 2.7). The pits can be accompanied by small, sharp exostoses scattered across the face. Occasionally, in older individuals an otherwise flat face is thickly covered by low but typically sharp exostoses that give the symphyseal surface a markedly irregular appearance. Pitting in such specimens might be minor, but the bone is still classified as Irregular. Similar to the *Flat* category, the scored part of the symphyseal face does not include the ventral gap, if present. In Irregular specimens, the margins of the symphyseal face are typically defined by the *Rim* and *Breakdown* stages of the **Ventral and Dorsal Margin** components.

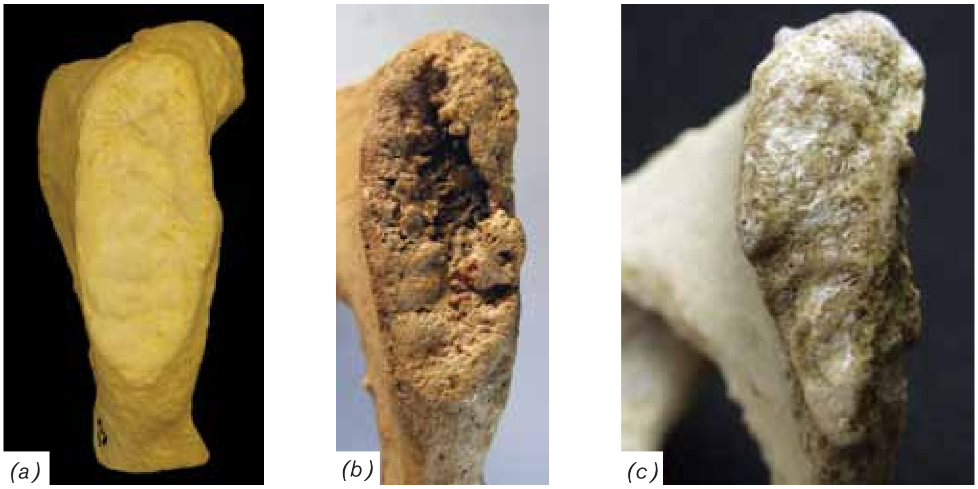

Figure 2.5. ***(a) Residual billowing*** *[4], inferior part (FC M-13),* ***(b) Residual billowing*** *[4], inferior part (P 6120, image reversed), and* ***(c) Residual billowing*** *[4], inferior part (MC 7-76).*

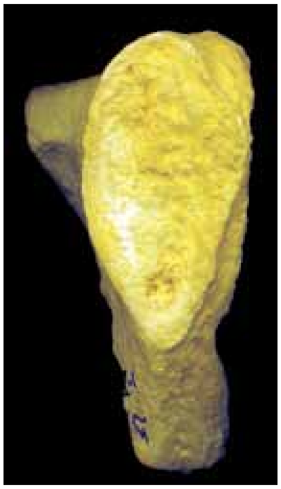

Figure 2.6. ***Flat*** *[5] (FC M-V-1).*

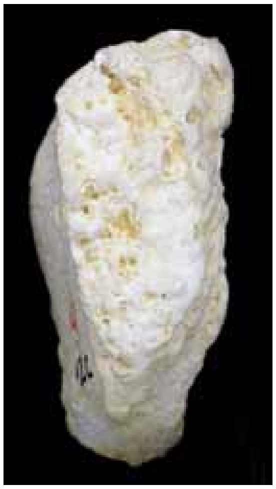

Figure 2.7. ***Irregular*** *[6] (FC F-12C).*

II. Dorsal Symphyseal Texture

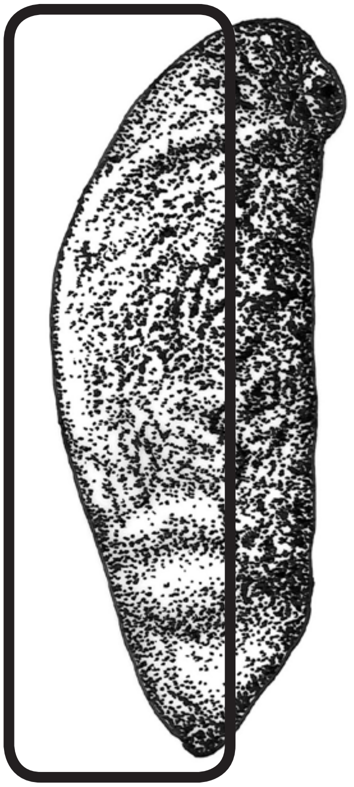

Figure 2.8. ***Dorsal Symphyseal Texture***

LOCATION

The dorsal portion of the surface is examined (Figure 2.8).

CHARACTERISTICS [STAGES]

1. Smooth (fine-grained)
2. Coarse-grained
3. Microporosity
4. Macroporosity

DEFINITIONS

1. *Smooth (fine-grained)*: Smooth to fine-grained bone extends across most, or all, of the dorsal demiface (Figure 2.9).
2. *Coarse-grained*: Coarse-textured bone covers over one-third of the dorsal demiface (Figure 2.10). The surface looks like packed fine sand, similar to medium-grained sandpaper. Americans may recognize it best as the surface of a sugar cube.
3. *Microporosity*: Porous bone covers over one-third of the dorsal demiface. It looks as if the surface was pierced by closely packed pinpricks (Figure 2.11).
4. *Macroporosity*: Deep pits cover over one-third of the dorsal demiface, giving it an irregular appearance (Figure 2.12). The pits are at least 0.5 mm in diameter, and generally are spaced close together. Sometimes the symphyseal surface is so irregular from pitting that it resembles the edge of a sponge. The surface looks like it was pierced by closely packed pinheads (the bulbous end of the same shirt or blouse pin in the previous description).

SCORING TIPS

In most instances it is difficult to identify the proper stage from photographs. So if the actual specimen is unavailable, it is generally best to score this feature as not observable.

The ventral part of the bone, when ventral beveling is present, is often pitted, giving it the appearance of microporosity. A porous ventral demiface should not be confused with what is happening in the dorsal demiface.

It is our impression that prehistoric Native American skeletons have microporosity more often and at an earlier age than medieval Scandinavians. More to the point, they differ from the modern Terry and Coimbra collection skeletons used to generate transition curves used when estimating age. Therefore, it is best not to rely heavily on this feature of the symphyseal face when examining Native American skeletons. In fact, it is prudent to record **Symphyseal Texture** stages for Native Americans, but treat them as missing data when estimating age with the computer program (i.e., do not enter the scores on the data entry page).

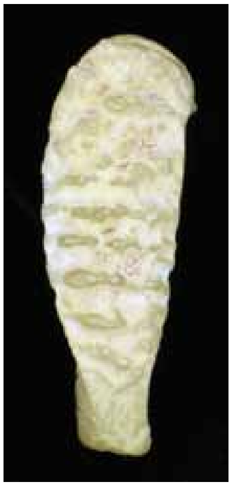

Figure 2.9. **Smooth** [1] (FC M-I-1).

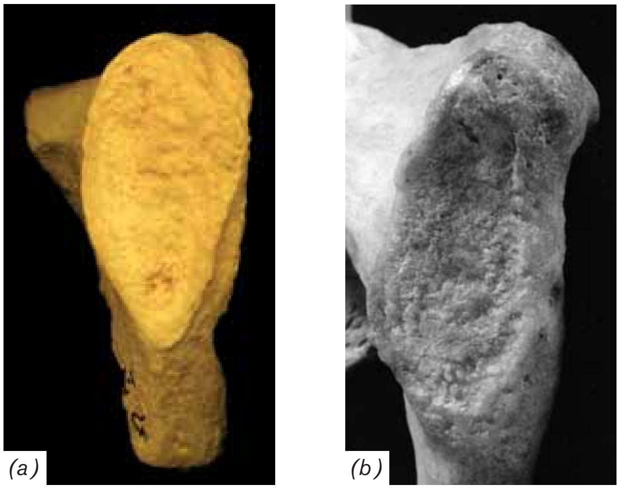

Figure 2.10. **(a) Coarse grained** [2] (FC M-V-1) and **(b) Coarse grained** [2] (T 862).

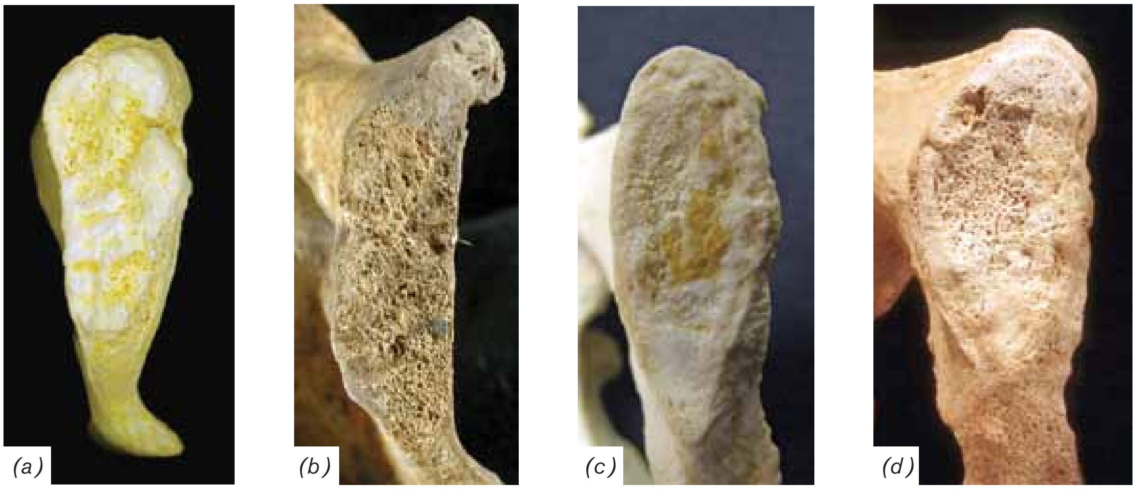

Figure 2.11.
(a) Microporosity [3] (FC F-III-1), **(b) Microporosity** [3] (MC 6-69), **(c) Microporosity** [3] (MC 7-55, image reversed), and **(d) Microporosity** [3] (NF 19).

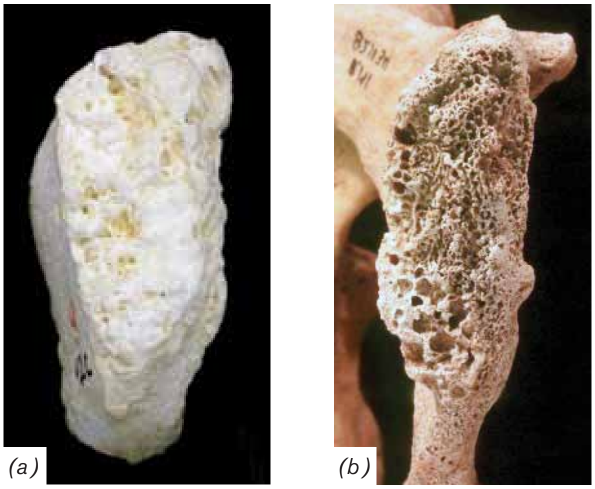

Figure 2.12.
(a) Macroporosity [4] (FC F-12C) and **(b) Macroporosity** [4] (NF 191).

III. Superior Protuberance

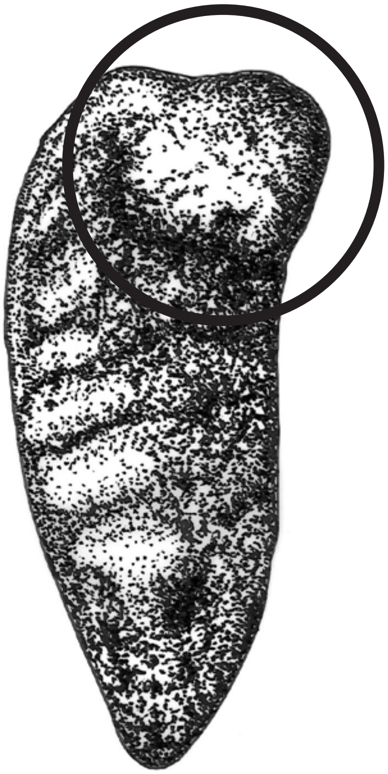

Figure 2.13. Superior Protuberance

LOCATION

The superior part of the symphyseal face is examined for a distinct knob of bone or, later, an elevated area (Figure 2.13).

CHARACTERISTICS [STAGES]

1. **No protuberance**
2. **Early protuberance**
3. **Late protuberance**
4. **Integrated**

DEFINITIONS

1. *No protuberance*: Deep to shallow billowing is present in the superior part of the symphyseal face (Figure 2.14). There are no signs of a bony protuberance. In young individuals, this part of the symphyseal face can be poorly differentiated from the non-articular portion of the pubis immediately lateral to the joint. This stage is on a symphyseal face characterized by a youthful ridge-and-valley surface.

2. *Early protuberance*: A distinct bony knob of variable dimensions with well-defined margins is visible in the superior part of the symphyseal face (Figure 2.15). It projects above the plane(s) defined by the immediately adjacent symphyseal face (i.e., the superior portions of the dorsal and ventral demifaces, where the latter can be characterized by ventral beveling). The surface of the bony protuberance is typically smooth to fine grained. In many specimens, the bony knob looks like a split pea stuck on the bone.

3. *Late protuberance*: The superior part of the symphyseal face is raised somewhat above the rest of the articulation surface (Figure 2.16). The elevated area is typically located mostly on the ventral side of the midline. The margins of the slightly raised area are poorly defined. These raised areas tend to look like a continuation of a relatively smooth symphyseal surface, except they slope slightly upward to meet the superior margin of the bone. Thus the *Late Protuberance* is more completely integrated with the rest of the symphyseal face than the distinctly knob-like *Early Protuberance*. *Late Protuberance* should not be confused with a narrow raised marginal rim that can border the cranial end of the symphyseal face in many specimens, especially females with narrow pubic symphyses. For a *Late Protuberance* to be scored as present, the slightly raised area must extend onto the symphyseal face; that is, it is not restricted to the rim alone. Occasionally, the superior part of the symphyseal face can be partly separated from the rest of the face by marked and extensive pitting of the middle symphyseal surface. Care must be taken not to confuse an isolated segment of the symphyseal face with a *Late Protuberance* stage. It is only *Late Protuberance* if the superior end of the symphyseal surface is elevated above the portions of the face not affected by the pitting.

4. *Integrated*: The symphyseal face's superior end displays no signs of a low bony elevation (Figure 2.17). The area where the protuberance was formerly present is fully integrated

with the rest of the symphyseal face. That is, the smooth to irregular (usually pitted) symphyseal face is essentially flat. This stage, the absence of a raised area, is distinguishable from Stage 1, *No Protuberance*, because the superior portion of the symphyseal face is flat, not the ridge-and-valley surface typical of the initial *No Protuberance* stage. The *Integrated* stage also frequently has a narrow elevated rim demarcating the superior symphyseal surface, which is a continuation of the ventral or dorsal rims.

SCORING TIPS

Do not confuse a narrow rim bordering the superior margin of the symphyseal face, which commonly occurs in the terminal *Integrated* stage, with the earlier *Late Protuberance* stage. The two stages are distinguishable because the *Late Protuberance* raised area extends onto the upper part of the symphyseal face. In the *Integrated* stage, a flat symphyseal face extends up to, and abuts, a narrow rim, which is commonly present.

Occasionally, a gap exists in the superior one-half of the ventral margin, but the ventral rampart is otherwise completely formed. In that case, the presence of a protuberance is often not scored as the appearance of the bone can be confusing.

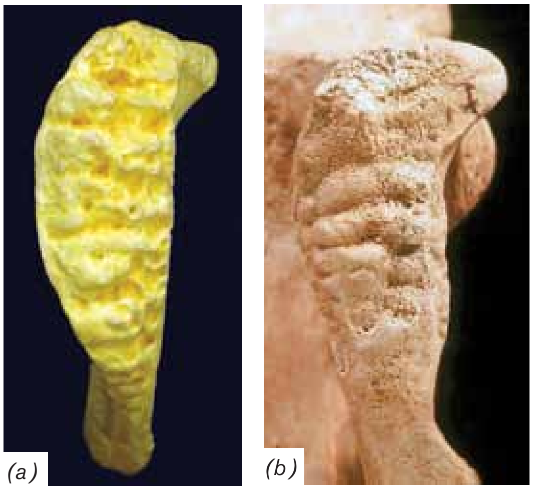

Figure 2.14. **(a) No protuberance** [1] (FC F-I-1) and **(b) No protuberance** [1] (NF 44).

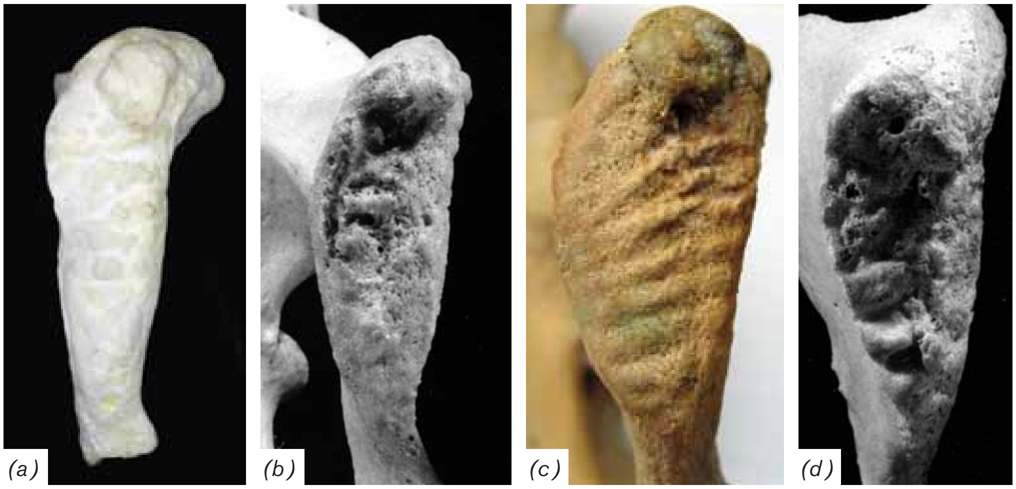

Figure 2.15. **(a) Early protuberance** [2] (FC M-I-2), **(b) Early protuberance** [2] (T 465), **(c) Early protuberance** [2] (P 5152, image reversed), and **(d) Early protuberance** [2] (T 255, image reversed).

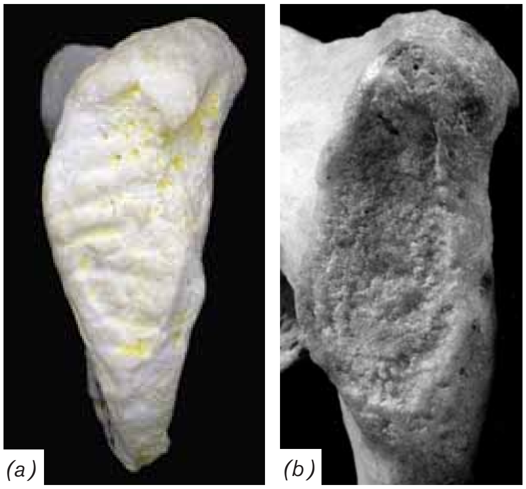

Figure 2.16. **(a) Late protuberance** [3] (FC M-III-1) and **(b) Late protuberance** [3] (T 862).

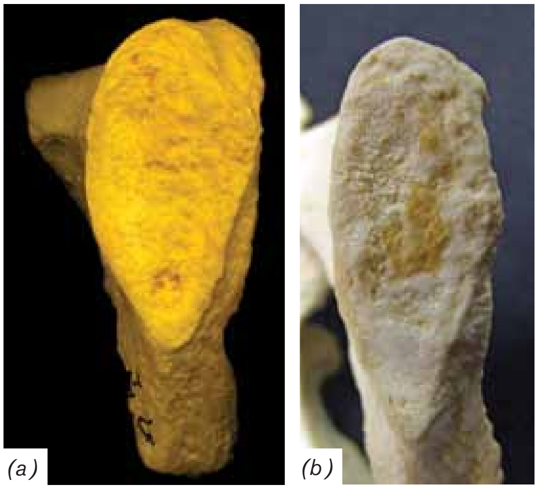

Figure 2.17. **(a) Integrated** [4](FC M-V-1) and **(b) Integrated** [4] (MC 7-55).

IV. Ventral Symphyseal Margin

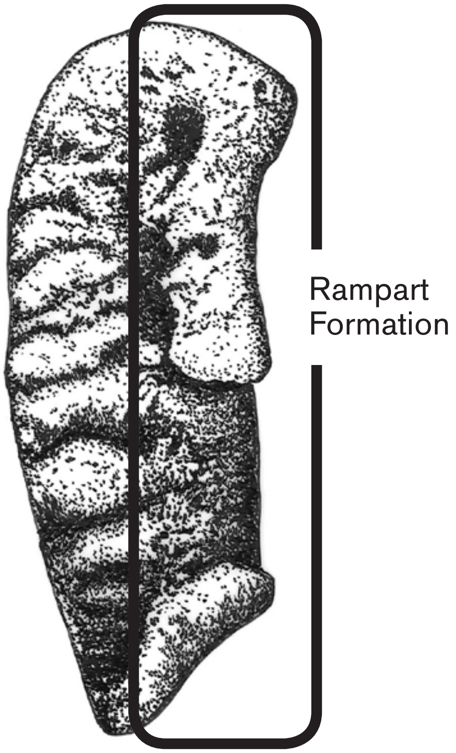

Figure 2.18. ***Ventral Symphyseal Margin***

LOCATION

The ventral margin of the pubic symphysis is scored separately from the rest of the bone (Figure 2.18).

CHARACTERISTICS [STAGES]

1. Serrated
2. Beveled
3. Rampart formation
4. Rampart completion with anterior sulcus
5. Rampart completion without sulcus
6. Rim
7. Breakdown

DEFINITIONS

1. *Serrated*: Ridges and furrows typical of *Sharp* or *Soft Deep Billowing* extend uninterrupted across the ventral part of the symphyseal face, producing a serrated or jagged ventral margin, especially when viewed from the front of the bone (Figure 2.19).

2. *Beveled*: Billows are flattened in the ventral half of the symphyseal face, a process that generally starts at the superior end (Figure 2.20). Flattening, or beveling, must extend along at least one-third of the ventral margin to be scored as present. There is often a reasonably well-defined margin where the beveled ventral portion meets the dorsal demiface immediately posterior to it.

3. *Rampart incomplete*: The ventral rampart, following McKern and Stewart (1957), refers to a distinct outgrowth of bone that ultimately forms the ventral aspect of the symphyseal face (Figure 2.21). The rampart extends from one or both ends of the symphysis, and it often resembles a roll of well-chewed gum stuck on the ventral edge of the symphyseal face. The rampart does not extend along the entire ventral edge, and often some elements of a youthful symphyseal surface can be followed uninterrupted to the ventral edge of the smphysis. In the superior part of the ventral margin, the rampart forms on the *Beveled* surface. In the inferior part of the margin, remnants of the original irregular surface can often be seen dipping below a partially formed rampart, which looks as if it was lying on a shallowly furrowed surface. An incomplete rampart frequently extends inferiorly from the bony protuberance defining the cranial end of the face, sometimes forming a bony elevation that resembles a comma, with the rampart being the tail.[1] A rampart can also extend superiorly from the inferior end of the symphysis. Bony extensions from the superior and inferior ends of the symphysis, if both are present, typically leave a gap in the middle one-third of the ventral margin. An early *Rampart Incomplete* stage can consist of one or more bony knobs, commonly located in the middle one-third of the ventral margin. The knobs can occur with, or without, the formation of a bony rampart extending from the superior and inferior ends of the symphysis. If the rampart is more than two-thirds complete but there is a gap in the superior part of it, you should consider the possibility

[1] A well-developed bony protuberance at the cranial end of the face that lacks a distinct inferiorly projecting ventral rampart should not be coded as Ventral Rampart Formation; that is, the mere existence of a cranially located bony knob without bone being laid down along the ventral margin is not sufficient to score the ventral rampart as present.

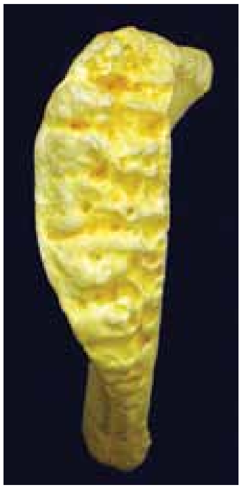

Figure 2.19. ***Serrated*** *[1] (FC F-I-1).*

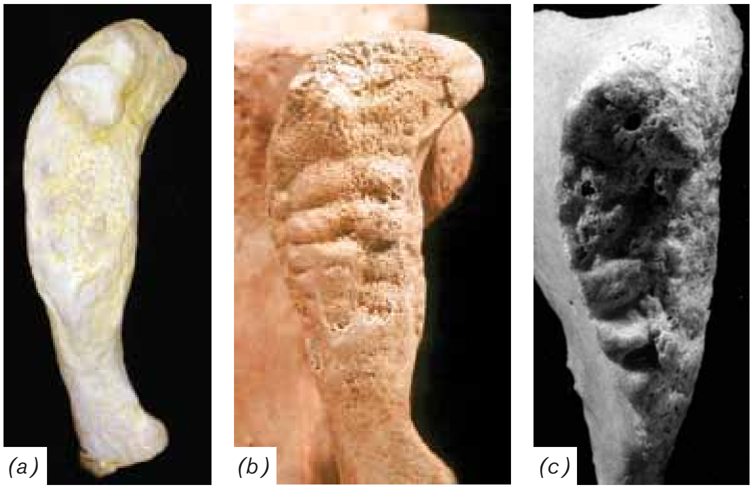

Figure 2.20. ***(a) Beveling*** *[2] (FC M-12),* ***(b) Beveling*** *[2] (NF 44), and* ***(c) Beveling*** *[2] (T 255, image reversed).*

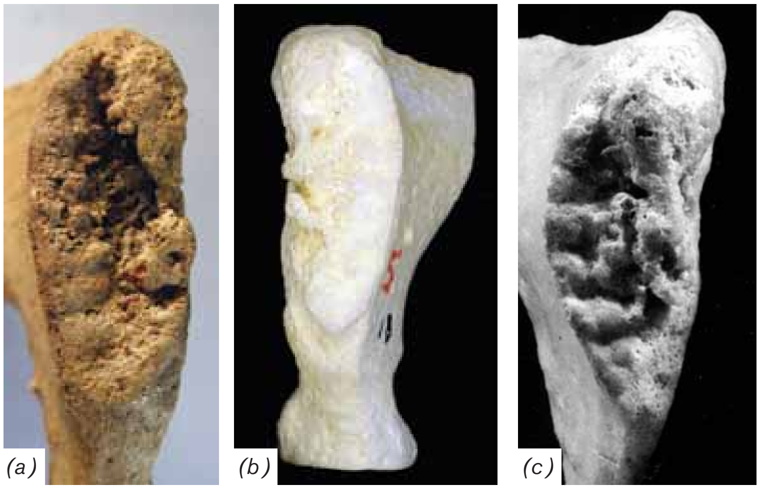

Figure 2.21. ***(a) Rampart formation*** *[3] (P 6120, image reversed),* ***(b) Rampart formation*** *[3] (FC M-1B, image reversed), and* ***(c) Rampart formation*** *[3] (T 255).*

that the specimen is in the *Rampart Complete with Anterior Sulcus* or *Rampart Complete without Sulcus* stages. Occasionally a rampart never completely forms along the ventral margin.

4. *Rampart Complete with Anterior Sulcus*: *Rampart Complete 4 and 5* are similar to one another. What distinguishes them is the appearance of the anterior surface of the bone immediately lateral to the symphyseal margin. In *Rampart Complete with Anterior Sulcus* [4], the ventral rampart is complete, but there is a shallow sulcus extending along much of the length of the ventral pubis immediately lateral to the symphysis (often more pronounced inferiorly). The groove is a residual feature related to rampart formation along the ventral margin. A reasonably flat symphyseal surface extends uninterrupted from the dorsal to ventral margins, so the face is unlike the somewhat furrowed appearance of many *Rampart Incomplete* specimens where there is a shallow groove just dorsal to an incomplete ventral rampart. Occasionally a gap exists in the ventral margin, usually in its superior half; the ventral rampart is otherwise completely formed. This stage is only occasionally found in most skeletal samples that have been examined. Typically the completed rampart is a *Stage 5, Rampart Complete without Sulcus*. The only difference between Stages 4 and 5 is the presence of the anterior sulcus in Stage 4.

5. *Rampart Complete without Sulcus*: The ventral rampart is

complete, and there is no shallow sulcus as described in *Rampart Complete 4*. A reasonably flat symphyseal surface extends uninterrupted from its dorsal to ventral margins, so the face is unlike the somewhat furrowed appearance of many *Rampart Incomplete* specimens where there is also a shallow groove just dorsal to the incomplete ventral rampart. Occasionally there is a gap in the superior half of the ventral margin, but the ventral rampart is otherwise complete (Figure 2.27a). These specimens should be classified as *Rampart Complete*. With regard to *Rampart Complete With and Without Anterior Sulcus (Stages 4 and 5)*, most specimens are in the later Stage 5.

6. *Rim*: A narrow, bony rim defining the ventral margin of the symphysis, perched on top of the ventral rampart, demarcates a usually flat or irregular face (Figure 2.22). The rim does not have to be complete, but it must be at least 1 cm long and readily visible as a raised ridge adjacent to a slightly recessed symphyseal face. The rim can be either a continuous ridge of bone or several segments, as long as 1 cm of an elevated border is present. The rim's crest can be low and rounded, or narrow and sharp. A ventral rim is always formed on top of a ventral rampart. Odd rim-like bone formations on gaps in a rampart or formed with no rampart at all are not scored as a ventral rim.
7. *Breakdown*: The ventral margin of the symphyseal face has begun to break down, as indicated by pitting and an erosion of the *Rim* (Figure 2.23). The breakdown of the ventral margin must exceed 1 cm (either in one spot, or when two or more areas of erosion are combined) to be scored as present. Care must be taken to distinguish antemortem degeneration – that is, true *Breakdown* – from postmortem damage. The latter, of course, can render the bone unscorable if it is extensive enough.

SCORING TIPS

In mature pubic symphyses there can be a gap in the ventral margin, as noted by McKern and Stewart (1957: 77, Fig. 40) who called it the "ventral hiatus (Figure 2.27a)". Gaps, when present, usually occur in the superior half of the pubic symphysis. In most instances, they are readily distinguishable from incomplete rampart formation because the remainder of the ventral rampart appears complete. That is, the ventral margin elsewhere has a rounded to angular edge, an anterior sulcus is typically absent, and a rim might have developed on the part of the rampart that is present. In addition, the symphyseal surface extends uninterrupted from the dorsal to ventral margins, and it is often flat. The appearance of the symphyseal face contrasts sharply with what is present in the typical *Rampart Incomplete* stage. In the earlier *Rampart Incomplete 4* stage, a shallow depression is often present immediately dorsal to the newly formed and still rather narrow rampart, and much of the rest of the symphyseal face is marked by remnants of the original ridges and furrows.

Occasionally in the *Rim* or *Breakdown* stages, there is also a separate bony growth or collar, presumably ossified ligaments, adjacent to the ventral margin that is separated from the symphyseal face by a groove. It only occurs on old people, and it is more commonly found toward the superior end of the pubic symphysis. This bony structure is not scored and should not to be confused with what is happening on the symphyseal surface or its margin.

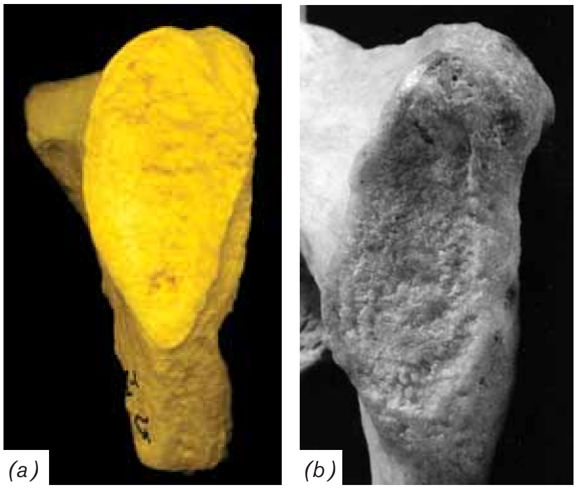

Figure 2.22. ***(a) Rim*** *[6] (FC M-V-1) and* ***(b) Rim*** *[6] (T 862).*

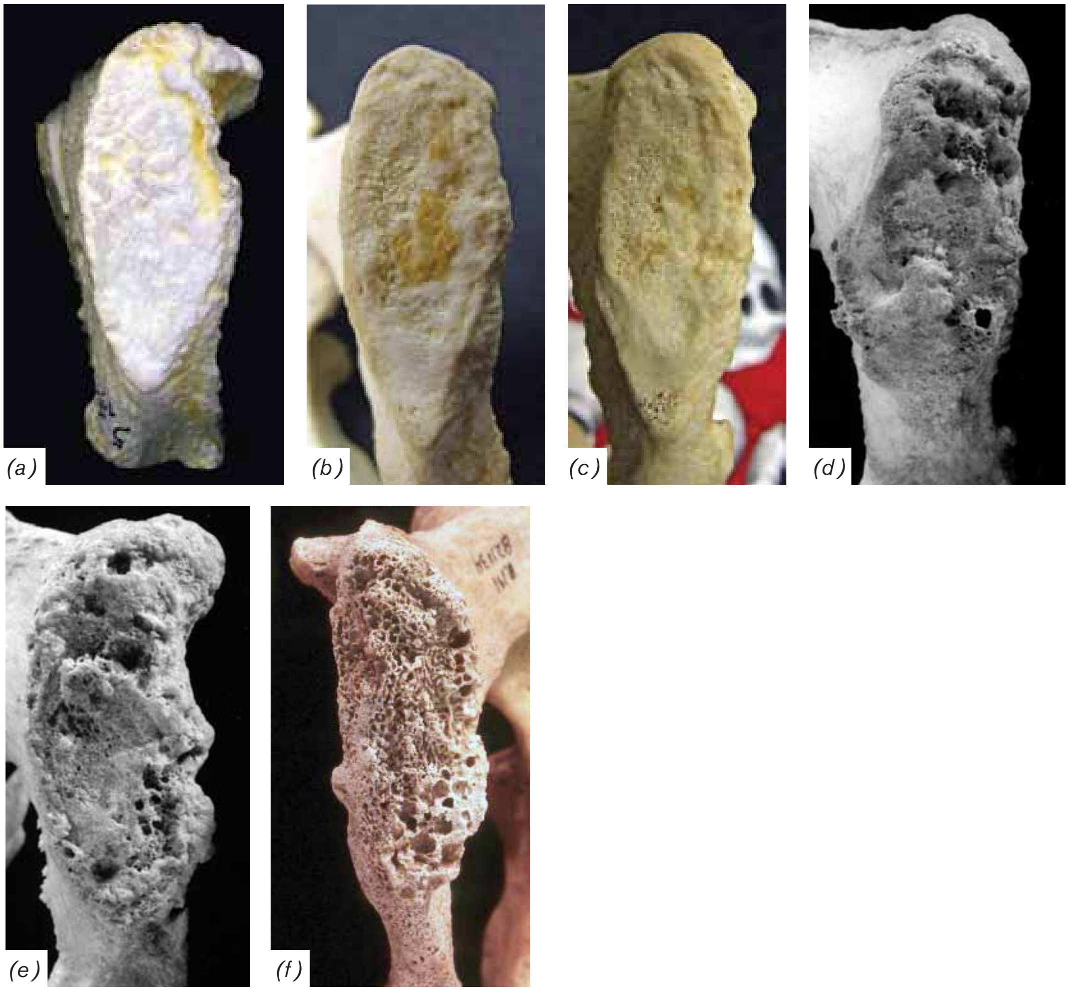

Figure 2.23. ***(a) Breakdown*** *[7] (FC M-VI-2),* ***(b) Breakdown*** *[7], breakdown superiorly and rim inferiorly (MC 7-55),* ***(c) Breakdown*** *[7], breakdown most of margin (MC 7-55, image reversed),* ***(d) Breakdown*** *[7] (T 500),* ***(e) Breakdown*** *[7] (T 1115), and* ***(f) Breakdown*** *[7] (NF 191).*

V. Dorsal Symphyseal Margin

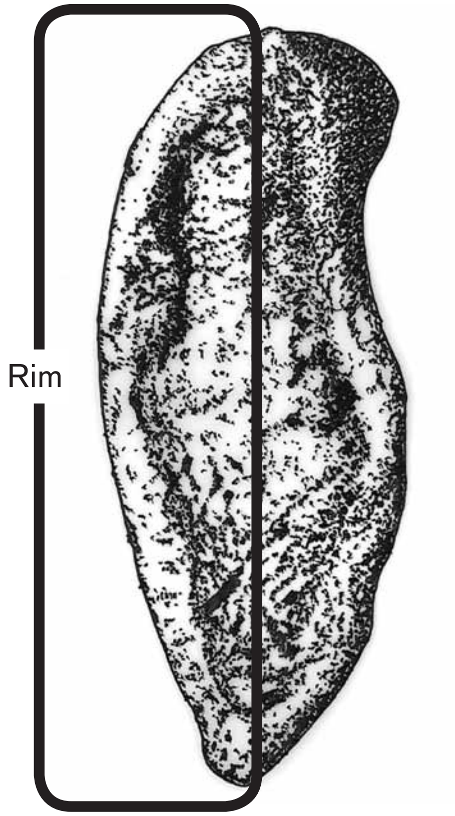

Figure 2.24. ***Dorsal Symphyseal Margin***

LOCATION

The dorsal part of the pubic symphysis is scored separately from the ventral margin (Figure 2.24). In females, dorsally located characteristics can be partly or entirely obscured by large postpartum, or parity, pits. Occasionally, such specimens cannot be scored properly.

CHARACTERISTICS [STAGES]

1. Serrated
2. Flattening incomplete
3. Flattening complete
4. Rim
5. Breakdown

DEFINITIONS

1. *Serrated*: The dorsal margin of the symphyseal face is irregular because ridges and furrows typical of pronounced billowing extend uninterrupted to the edge of the bone (Figure 2.25).

2. *Flattening incomplete*: A well-defined flattened area at least 1 cm long is present where the symphyseal face meets the dorsal margin (Figure 2.26). Flattening usually starts in the superior part of the dorsal demiface; the remainder of the margin has the ridge and valley configuration typical of a youthful symphyseal face. The undulating edge usually occurs in the inferior part of the symphyseal face. Note that billowing is also present on the dorsal demiface, and it typically produces an undulating edge to the pubic symphysis, although it is usually not as extreme as what is found in *Serrated* specimens.

3. *Flattening complete*: There is a rather obvious area of flattening that completely (or almost entirely) covers the symphyseal face where it meets the dorsal margin (Figure 2.27). This flattening seemingly occurs partly through a coalescence of billows. A small area at the inferior end of the dorsal margin occasionally retains an undulating appearance.

4. *Rim*: An elevated bony rim demarcates a flat or, infrequently, an irregular face (Figure 2.28). The rim projects slightly above the symphyseal face, and its crest can be blunt or sharp. The rim does not have to extend along the entire dorsal margin to be scored as present, but it must be at least 1 cm long. The 1 cm rule pertains to either a continuous rim or discontinuous segments that together sum to that length. A rim typically develops first along the superior part of the dorsal margin. It can, however, occur anywhere along the dorsal margin.

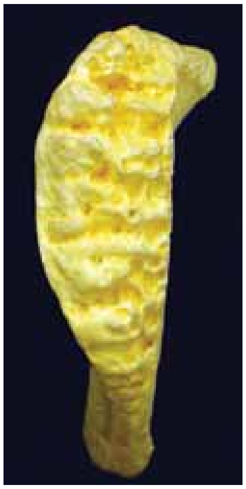

Figure 2.25.
***Serrated** [1] (FC F-I-1).*

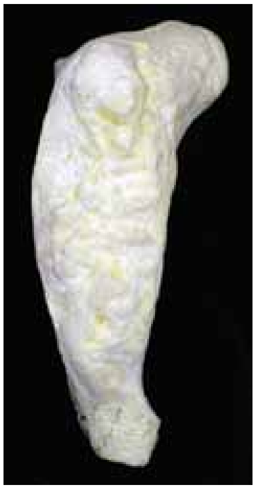

Figure 2.26.
***Flattening incomplete** [2] (FC M-II-1).*

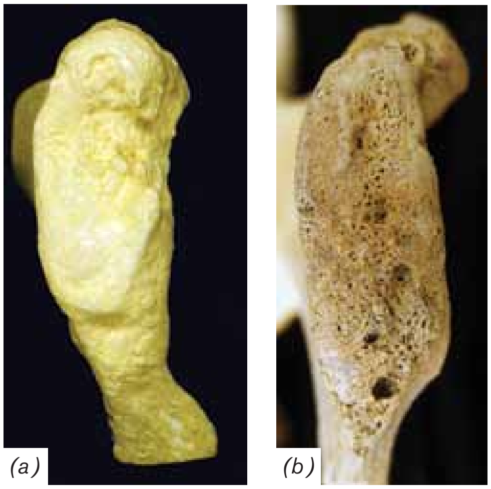

Figure 2.27.
***(a) Flattening complete** [3] (FC M-III-2) and*
***(b) Flattening complete** [3] (NF 229).*

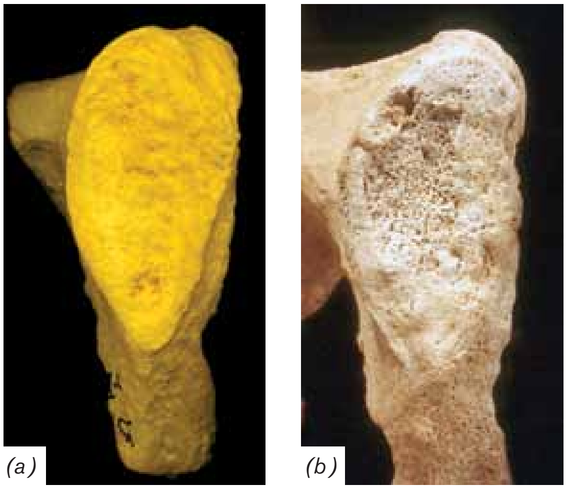

Figure 2.28.
***(a) Rim** [4] (FC M-V-1) and **(b) Rim** [4] (NF 19).*

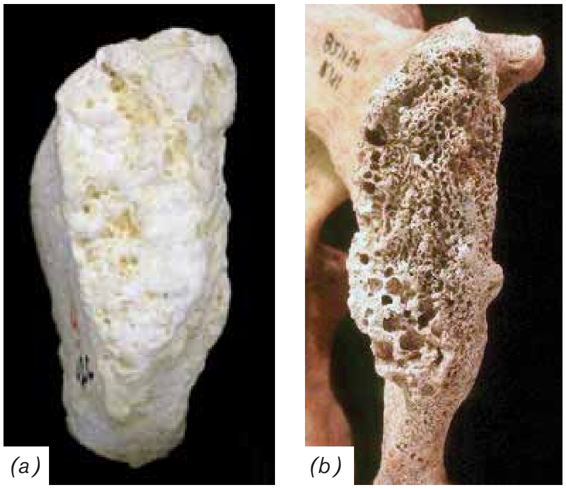

Figure 2.29.
***(a) Breakdown** [5] (FC F-12C) and*
***(b) Breakdown** [5] (NF 191)*

5. *Breakdown*: The dorsal margin where the Rim is located shows evidence of breakdown, specifically a pitting and erosion of the edge of the pubic symphysis (Figure 2.29). The breakdown must exceed 1 cm in length either in one spot or when two or more areas of erosion are combined. Care must be taken to differentiate antemortem degeneration of the margin from postmortem damage, which is of no concern. Antemortem destruction attributable to large parity pits in females that can undercut the dorsal margin is not considered breakdown in the sense of the term as used here. It might not be possible to score those specimens; when that occurs, the component is simply missing data.

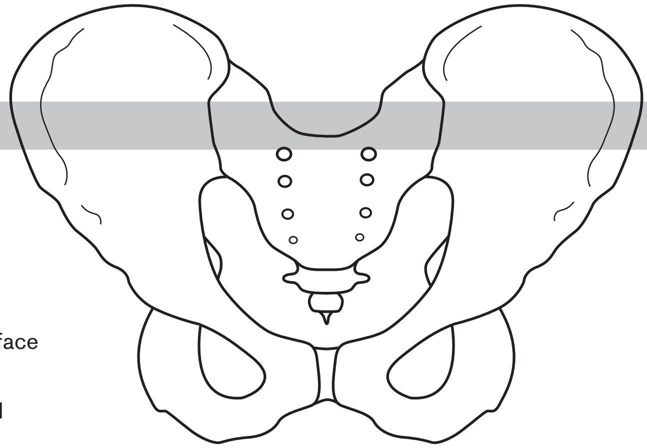

Sacroiliac Joint

Two aspects of the sacroiliac joint on the ilium are examined (Figure 2.30):

1. the auricular (joint) surface
2. the ilium immediately posterior to the joint surface

Anatomical features and terms follow those of Lovejoy and colleagues (1985) where possible.

Nine separate components are examined for the iliac portion of the sacroiliac joint:

- **I. Superior Demiface Topography**
- **II. Inferior Demiface Topography**
- **III. Superior Surface Characteristics**
- **IV. Middle Surface Characteristics**
- **V. Inferior Surface Characteristics**
- **VI. Inferior Surface Texture**
- **VII. Superior Posterior Iliac Exostoses**
- **VIII. Inferior Posterior Iliac Exostoses**
- **IX. Posterior Exostoses**

I. Superior Demiface Topography

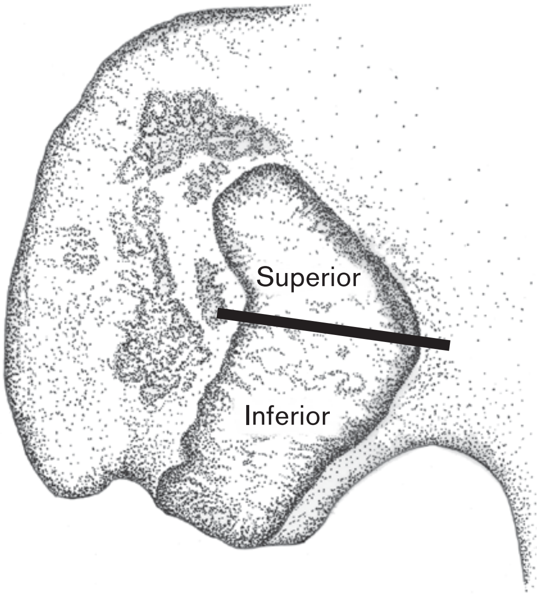

Figure 2.30 ***Superior Demiface Topography***

LOCATION

The superior demiface is examined. The two demifaces (superior and inferior) are divided by a line extending posteriorly from the most anterior point of the apex to the posterior joint margin.

CHARACTERISTICS [STAGES]

1. Undulating
2. Median elevation
3. Flat to Irregular

DEFINITIONS

1. *Undulating*: The surface is undulating in a superior to inferior direction. There is no centrally located and linear area of elevated bone (*Median Elevation*). When the entire articular surface is viewed in aggregate, the overall effect is of two or three low waves proceeding lengthwise along the joint (Figure 2.31).

2. *Median elevation*: In the middle to posterior part of the demiface there is a broad raised area where the joint surface is elevated slightly above the rest of the joint (Figure 2.32). The elevation is flanked anteriorly, posteriorly, or both by one or two long, low areas. The elevated area takes the form of an elongated ridge with the long axis paralleling the main orientation of the demiface. It is not unusual for the elevated area to be restricted to a noticeably raised area that does not extend the entire length of the demiface. When that occurs, the raised area is typically located in the inferior portion of the demiface. To be scored as present, the elevated area must extend along at least one-third of the joint surface's length.

3. *Flat to irregular*: The surface is essentially flat or recessed, a result of marginal lipping, or it is irregular from degeneration of the joint or the formation of low, pillow-like exostoses (Figure 2.33).

SCORING TIPS

These comments pertain to **Superior and Inferior Demiface Topography**. The first thing to do is to place your thumb such that it masks the superior demiface, and then score the inferior part of the joint. The characteristics of the inferior demiface are generally easier to see, and to score reliably, than the superior demiface, especially with regard to the *Median Elevation*. Then move your thumb so it masks much of the inferior part of the joint so you just look at the superior part. The purpose of this exercise is to prevent being influenced by what is happening in the part of the joint that is not being scored.

When deciding which of the stages is represented, it is easiest to determine if a *Median Elevation* is present first. It will be generally more pronounced on the inferior demiface. If it is not present, the surface is typically in the *Flat* to *Irregular* stage. The *Undulating* variant is almost always found on the ilia of young people in their teens or twenties. The **Superior Demiface** *Undulating* and *Median Elevation* features are more subdued and harder to distinguish than those of the **Inferior Demiface**.

Most specimens are either *Median Elevation* [2] or *Flat to Irregular* [3]. Occasionally in young adults a median elevation appears to be in the process of forming on an otherwise undulating surface. The median elevation is short and typically centrally located on the superior demiface (rarely this occurs on the inferior demiface). Because the median elevation, if present, is poorly defined, these specimens are best coded as 1-2.

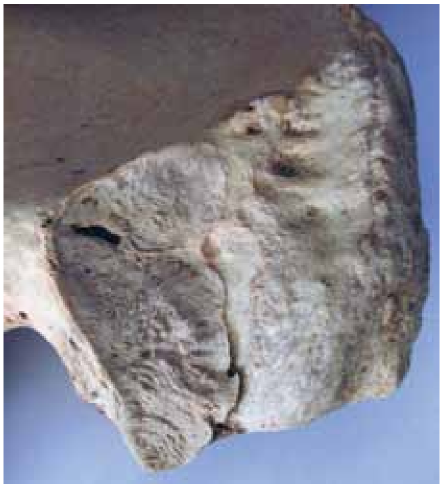

Figure 2.31. ***Undulating*** *[1], inferior and superior (subtle) parts (P 5897).*

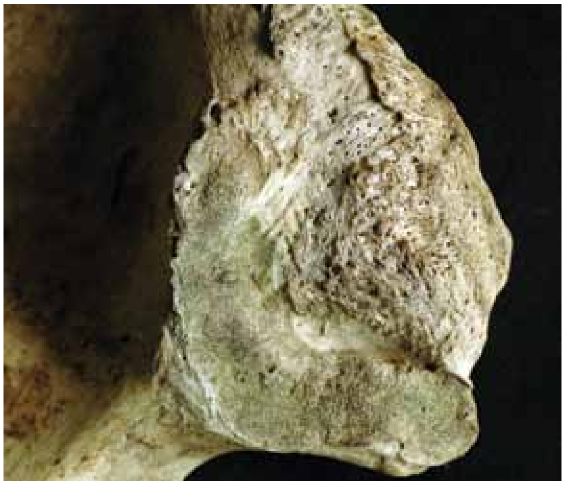

Figure 2.32. ***Median elevation*** *[2], superior part (MC 6-73).*

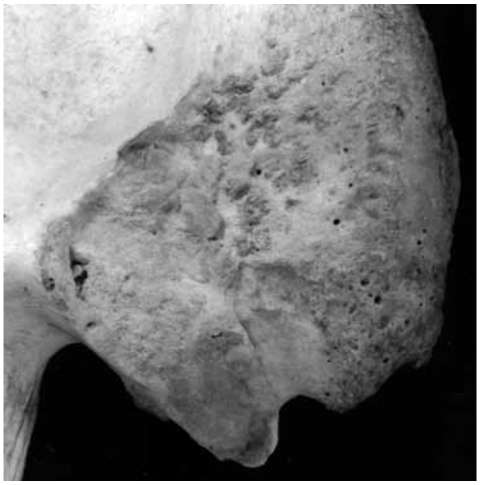

Figure 2.33. ***Flat*** *[3], superior and inferior parts (T 500).*

II. Inferior Demiface Topography

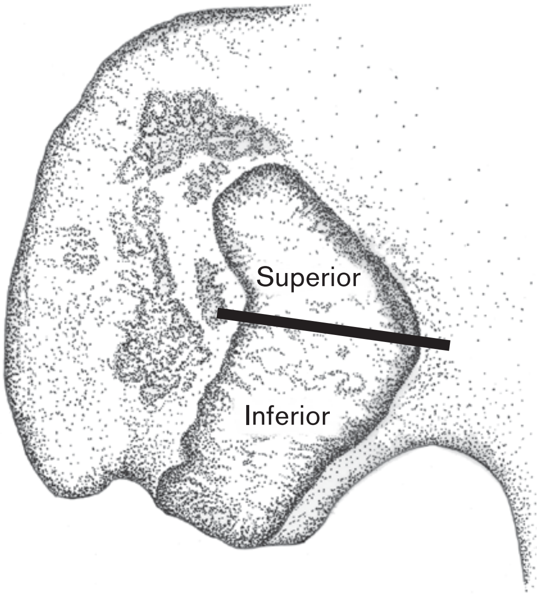

Figure 2.30. ***Superior Demiface Topography***

LOCATION

The inferior demiface is examined. The superior and inferior demifaces are divided by a line extending posteriorly from the most anterior point of the apex to the posterior border of the joint (see Figure 2.30).

CHARACTERISTICS [STAGES]

1. Undulating
2. Median elevation
3. Flat to Irregular

DEFINITIONS

1. *Undulating*: The surface is undulating in a superior to inferior direction (Figure 2.34). There is no centrally located linear area of elevated bone (*Median Elevation*). When the entire articular surface is viewed in aggregate, the overall effect is of two or three low waves proceeding lengthwise along the joint.

2. *Median elevation*: In the middle to posterior part of the demiface there is a broad raised area where the joint surface is elevated slightly above the rest of the joint (Figure 2.35). The elevation is flanked anteriorly, posteriorly, or both by one or two long, low areas. The elevated area takes the form of an elongated ridge with the long axis paralleling the main orientation of the demiface. It is not unusual for the elevated area to be restricted to a noticeably raised area that does not extend the entire length of the demiface. To be scored as present, the elevated area must extend along at least one-third of the joint surface's length

3. *Flat to irregular*: The surface is essentially flat or recessed, a result of marginal lipping, or it is irregular from degeneration of the joint or the formation of low, pillow-like exostoses (Figure 2.36).

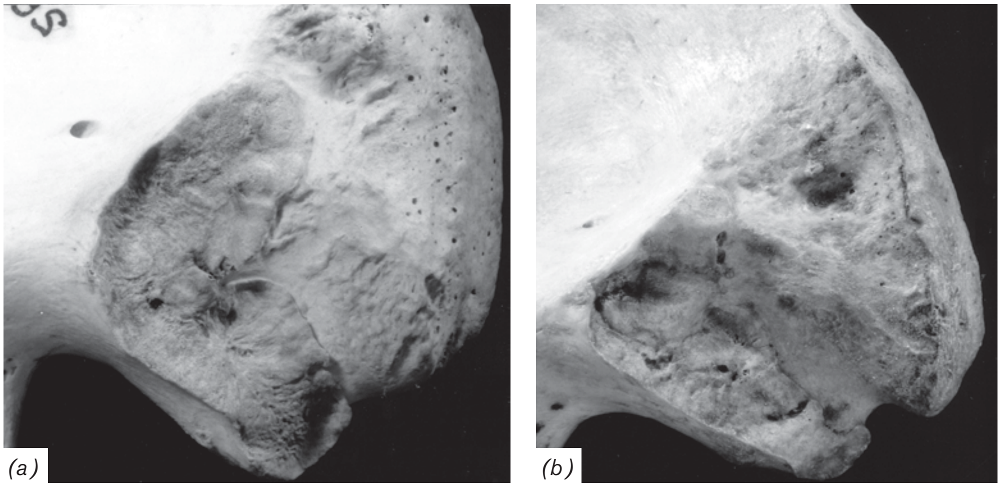

Figure 2.34.
(a) Undulating *[1], inferior part (T 269, image reversed),*
(b) Undulating *[1], inferior part (T 385, image reversed)*

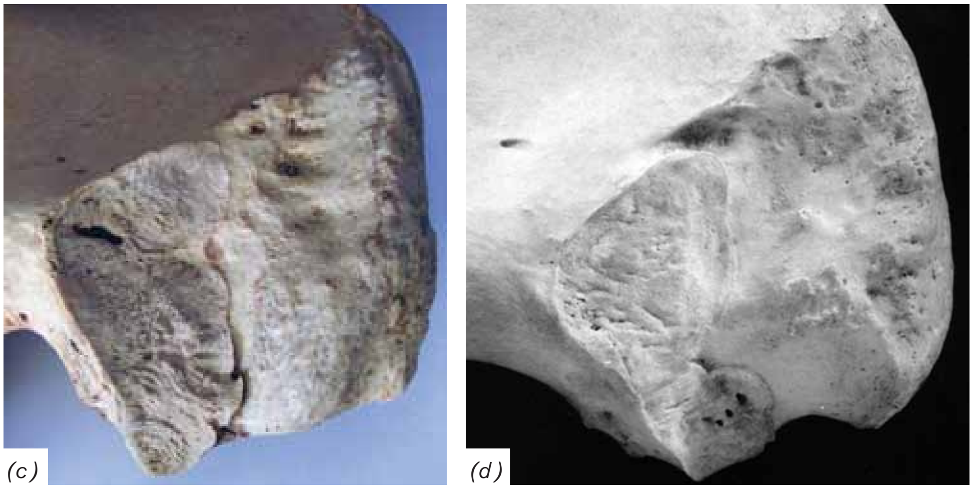

Figure 2.34. ***(c) Undulating*** *[1], inferior and superior (subtle) parts (P 5897), and* ***(d) Undulating*** *[1], inferior part (T 255).*

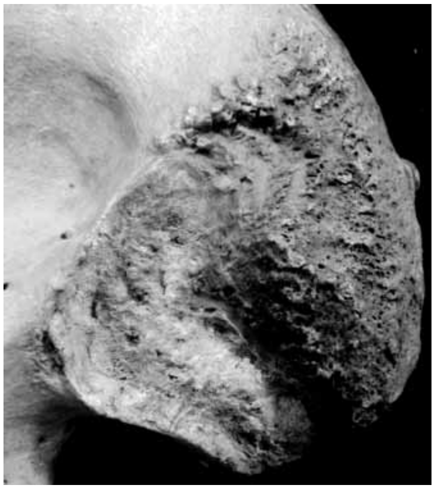

Figure 2.35.
Median elevation *[2], inferior part (T 274, image reversed).*

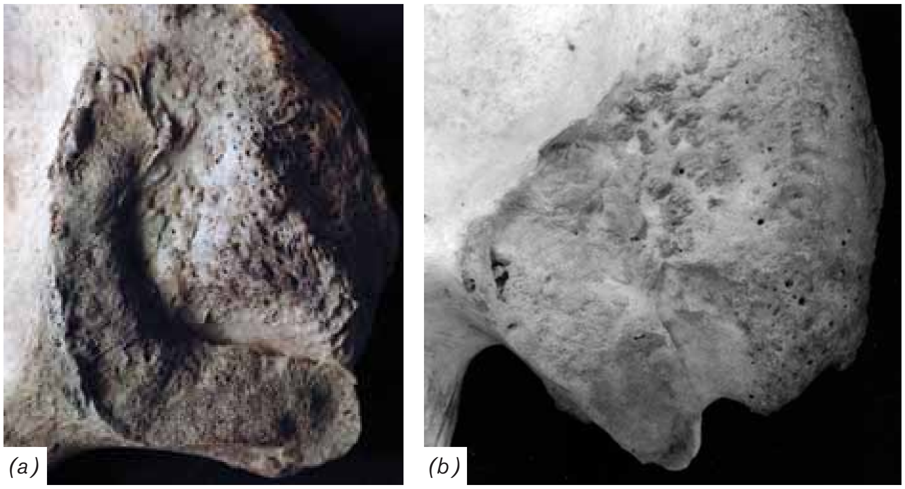

Figure 2.36.
(a) Flat *[3], inferior part (MC 6-73) and* ***(b) Flat*** *[3], superior and inferior parts (T 500).*

III. Superior Surface Characteristics

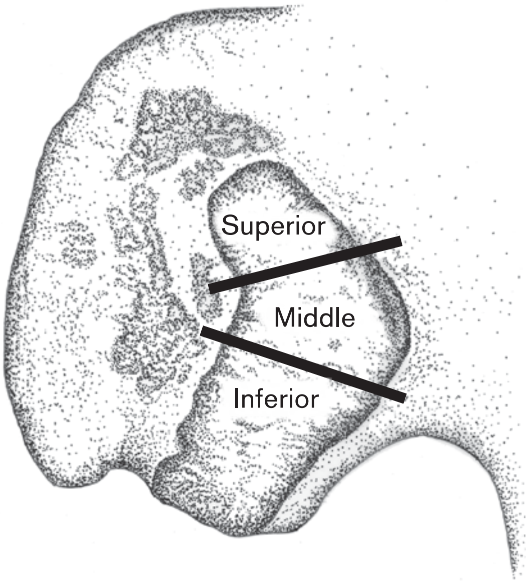

Figure 2.37. ***Superior Surface Characteristics***

LOCATION

The superior part of the face is examined. The joint surface is divided into superior, middle (apical), and inferior segments (Figure 2.37).

CHARACTERISTICS [STAGES]

1. **>2/3 covered by billows**
2. **1/3-2/3 covered by billows**
3. **<1/3 covered by billows**
4. **Flat (no billows)**
5. **Bumps**

DEFINITIONS

1. *Billows cover >2/3 of the surface*: Low, rounded ridges separated by furrows with have distinctly rounded bases are clearly identifiable (Figure 2.38). The ridge surfaces are curved from the depths of the furrows completely across their crests. Most or all of the billowing is oriented roughly anterior to posterior, and furrows can run across much of the face. Billowing covers most (>2/3) of the joint surface (i.e., it is a dominant element of the surface).

2. *Billows cover 1/3-2/3 of the surface*: About one-half of the surface is covered by billows.

3. *Billows cover <1/3 of the surface*: Billows are a noticeable, but minor, component of the joint surface. The rest of the surface is flat or bumpy.

4. *Flat* (no billows): The joint surface is flat (Figure 2.39).

5. *Bumps*: Most, or all, of the joint surface is covered by low, rounded bony exostoses, much like little irregular pillows (Figure 2.40). Part of the surface may be flat, but over one-half of it is bumpy. One is often reminded of lentils squished onto the joint surface. The bumps can be discrete low elevations or confluent, in which case the raised areas have irregular margins

Unscorable: If defects in the joint surface are so extensive they obscure much of the face, this characteristic is considered unscorable. The defects often take one of two forms. (1) Irregular and large pits can be present that are for the most part either separate or confluent with one another. The pits can be found anywhere on the joint face. (2) Alternatively, the defects are linear grooves that occur in isolation or as multiple nearby grooves. In either case, they can be up to a centimeter long, and they generally extend in a transverse direction. For the linear defects in particular, the smooth bone of the joint surface frequently laps over into the defect for a short distance. That is, the lips of these defects are covered by smooth-surfaced bone. The grooves are more commonly found in the middle part of the joint surface than toward the superior or inferior parts. They are not to be confused with some form of anomalously deep billows. Occasionally surface defects are sufficient to obscure the **Superior and Inferior Demiface Topography**, although they more often interfere with the proper scoring of the **Superior**, **Middle**, and **Inferior Surface Characteristics**.

SCORING TIPS

These comments pertain to the **Superior**, **Middle**, and **Inferior Surface Characteristics**. The best way to proceed is to first

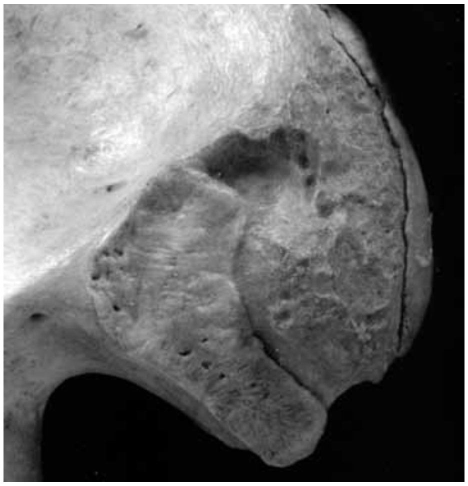

Figure 2.38.
***Billows** [1-3], middle apical and inferior parts (T-1023).*

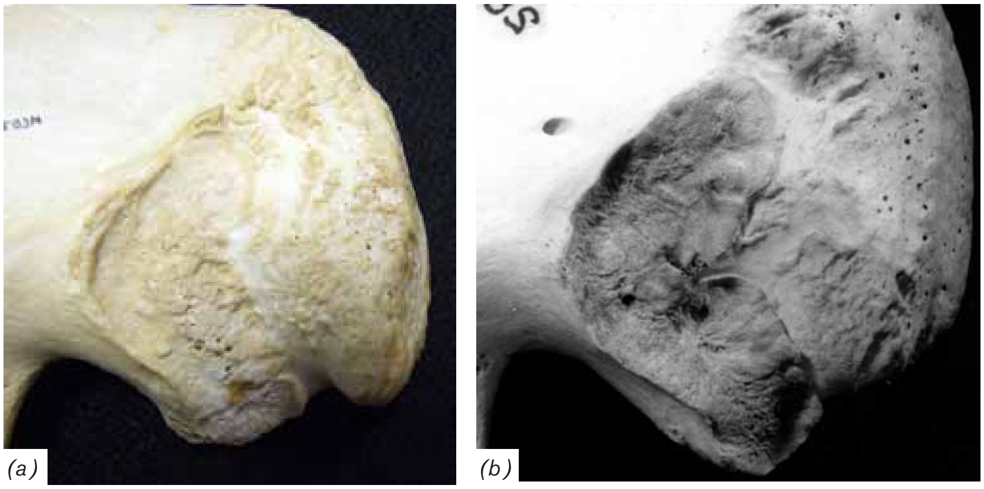

(a) (b)

Figure 2.39.
***(a) Flat** [4], superior part (MC 7-55, image reversed) and **(b) Flat** [4], superior part (T 269, image reversed).*

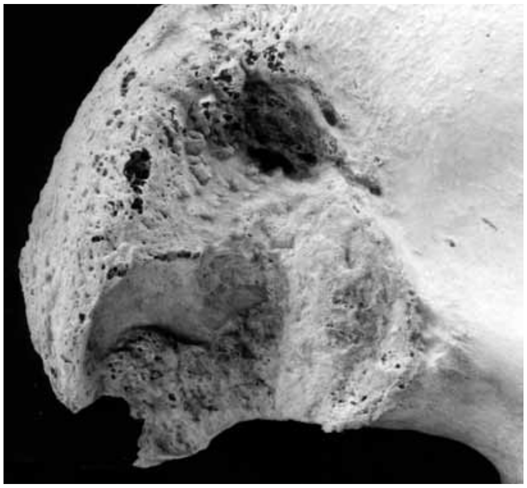

Figure 2.40.
***Bumps** [5] superior part (T-1103R, image reversed).*

bracket the part of the auricular surface that includes the apex with your two thumbs. That defines the middle, or apical, portion. Then move one thumb to define the inferior margin of the superior portion. Finally, use a thumb to define the superior margin of the inferior surface. This simple masking procedure will help counteract the tendency to be influenced by what is present on adjacent parts of the auricular surface.

When scoring the surface characteristics, it is generally easiest to look for billows first. If present, then determine how abundant they are: there are only a few of them, they cover roughly one-half of the surface, or they spread out across much of the surface. If billows are not present, then look for bumps. The low and irregular bumps do not display the generally transverse organization of the billows.

IV. Middle Surface Characteristics

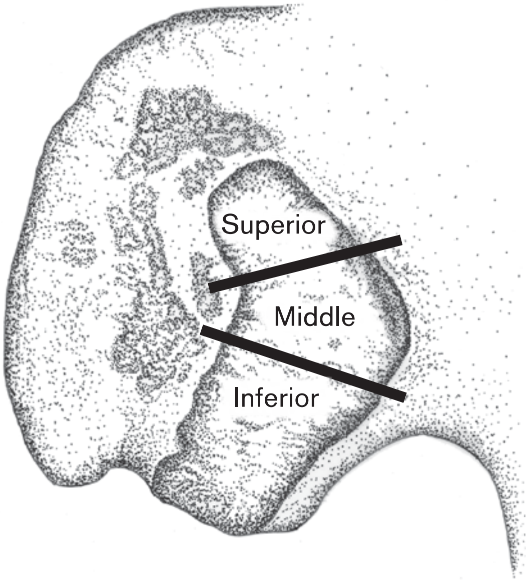

Figure 2.37. ***Superior Surface Characteristics***

LOCATION

The middle part of the face is examined (see Figure 2.37). This area corresponds to the portion of the joint surface that includes the so-called apex – the curved part of the boomerang-shaped auricular surface.

CHARACTERISTICS [STAGES]

1. **>2/3 covered by billows**
2. **1/3-2/3 covered by billows**
3. **<1/3 covered by billows**
4. **Flat (no billows)**
5. **Bumps**

DEFINITIONS

1. *Billows cover >2/3 of the surface*: Low, rounded ridges separated by furrows that have distinctly rounded bases are clearly identifiable (Figure 2.41). The ridge surfaces are curved from the depths of the furrows completely across their crests. Most or all of the billowing is oriented roughly anterior to posterior, and furrows can run across much of the face. Billowing covers most (>2/3) of the joint surface (i.e., it is a dominant element of the surface).

2. *Billows cover 1/3-2/3 of the surfac*e: About one-half of the surface is covered by billows.

3. *Billows cover <1/3 of the surface*: Billows are a noticeable, but minor, component of the joint surface. The rest of the surface is flat or bumpy

4. *Flat (no billows)*: The joint surface is flat (Figure 2.42).

5. *Bumps*: Most, or all, of the joint surface is covered by low, rounded bony exostoses, much like little irregular pillows (Figure 2.43). Part of the surface may be flat, but over one-half of it is bumpy. One is often reminded of lentils squished onto the joint surface. The bumps can be discrete low elevations or confluent, in which case the raised areas have irregular margins.

Unscorable: If defects in the joint surface are so extensive they obscure much of the face, this characteristic is considered unscorable. The defects often take one of two forms. (1) Irregular and large pits can be present that are for the most part either separate or confluent with one another. The pits can be found anywhere on the joint face. (2) Alternatively, the defects are linear grooves that occur in isolation or as multiple nearby grooves. In either case, they can be up to a centimeter long, and they generally extend in a transverse direction. For the linear defects in particular, it frequently appears as if the smooth bone of the joint surface laps over into the defect for a short distance. The grooves are more commonly found in the middle part of the joint surface than toward the superior or inferior ends. They are not to be considered a form of anomalously deep billows. Occasionally the surface defects are sufficient to obscure the **Superior and Inferior Demiface Topography**, although they more often interfere with the proper scoring of the **Superior**, **Middle**, and **Inferior Surface Characteristics**.

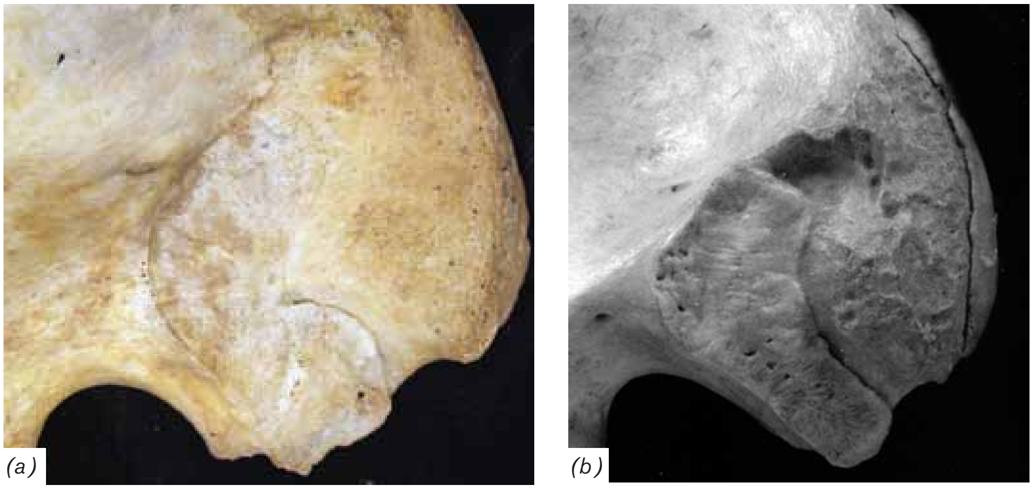

Figure 2.41.
(a) Billows *[1-3], middle/apical part (NF 225, image reversed) and* ***(b) Billows*** *[1-3], middle apical and inferior parts (T-1023).*

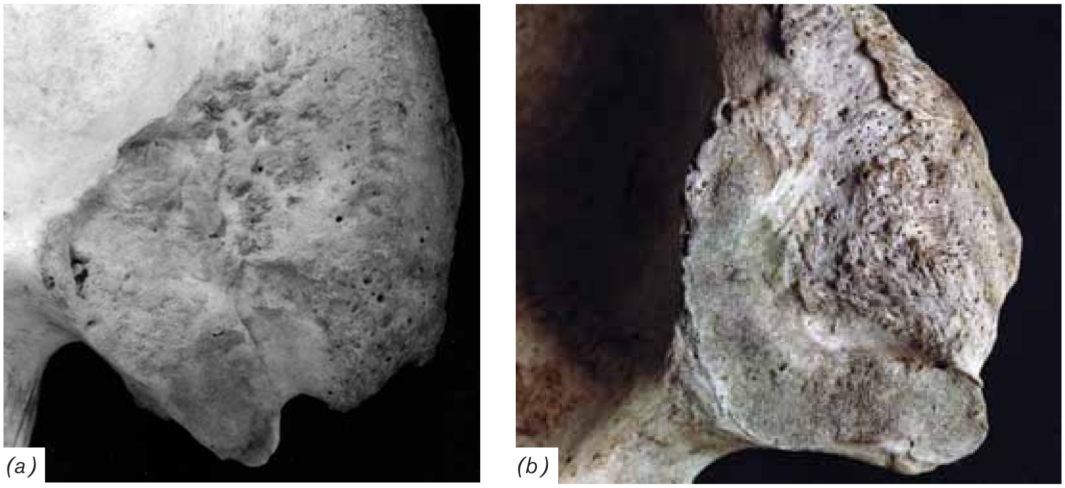

Figure 2.42.
(a) ***Flat*** *[4], middle/apical and inferior parts (T-500) and (b) Flat [4] (MC 6-73).*

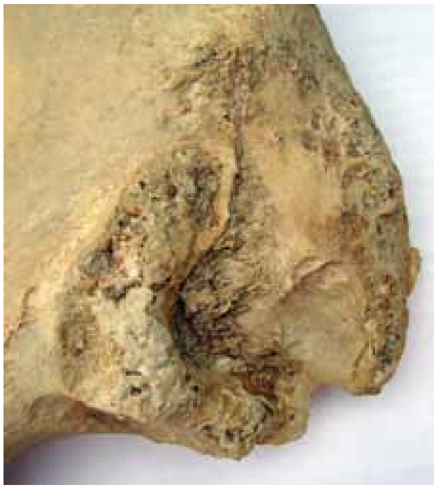

Figure 2.43. ***Bumps*** *[5] middle part (P 5750, image reversed).*

V. Inferior Surface Characteristics

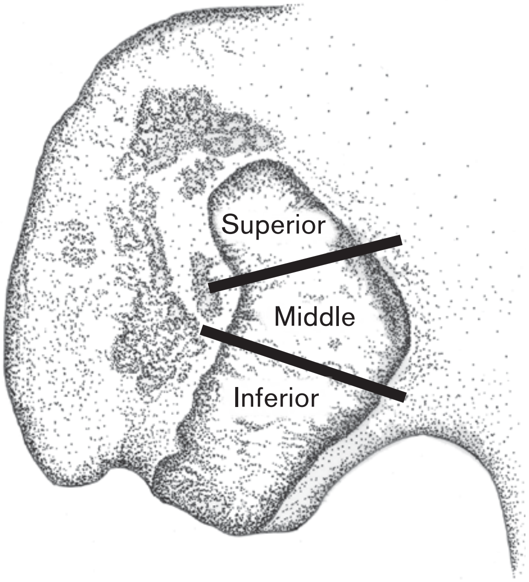

Figure 2.37. ***Superior Surface Characteristics***

LOCATION

The inferior part of the face is examined. On most ilia this is the largest part of the auricular surface as it is defined here (see Figure 2.37).

CHARACTERISTICS [STAGES]

1. **>2/3 covered by billows**
2. **1/3-2/3 covered by billows**
3. **<1/3 covered by billows**
4. **Flat (no billows)**
5. **Bumps**

DEFINITIONS

1. *Billows cover >2/3 of the surface*: Low, rounded ridges separated by furrows with distinctly rounded bases are clearly identifiable (Figure 2.44). The ridge surfaces are curved from the depths of the furrows completely across their crests. Most or all of the billowing is oriented roughly anterior to posterior, and furrows can run across much of the face. Billowing covers most (>2/3) of the joint surface (i.e., it is a dominant element of the surface).
2. *Billows cover 1/3-2/3 of the surface*: About one-half of the surface is covered by billows.
3. *Billows cover <1/3 of the surface*: Billows are a noticeable, but minor, component of the joint surface. The rest of the surface is flat or bumpy.
4. *Flat (no billows)*: The joint surface is flat (Figure 2.45).
5. *Bumps*: Most, or all, of the joint surface is covered by low, rounded bony exostoses, much like little irregular pillows (Figure 2.46). Part of the surface may be flat, but over one-half of it is bumpy. One is often reminded of lentils squished onto the joint surface. The bumps can be discrete low elevations or confluent, in which case the raised areas have irregular margins.

Unscorable: If defects in the joint surface are so extensive they obscure much of the face, this characteristic is considered unscorable. The defects often take one of two forms. (1) Irregular and large pits can be present that are for the most part either separate or confluent with one another. The pits can be found anywhere on the joint face. (2) Alternatively, the defects are linear grooves that occur in isolation or as multiple nearby grooves. In either case, they can be up to a centimeter long, and they extend in a generally transverse direction. For the linear defects in particular, it frequently appears as if the smooth bone of the joint surface laps over into the defect for a short distance. The grooves are more commonly found in the middle part of the joint surface than toward the superior or inferior ends. They are not to be considered a form of anomalously deep billows. Occasionally the surface defects are sufficient to obscure the **Superior and Inferior Demiface Topography**, although they more often interfere with the proper scoring of the **Superior**, **Middle**, and **Inferior Surface Characteristics.**

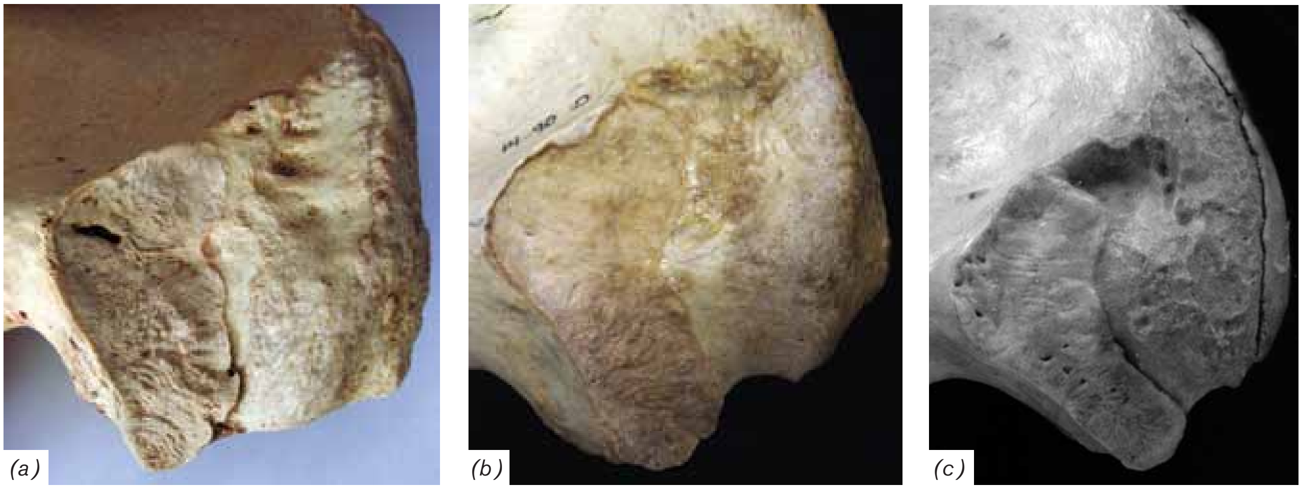

Figure 2.44.
***(a) Billows** [1-3], inferior part (P 5897), **(b) Billows** [1-3], inferior part (Bass 14-90), and **(c) Billows** [1-3], middle/ apical and inferior parts (T-1023).*

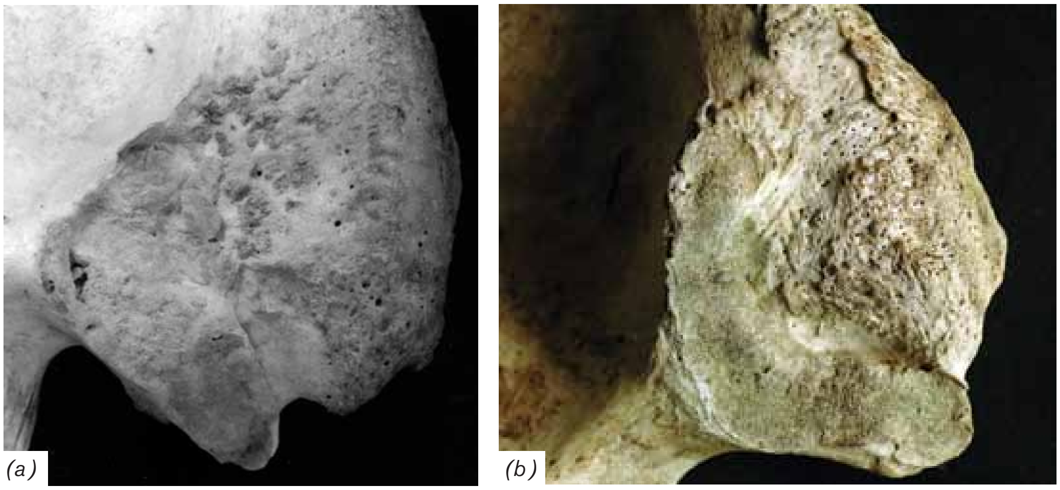

Figure 2.45.
***(a) Flat** [4], apical and inferior parts (T-500) and **(b) Flat** [4] (MC 6-73).*

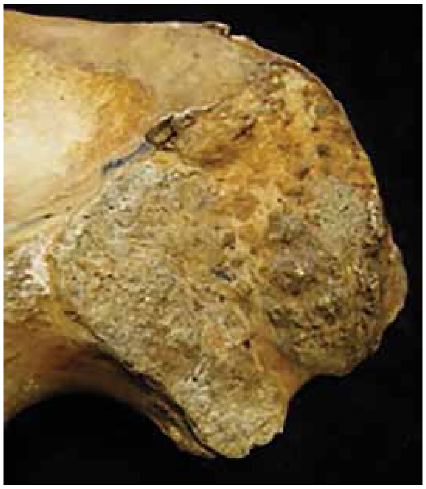

*Figure 2.46. **Bumps** [5] on inferior part (William M. Bass Collection).*

VI. Inferior Surface Texture

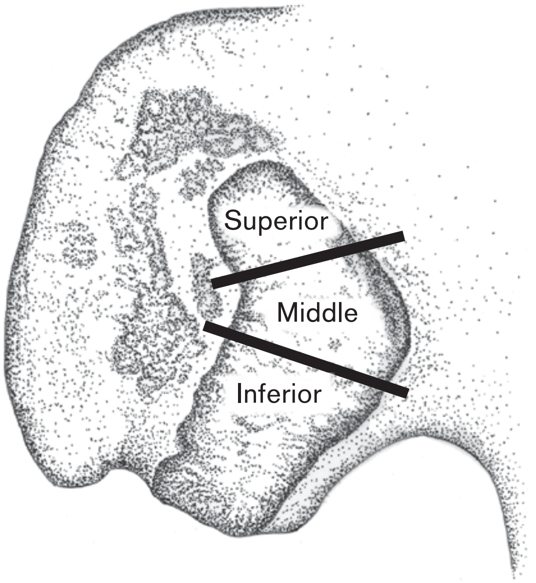

Figure 2.47. ***Inferior Surface Texture***

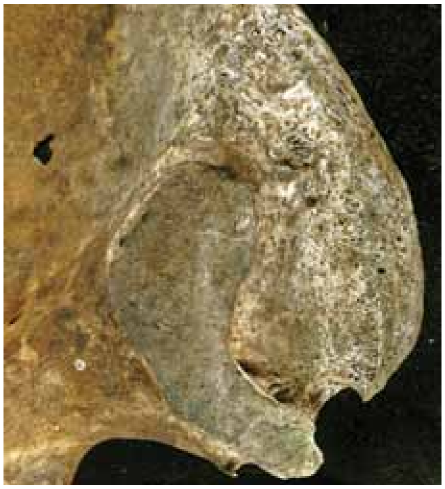

Figure 2.48. ***Smooth*** *[1] (MC 6-69).*

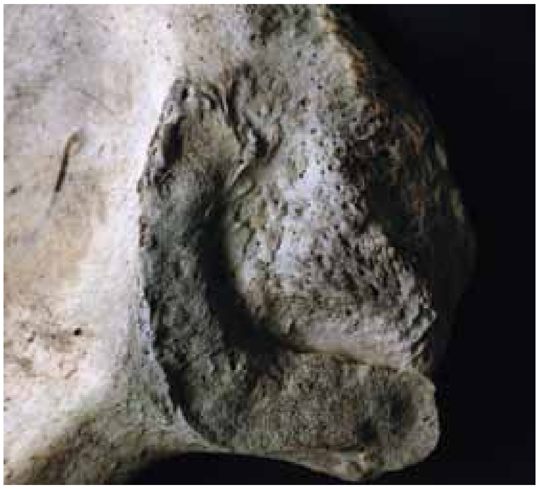

Figure 2.49. ***Microporosity*** *[2] (MC 6-73).*

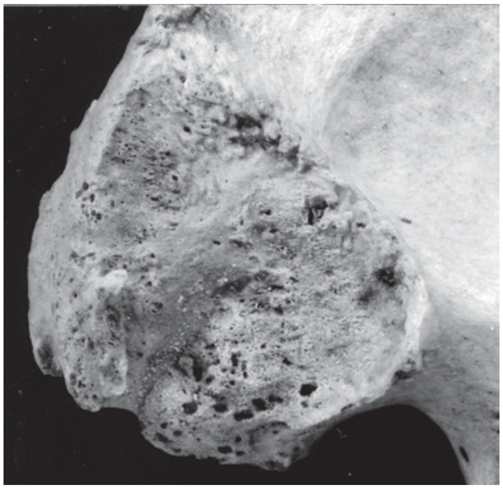

Figure 2.50. ***Macroporosity*** *[3] (T 1394, image reversed).*

LOCATION

Only one part of the joint surface – the inferior area – is scored for texture (Figure 2.47). This part of the joint is 1 cm long, as measured in a superior to inferior direction. Occasionally there can be an elongated portion of the joint surface, often accompanied by marginal lipping, that extends well beyond the main body of the ilium. While it can occur in both sexes, it is more often seen in females. Do not score the auricular surface that extends beyond the margin of the ilium proper.

CHARACTERISTICS [STAGES]

1. Smooth
2. Microporosity
3. Macroporosity

DEFINITIONS

1. *Smooth*: Most, or all, of the joint surface appears to be smooth to slightly granular (Figure 2.48).
2. *Microporosity*: At least one-half of the surface has a porous appearance with apertures less than 0.5 mm in diameter (Figure 2.49). The symphyseal face looks as if it is covered by many closely spaced pinpricks.
3. *Macroporosity*: At least one-half of the surface is porous, with most or all of the apertures exceeding 0.5 mm in diameter (Figure 2.50). Here the surface looks as if it was penetrated by multiple closely spaced pinheads (the large end of the same shirt pin mentioned above).

VII. Superior Posterior Iliac Exostoses

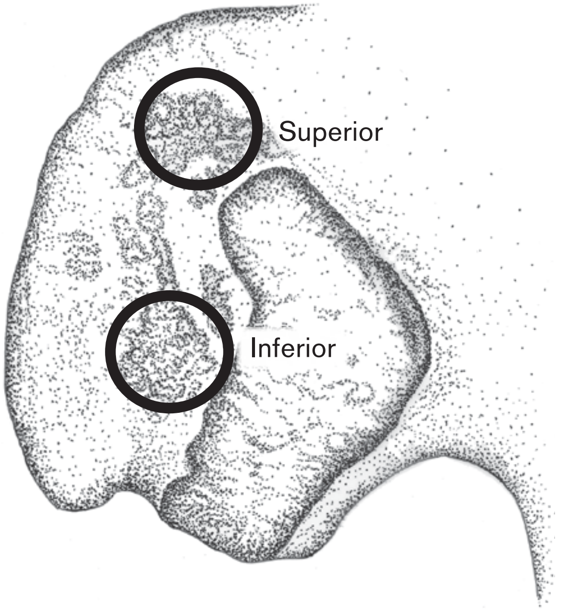

Figure 2.51. ***Superior Posterior Iliac Exostoses***

LOCATION

Superior posterior iliac exostoses are scored. This area refers to the superior part of the medial surface of the posterior ilium where ligaments attach (Figure 2.51). It is located superior to the sacroiliac joint surface; that is, to a line that passes from the anterior superior iliac spine, to the most superior point of the joint surface (the superior angle), and on through the posterior part of the ilium. In some individuals, the bone is distinctly raised in this area, so care must be taken to differentiate jagged (or high) exostoses from rounded or pointed ones perched on top of a raised elevation of bone.

CHARACTERISTICS [STAGES]

1. Smooth
2. Rounded exostoses
3. Pointed exostoses
4. Jagged exostoses
5. Touching exostoses
6. Fusion

DEFINITIONS

1. *Smooth*: The surface is often elevated in this area, but shows no evidence of discrete bony elevations (Figure 2.52). At most there are one or two isolated small exostoses.
2. *Rounded exostoses*: Definite raised areas of bone with rounded crests dominate the scoring area (Figure 2.53).
3. *Pointed exostoses*: Over one-half of the rough area where ligaments attach is dominated by sharply pointed, but short elevations of bone (Figure 2.54).
4. *Jagged exostoses*: The raised areas of bone have a jagged appearance where ligaments attach in life (Figure 2.55). These exostoses, which can have either round or sharp ends, are tall, extending several millimeters upward from the original bone surface.
5. *Touching exostoses or facet*: There is a distinct oval facet where the ilium and sacrum are in close justaposition, often 5 mm in diameter or more. This facet can occur either on top of a pronounced growth of bone or on an iliac surface that is relatively flat.
6. *Fusion*: The ilium and sacrum are fused by exostoses in this area.

SCORING TIPS

These comments pertain equally to the **Superior and Inferior Posterior Iliac Exostoses**. If the ilium is fused to the sacrum at the margins of the auricular surface (usually the superior-anterior margin), you should score the **Superior and Inferior Exostoses** as not observable. The effect of fusion on the posterior exostoses is unknown. The common adult condition is to have either *Rounded* or *Pointed Exostoses*. The *Smooth* variant is typically found on the ilia of very young adults. You will rarely see sharp *Jagged Exostoses.*

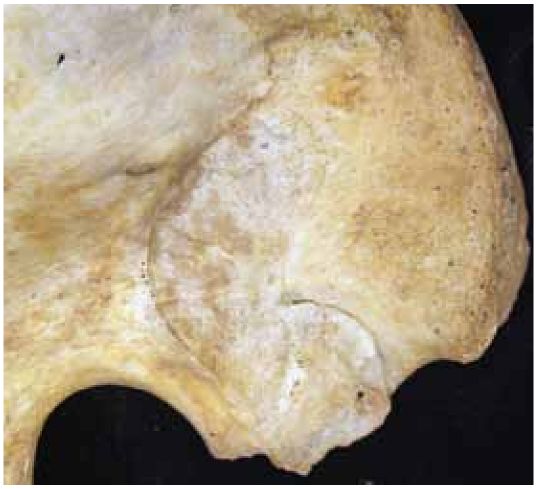

Figure 2.52. ***Smooth*** *[1] superior exostoses (NF 225, image reversed).*

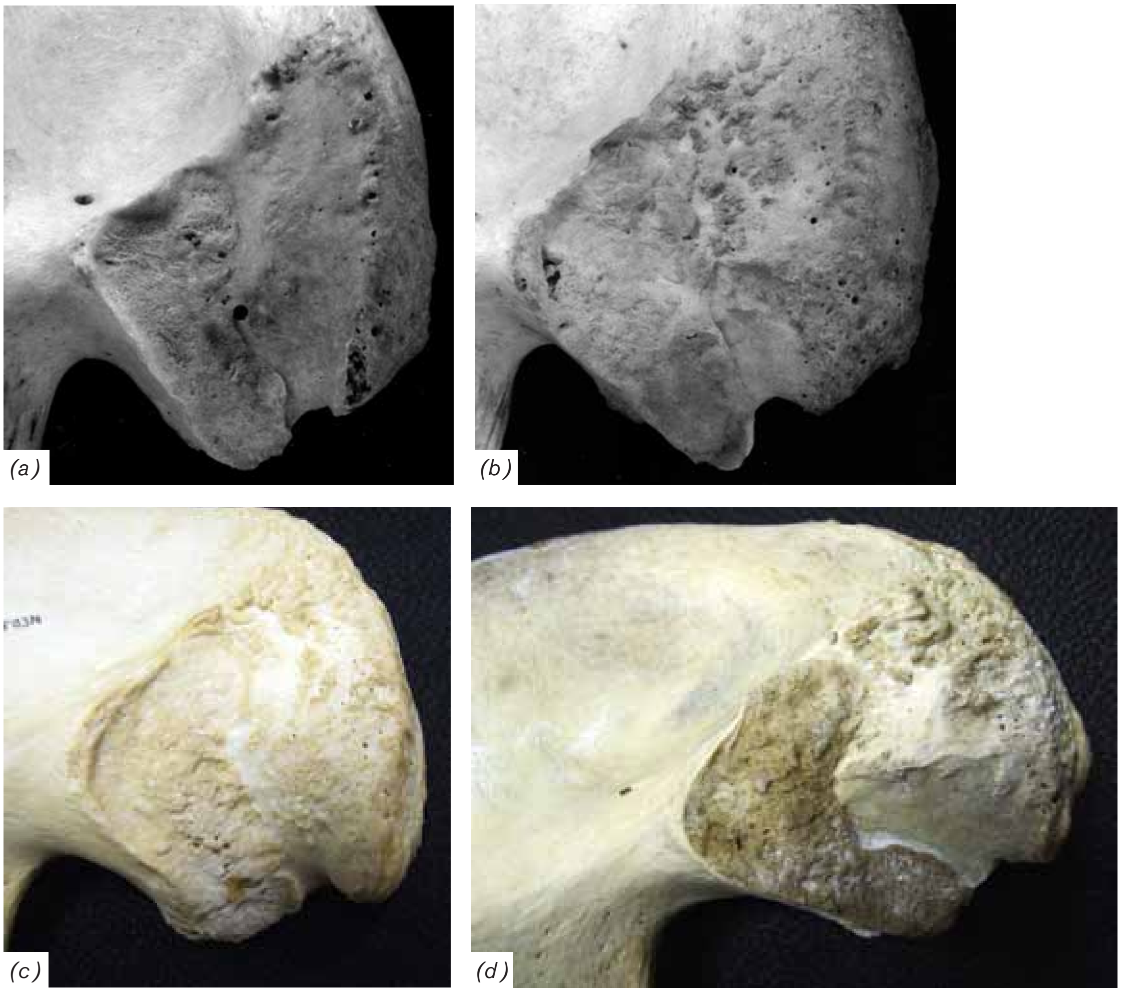

Figure 2.53.
(a) Rounded *[2], superior (T 862),* ***(b) Rounded*** *[2], superior exostoses (T 500),* ***(c) Rounded*** *[2], superior and inferior exostoses (MC 7-55, image reversed), and* ***(d) Rounded*** *[2], superior (MC 7-76*

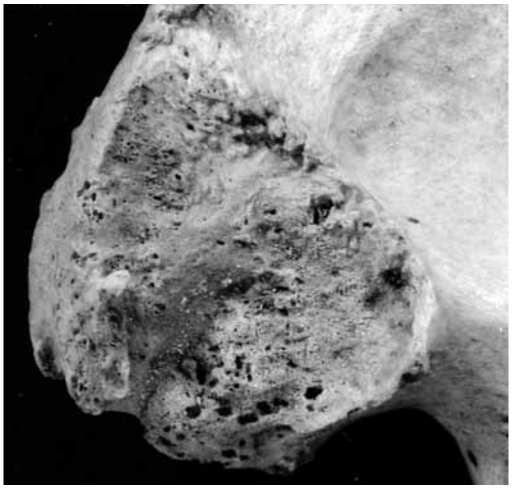

Figure 2.54.
Pointed *[3], superior (T 1394, image reversed).*

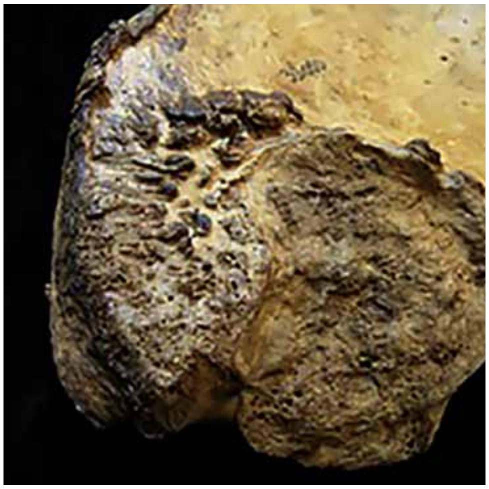

Figure 2.55. ***Jagged.***

VIII. Inferior Posterior Iliac Exostoses

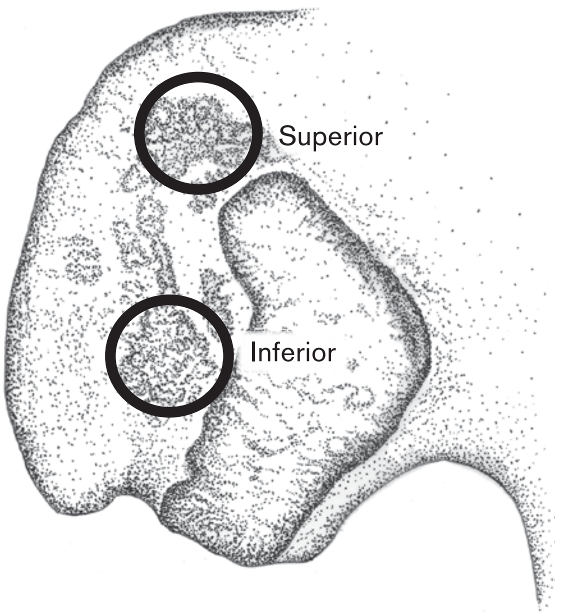

Figure 2.51. ***Superior Posterior Iliac Exostoses***

LOCATION

The inferior posterior iliac exostoses are scored. This area refers to the inferior part of the medial surface of the posterior ilium where ligaments attach (see Figure 2.51). It is located inferior to a line that passes from the anterior superior iliac spine, to the most superior point of the sacroiliac joint surface (the superior angle), and on through the posterior part of the ilium. This area is located immediately posterior to the middle of the sacroiliac joint; that is, it lies behind the most anteriorly projecting part of the posterior margin of the joint. In some individuals, the bone is distinctly raised in this area, so care must be taken to differentiate jagged (or high) exostoses from rounded or pointed ones perched on top of a raised elevation of bone.

CHARACTERISTICS [STAGES]

1. **Smooth**
2. **Rounded exostoses**
3. **Pointed exostoses**
4. **Jagged exostoses**
5. **Touching exostoses**
6. **Fusion**

DEFINITIONS

1. *Smooth*: The surface is often elevated in this area, but shows no evidence of discrete bony elevations (Figure 2.56). At most there are one or two isolated small exostoses.
2. *Rounded exostoses*: Definite raised areas of bone with rounded crests dominate the scoring area (Figure 2.57).
3. *Pointed exostoses*: Over one-half of the rough area where ligaments attach is dominated by sharply pointed but short elevations of bone (Figure 2.58).
4. *Jagged exostoses*: The raised areas of bone have a jagged appearance where ligaments attach in life. These exostoses, which can have either round or sharp ends, are tall, extending several millimeters upward from the original bone surface.
5. *Touching exostoses or facet*: There is a distinct oval facet where the ilium and sacrum are in close justaposition, often 5 mm in diameter or more (Figure 2.59). This facet can occur either on top of a pronounced growth of bone or on an iliac surface that is relatively flat.
6. *Fusion*: The ilium and sacrum are fused by exostoses in this area.

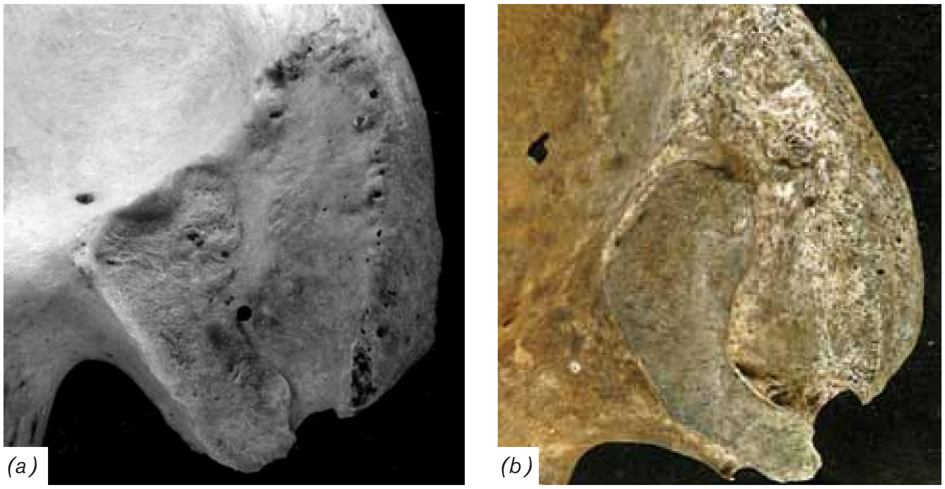

Figure 2.56. ***(a) Smooth*** *[1], inferior (T 862) and* ***(b) Smooth*** *[1], inferior exostoses (MC).*

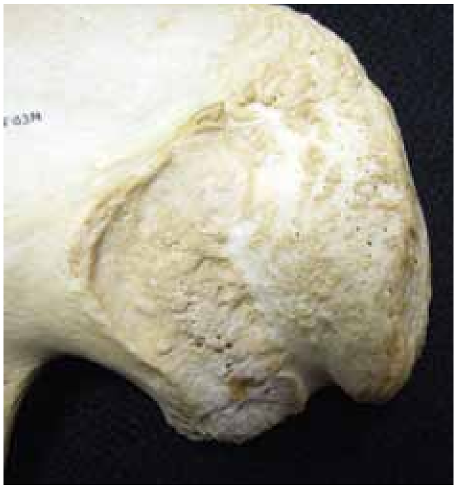

Figure 2.57. ***Rounded*** *[2], superior and inferior exostoses (MC 7-55, image reversed).*

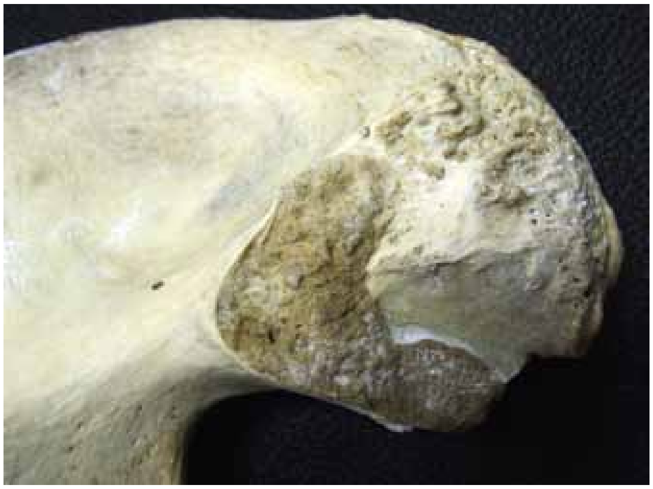

Figure 2.58. ***Pointed*** *[3], inferior, sometimes sharp ones can approximate linear ridges (MC 7-76).*

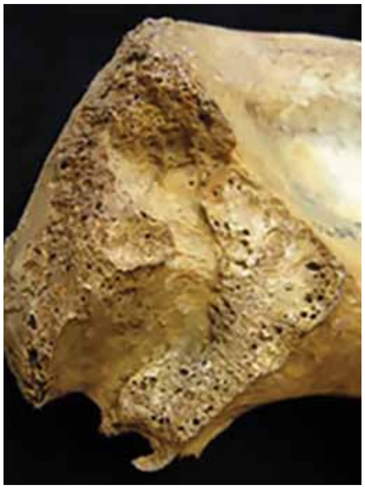

Figure 2.59. ***Facet*** *[5] on inferior portion (William M. Bass Collection)*

IX. Posterior Exostoses

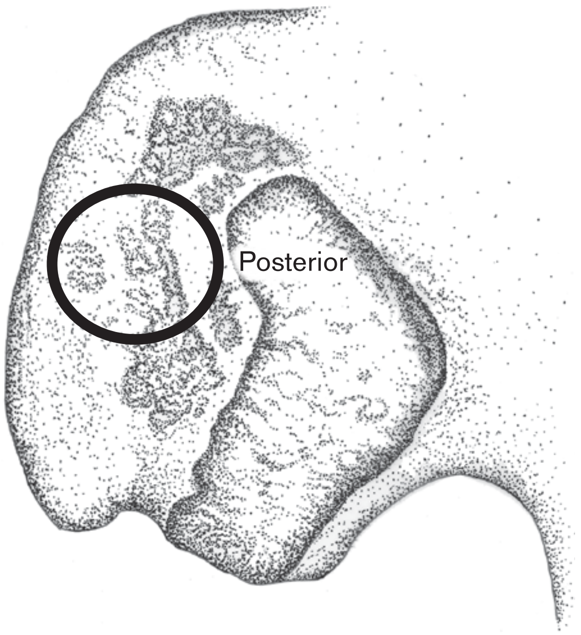

Figure 2.60. ***Posterior Exostoses***

LOCATION

The posterior iliac area between the **Superior and Inferior Posterior Iliac Exostoses** (defined above) is scored. These exostoses are found on the medial side of the ilium bordered posteriorly by the iliac crest, anteriorly by the sacroiliac joint surface, superiorly by a slightly raised area often surmounted by bony exostoses (**Superior Posterior Iliac Exostoses**), and inferiorly by a similar area (**Inferior Posterior Iliac Exostoses**) (Figure 2.60). Most individuals are *Smooth* as defined below. The feature is best considered an old-age trait.

CHARACTERISTICS [STAGES]

1. Smooth (no exostoses)
2. Rounded exostoses
3. Pointed exostoses

DEFINITIONS

1. *Smooth*: The area posterior to the sacroiliac joint is smooth, except for the two areas scored separately as **Superior and Inferior Posterior Iliac Exostoses** (Figure 2.61).

2. *Rounded exostoses*: Low, rounded exostoses (or spicules) cover the *entire* bone surface posterior to the sacroiliac joint, except for a ca. 0.5 cm band of smooth bone immediately adjacent to the posterior edge of the joint (Figure 2.62). The exostoses are normally lower than the **Superior and Inferior Posterior Iliac Exostoses**. The low exostoses give the normally smooth iliac surface a rough appearance. It looks as if the surface is covered by coarse (construction) sand.

3. *Pointed exostoses*: Low, pointed exostoses (or spicules) cover the *entire* bone surface posterior to the sacroiliac joint, except for a ca. 0.5 cm band of smooth bone immediately adjacent to the posterior edge of the joint. The exostoses are normally lower than the **Superior and Inferior Posterior Iliac Exostoses**. The sharp exostoses give the normally smooth iliac surface a rough appearance. It looks as if the surface is covered by coarse (construction) sand.

Not scorable: Superior part of sacroiliac joint surface cannot be scored because of significant abnormal pitting (Figure 2.63).

SCORING TIPS

By far the most common condition is *Smooth*, as it is defined here. Keep in mind that the *Smooth* category also includes surfaces with bony exostoses, which are typical of the 20s onward. Bones are only scored as *Rounded* or *Pointed* if the entire surface is covered with exostoses (the surface looks as if it is thickly covered by construction sand, and little, if any, of the original smooth surface remains visible). Furthermore, the ilium and sacrum can be fused in several places. If that occurs, you should score **Posterior Exostoses** as not observable, as the effect of fusion on this joint characteristic is unknown.

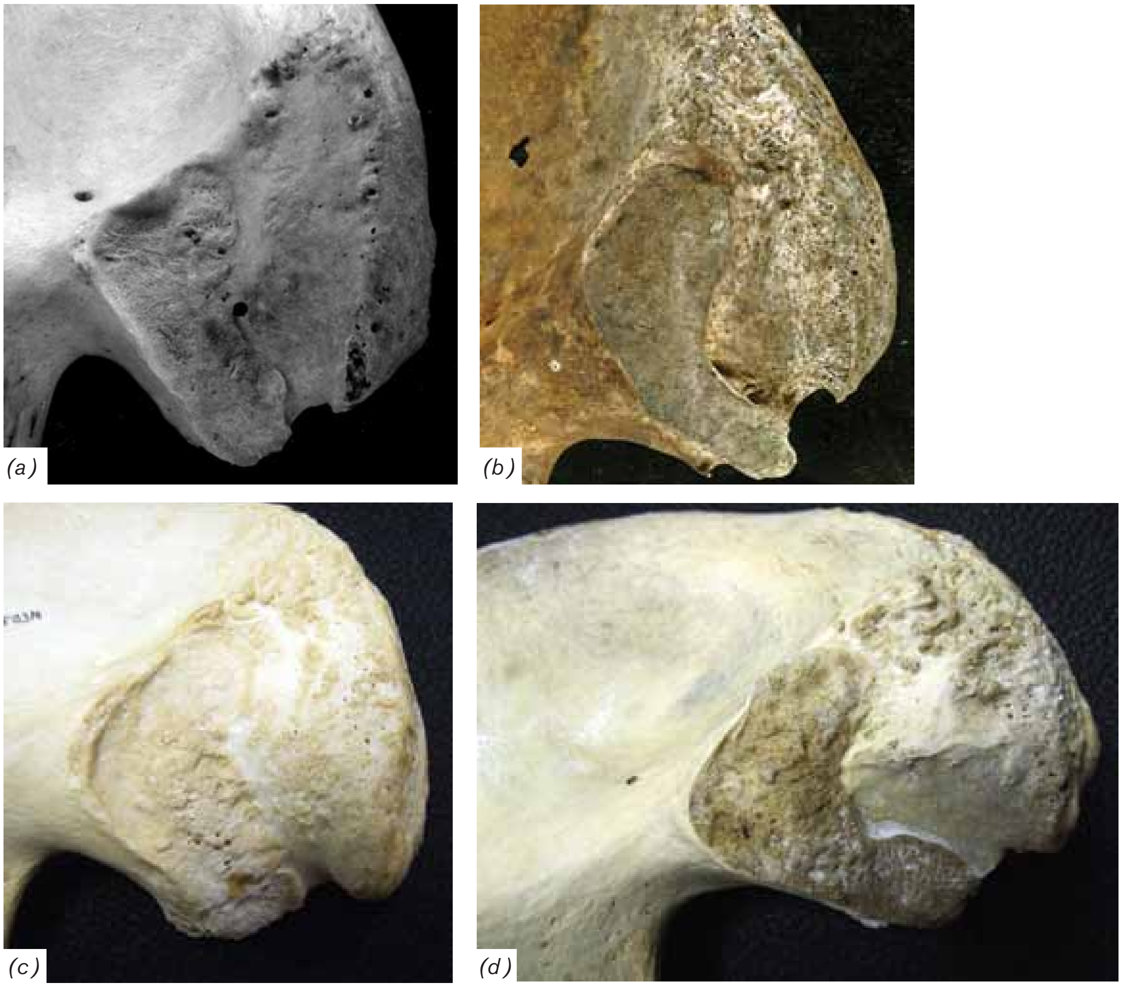

Figure 2.61.
***(a) Smooth** [1] (T 862), **(b) Smooth** [1] (MC 6-69), **(c) Smooth** [1], while exostoses are present, significant areas of smooth bone remain (MC 7-55, image reversed), and **(d) Smooth** [1], while exostoses are present, significant areas of smooth bone remain (MC 7-76).*

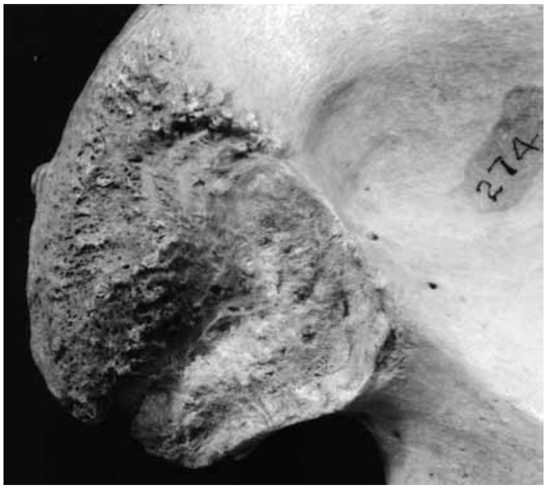

*Figure 2.62. **Rounded** [2] (T 274, image reversed).*

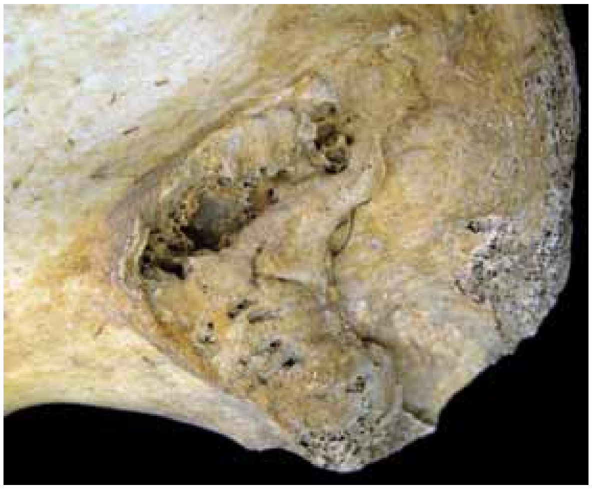

*Figure 2.63. **Sacroiliac joint**, not scorable (NF 229, image reversed).*

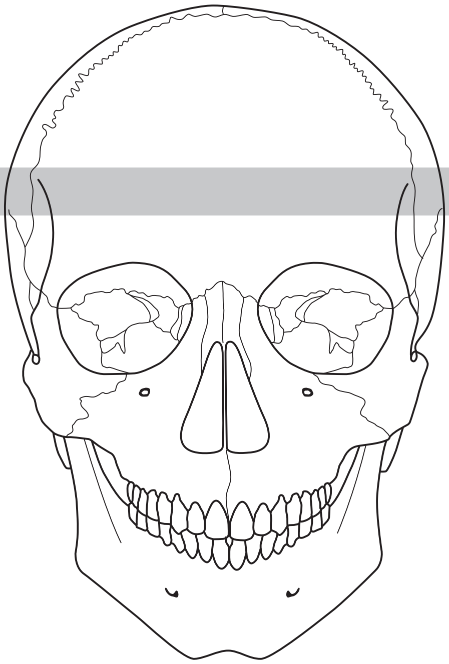

Cranial Sutures

The suture closure scores are similar to what osteologists have used for over a century. Ectocranial suture closure is recorded because it is often difficult to examine the interiors of archaeological crania, which can be dirty. Suture segment names conform to those commonly used by osteologists. Palatal sutures are included because they have been shown to be of some use in age estimation, even though they are often damaged (Mann et al. 1987). Five sutures are scored:

I. Coronal Pterica
II. Sagittal Obelica
III. Lambdoidal Asterica
IV. Zygomaticomaxillary
V. Interpalatine (Median Palatine, Posterior Portion)

SCORING TIPS

For sake of completeness, it is a good practice to record closure of both the left and right coronal, lambdoidal, and zygomaticomaxillary sutures, if present. When entering data, it is possible to list scores for both sides if they are different. Although Transition Analysis is based on the left side, the inclusion of scores for both bilateral sutures is conservative because it, in effect, increases the age interval. Because sutures are notoriously poor indicators of age, wider intervals are not a bad outcome.

The first stage, *Open*, is perhaps best seen in **Lambdoidal Asterica**. It is a common score for that suture segment, especially in young adults, but it even occurs in middle-aged adults. The *Open* category is distinguished from *Juxtaposed* by a noticeable gap between the two adjoining bones. The width of this gap varies from one suture to another, although it is generally greatest at **Lambdoidal Asterica**. While there is no bony bridging of the suture in the *Juxtaposed* category, the two adjacent bones are only separated by a narrow line, just as if a line was drawn on the bone by a pencil with a thin lead.

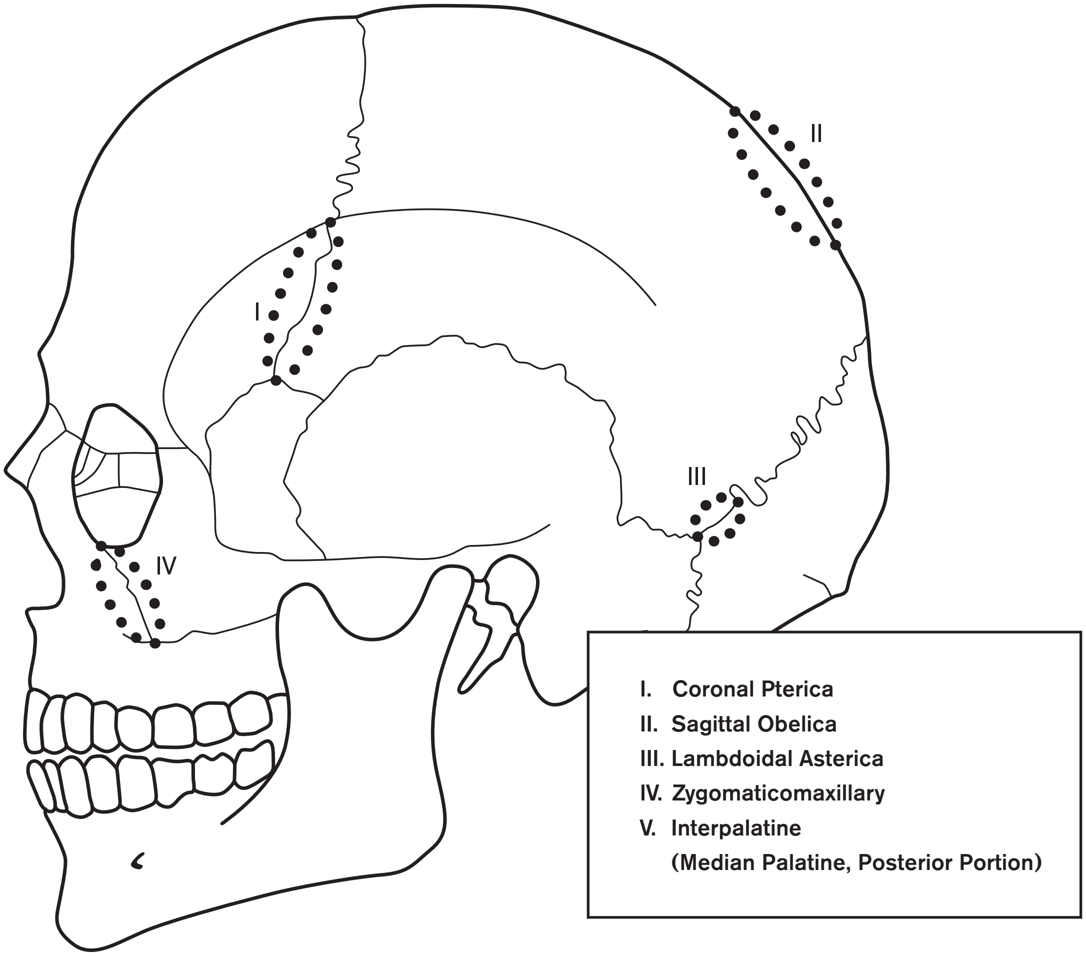

I. Coronal Pterica

LOCATION
Score the most inferior section of the coronal suture, a relatively straight part without a meandering appearance. It typically extends from the temporal line inferiorly to the sphenoid.

II. Sagittal Obelica

LOCATION
Score the relatively straight part of the posterior sagittal suture near the parietal foramina. This ca. 3-4 cm long segment is in the vicinity of the parietal foramina.

III. Lambdoidal Asterica

LOCATION
The relatively straight part of the lambdoidal suture adjacent to asterion is scored.

IV. Zygomaticomaxillary

LOCATION
The entire length of the facial, or anterior, part of the zygomaticomaxillary suture is scored.

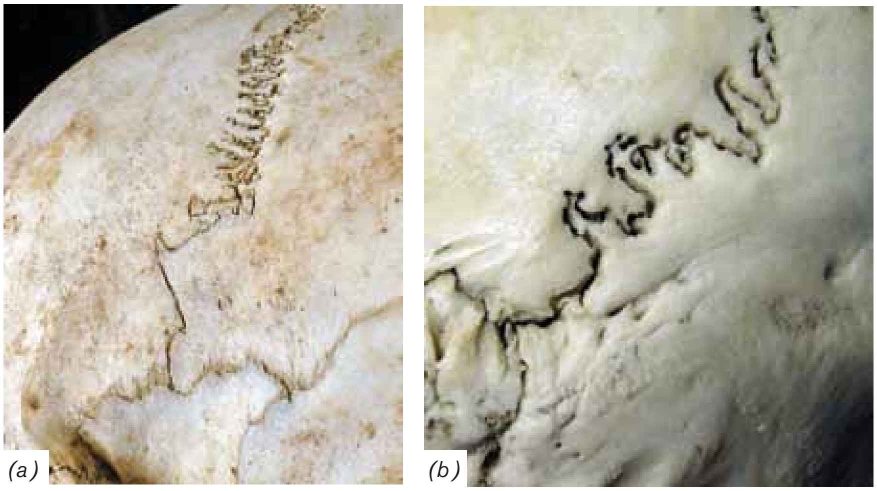

Figure 2.64. ***(a) Open*** *[1], Coronal Pterica (MC 6-69) and* ***(b) Open*** *[1], Lambdoidal Asterica (MC 7-76).*

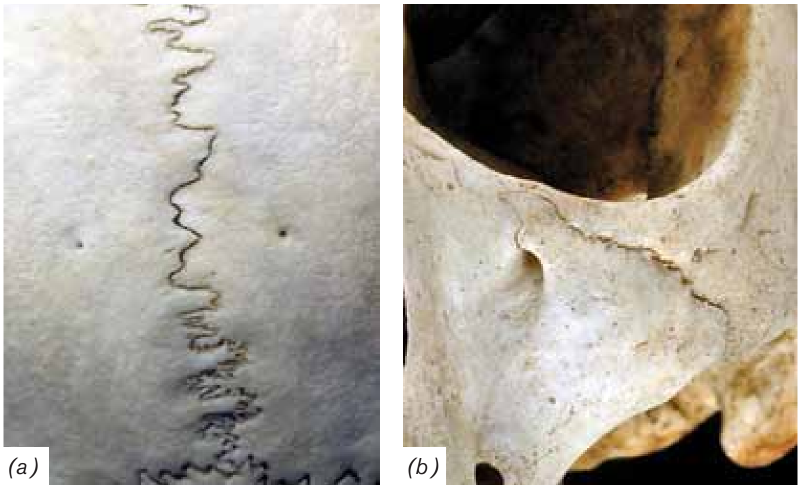

Figure 2.65. ***(a) Juxtaposed*** *[2], Sagittal Obelica (MC 7-76) and (b)* ***Juxtaposed*** *[2], Zygomaticomaxillary (MC 6-69).*

CHARACTERISTICS [STAGES]

1. Open
2. Juxtaposed
3. Partially obliterated
4. Punctuated
5. Obliterated

DEFINITIONS

1. *Open*: The suture is visible along its entire length, and there is a noticeable gap between the bones (Figure 2.64).

2. *Juxtaposed*: The suture is visible along its entire length, but the suture is narrow because the bones are tightly juxtaposed (Figure 2.65). If there are any bony bridges they are rare and small, sometimes with a trace of the original suture still evident.

3. *Partially obliterated*: The suture is partially obscured (Figure 2.66). There is no trace of the original suture in the bony bridges.

4. *Punctuated*: Only remnants of the suture are present (Figure 2.67). They appear as scattered small points or grooves each no more than two millimeters long.

5. *Obliterated*: There is no evidence of a suture (Figure 2.68).

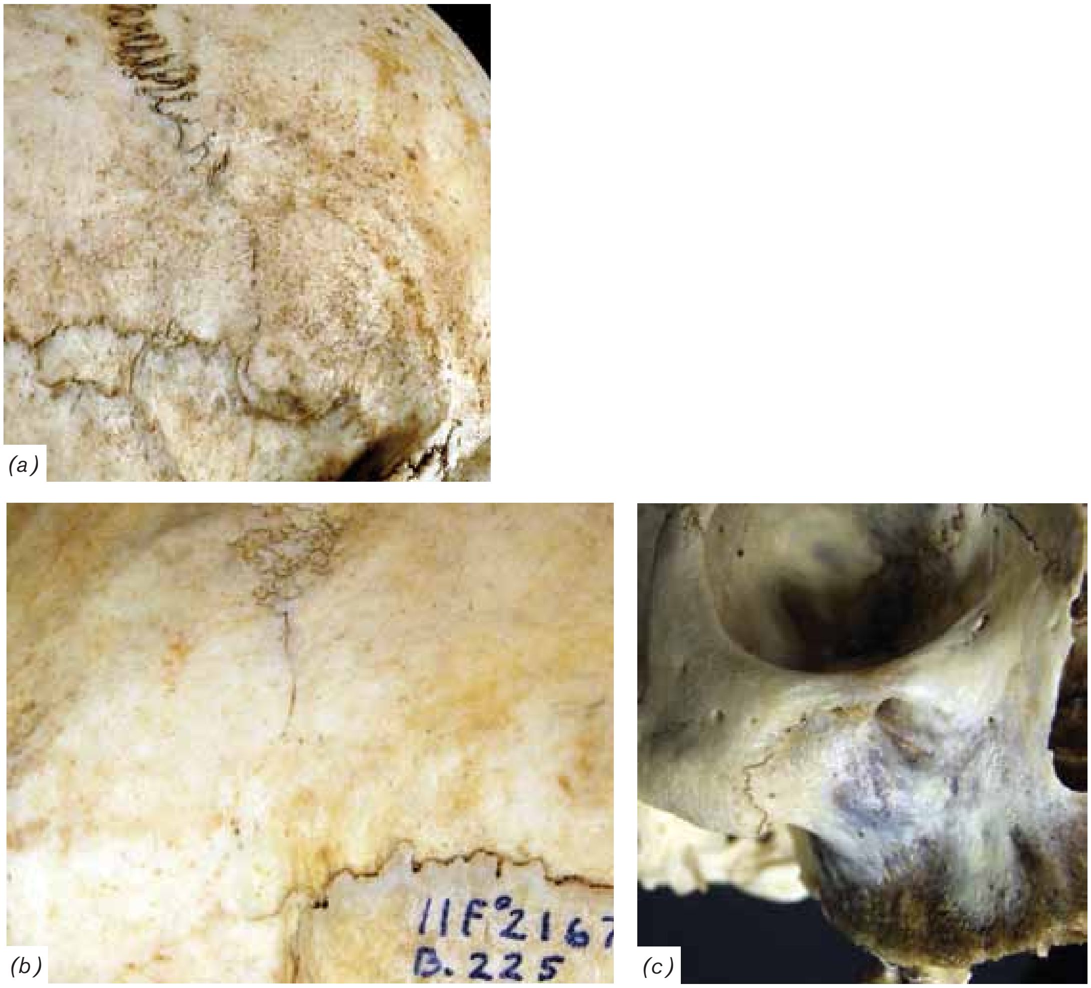

Figure 2.66. **(a) Partly obliterated** [3], Coronal Pterica (MC 6-69) and **(b) Partly obliterated** [3], Coronal Pterica (NF 225) and **(c) Partly obliterated** [3], Zygomaticomaxillary (MC 7-55).

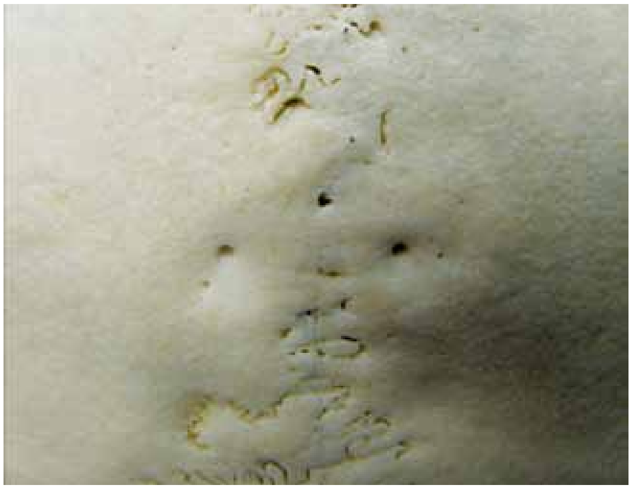

Figure 2.67. **Punctuated** [4], Sagittal Obelica (only Obelica, not segments on either side) (MC 7-55).

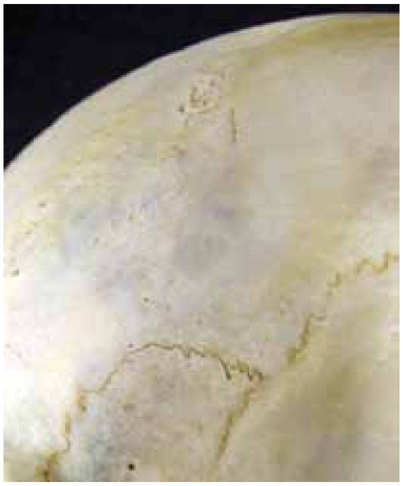

Figure 2.68. **Obliterated** [5], Coronal Pterica (MC 7-55).

V. Interpalatine (Median Palatine, Posterior Portion)

LOCATION

The suture located between the two opposing palatine bones is of interest (see Figure 2.69a). The *Open* [1] and *Juxtaposed* [2] distinction is not important because it is difficult to impossible to differentiate the two categories consistently. The *Juxtaposed* score [2] is eliminated for this suture to keep scoring consistent across all sutures and to reduce scoring error.

A small bony crest often forms along the midline of the palate that can make it difficult to record the extent of suture closure. In other specimens, the suture is barely visible in the depths of a deep and narrow groove. Both the ridge and groove make it hard or impossible to score the suture.

CHARACTERISTICS [STAGES]

1. Open (open and juxtaposed)
2. Partially obliterated
3. Punctuated
4. Obliterated

DEFINITIONS:

1. *Open (and juxtaposed)*: The suture is visible along its entire length, and there is a noticeable gap between the bones (Figure 2.69).
2. *Partially obliterated*: The suture is partially obscured (Figure 2.70). There is no trace of the original suture in the bony bridges.
3. *Punctuated*: Only remnants of the suture are present. They appear as scattered small points or grooves, each no more than 2 mm long.
4. *Obliterated*: There is no evidence of a suture.

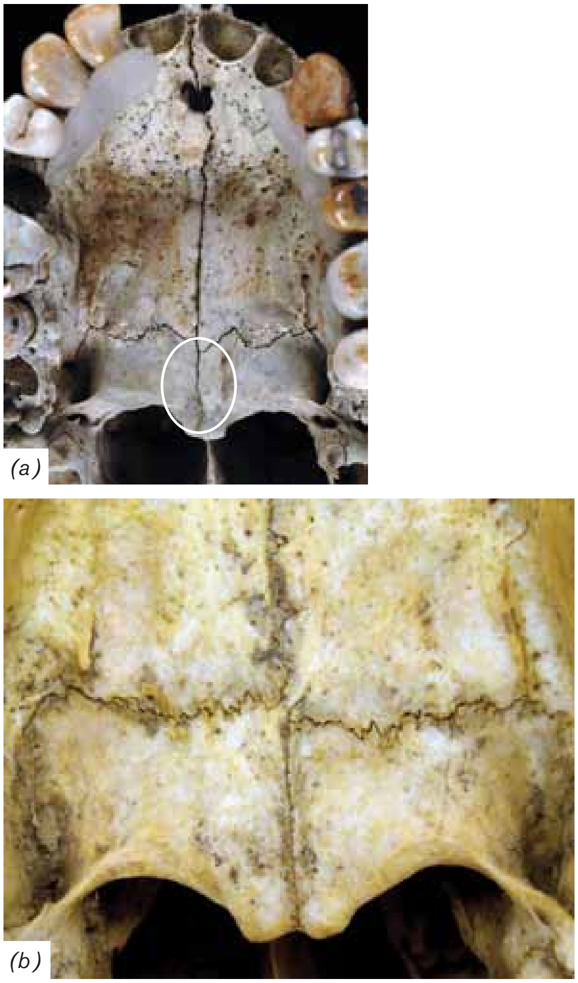

Figure 2.69.
***(a) Open & Juxtaposed** [1], Interpalatine (MC 6-69) and*
***(b) Open & Juxtaposed** [1], Interpalatine. These two stages are combined for the Interpalatine suture only (NF 229).*

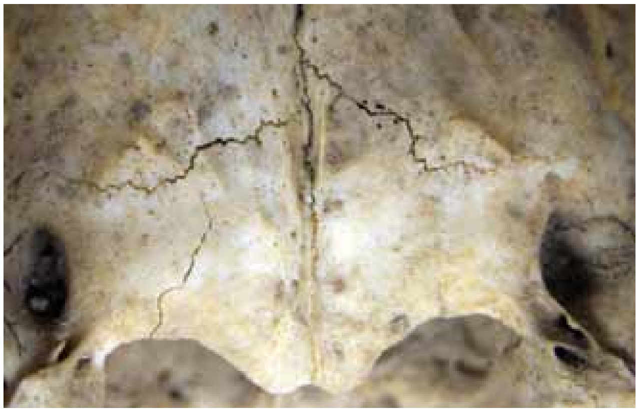

Figure 2.70. ***Partly obliterated** [3], Interpalatine (NF 225).*

SECTION 3 : SKELETAL MEASUREMENTS

Definitions of Cranial and Mandibular Landmarks

Table 3.1. Selected cranial landmarks.

1. Alveolon (alv)
2. Asterion (ast)
3. Basion (ba)
4. Bregma (b)
5. Condylion (cdl)
6. Dacryon (d)
7. Ectoconchion (ec)
8. Ectomolare (ecm)
9. Euryon (eu)
10. Frontomalare temporale (fmt)
11. Frontotemporale (ft)
12. Glabella (g)
13. Gnathion (gn)
14. Gonion (go)
15. Infradentale (id)
16. Lambda (l)
17. Mastoidale (ms)
18. Nasion (n)
19. Opisthocranion (op)
20. Opisthion (o)
21. Prosthion (pr)
22. Porion (po)
23. Radiculare (ra)
24. Zygion (zy)
25. Zygomaxillare anterior (zma)
26. Zygoorbitale (zo)

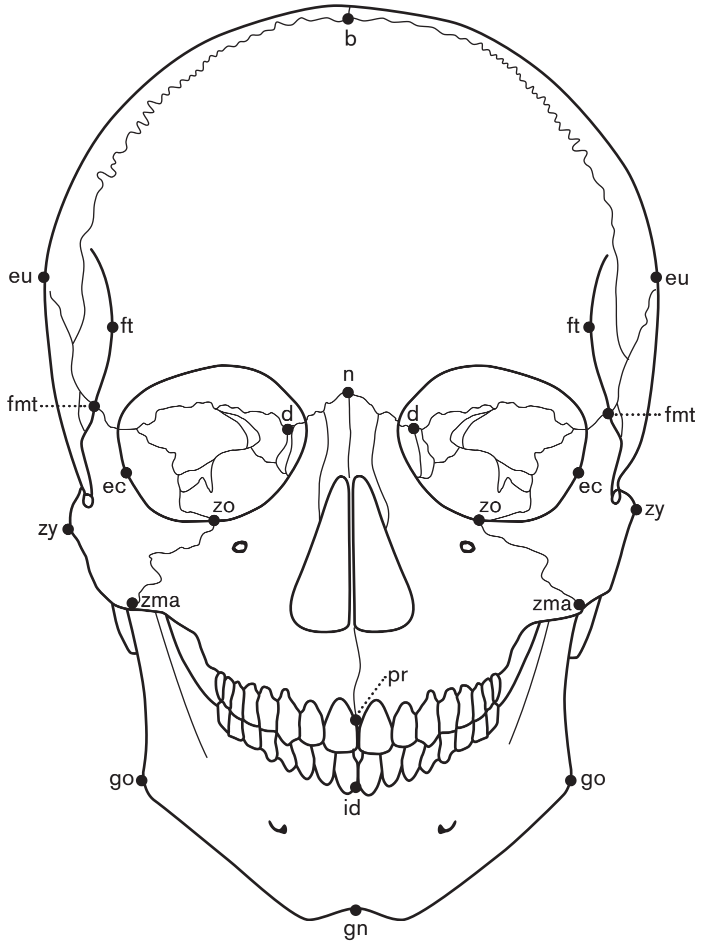

Figure 3.1. ***Selected anatomical landmarks of the skull*** *(anterior view).*

INTRODUCTION

1. **Alveolon (alv)**: The point where the mid-sagittal plane of the palate is intersected by a line connecting the posterior borders of the alveolar crests (Figure 3.2). To assist in locating the point, place a rubber band or wire against the posterior margins of the alveolar processes of the maxilla (Martin and Knussmann 1988:167).

2. **Asterion (ast)**: The point where the temporal, parietal, and occipital bones meet. (Figure 3.2). If the meeting point is occupied by a Wormian bone, extend the lambdoid suture onto its surface, and then extend the other two sutures (temporo-parietal, temporo-occipital) to the first line, finding asterion as the point midway between the intersections if these do not coincide. Use only the part of the last two sutures (ca. 1 cm) that is nearest the point when extending the lines. If the lambdoid (or other) suture is complex or composed of Wormian bones, trace a pencil line along the center of the area covered by the complexity, as well as can be done, to find the main axis of the suture. If the sutures gape at this point, leaving an open space, find asterion on the edge of the occipital bone. (Howells 1973: 166; Martin and Knussmann 1988: 164).

3. **Basion (ba)**: The point at which the anterior border of the foramen magnum is intersected by the mid-sagittal plane opposite nasion (na) (Figure 3.2). In rare cases the determination of the position of basion may be made difficult by a thickening of the anterior margin. (Howells 1973: 166).

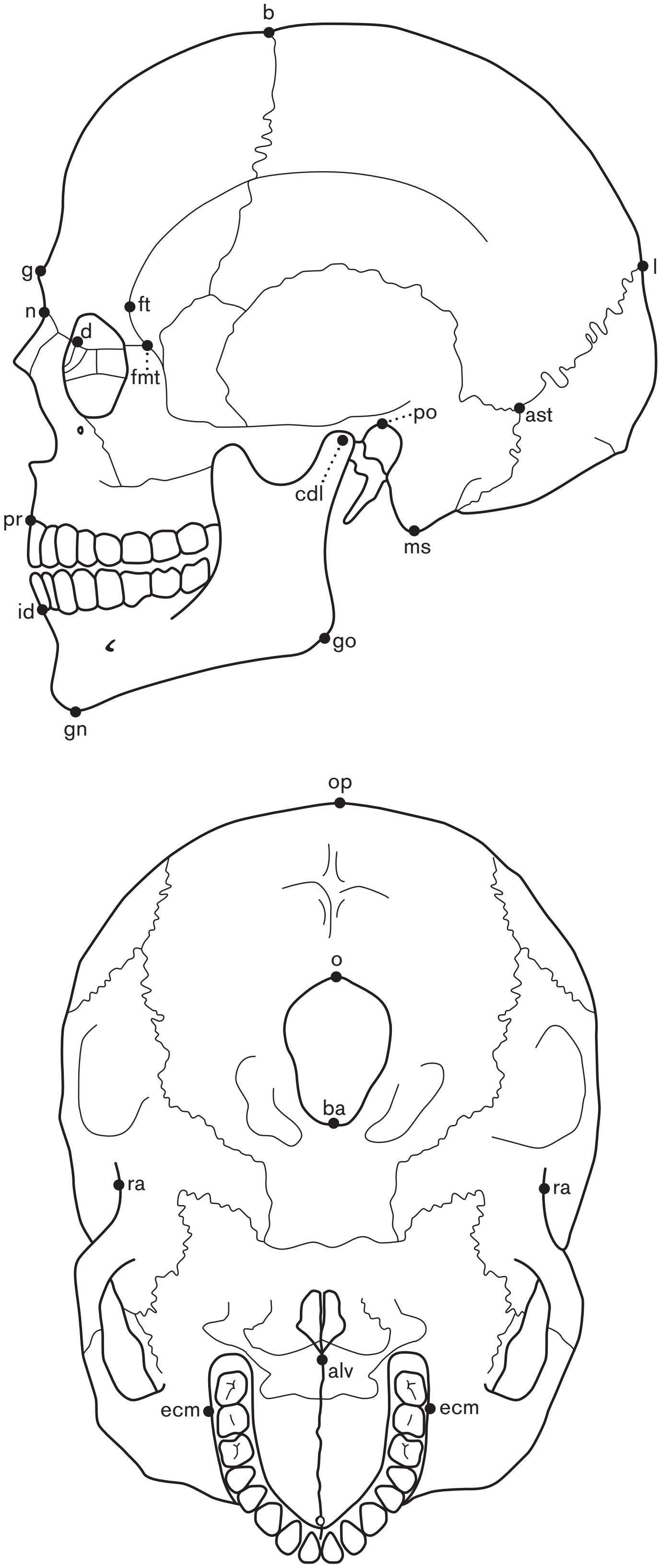

Figure 3.2. ***Selected anatomical landmarks of the skull*** *(lateral and basal view)*

4. **Bregma (b)**: The posterior border of the frontal bone in the mid-sagittal plane (Figure 3.1 and 3.2). Normally this is the meeting point of the coronal and sagittal sutures, on the frontal bone. The latter may diverge from the midline here, however, and should not then be followed (metopic sutures should be disregarded). In cases where the most anterior segment of the sagittal suture deflects to one side, the point of the junction of the two sutures must be projected. In cases of asymmetry of the coronal suture, the general course of the suture as a whole should be lightly drawn with a pencil, and the bregma established on this. The point should mark the limits of the frontal and parietal segments of the vault generally, not minor sutural variations. (Howells 1973: 167).

5. **Condylion (cdl)**: The most lateral points of the mandibular condyles (Figure 3.2) (Martin and Knussmann 1988: 168).

6. **Dacryon (d)**: Dacryon is located on the frontal bone. When the lacrimomaxillary suture is easily found, dacryon is the point on the *frontal bone* where the frontal, lacrimal and maxillary sutures meet (Figures 3.1 and 3.2). There is often a small foramen at this point. If the lacrimomaxillary suture cannot be found, dacryon is aligned with the apex of the lacrimal fossa. If the apex is inferior to the frontal bone, a line should be extended superiorly from the apex to the frontal, and the point should be marked on the frontal. (Howells 1973: 167; Martin and Knussmann 1988: 166).

7. **Ectoconchion (ec)**: The intersection of the most anterior edge of the lateral orbital border and a line parallel to the superior orbital border that bisects the orbit into two equal halves (Figure 3.1). To locate ectoconchion, trace a line along the anterior edge of the lateral orbital border with a pencil. Then hold a toothpick or thin straight instrument parallel to the superior orbital margin and move it down until the orbit is divided into two equal halves, making sure to keep the toothpick parallel to the superior margin. Mark the point (ectoconchion) where the toothpick intersects with the previously traced line. (Howells 1973: 168).

8. **Ectomolare (ecm)**: The most lateral point on the buccal surface of the alveolar margin (Figure 3.2). This point is generally positioned on the alveolar margin of the second maxillary molar (Martin and Knussmann 1988: 167).

9. **Euryon (eu)**: The most laterally positioned point on the side of the braincase (Figure 3.1). Euryon always falls on either the parietal bone or on the upper

portion of the temporal bone and may be determined only by measuring maximum cranial breadth. The area of the root of the zygomatic arch, the supra-mastoid crest, and the entire adjacent region above the external auditory meatus, which sometimes exhibit excessive symmetrical lateral expansion, should be avoided when determining the position of euryon (Martin and Knussmann 1988: 164).

10. **Frontomalare temporale (fmt)**: The most laterally positioned point on the fronto-malar suture (Figure 3.1 and 3.2) (Martin and Knussmann 1988: 167).

11. **Frontotemporale (ft)**: A point located generally forward and inward on the superior temporal line directly above the zygomatic process of the frontal bone (Figure 3.1 and 3.2). The right and left frontotemporale form the endpoints of the minimum frontal breadth measurement. (Martin and Knussmann 1988: 164).

12. **Glabella (g)**: The most anteriorly projecting point in the mid-sagittal plane at the lower margin of the frontal bone, which lies above the nasal root and between the superciliary arches (Figure 3.2). The point of glabella is depressed between the confining bony ridges, and is often delineated superiorly by a shallow gutter or a transversely running indentation on the surface of the frontal bone. (Martin and Knussmann 1988: 161).

13. **Gnathion (gn)**: The lowest point on the inferior margin of the mandibular body in the mid-sagittal plane (Figure 3.1 and 3.2). Frequently, gnathion is not the most inferiorly located point of the mandible, as the more laterally placed elements of the mandible may be extending far more inferiorly. This is particularly the case in mandibles with broad and square chin development (Martin and Knussmann 1988: 167).

14. **Gonion (go)**: The point on the mandible where the inferior margin of the mandibular corpus and the posterior margin of the ramus meet, i.e. the point on the mandibular angle which is directed most inferiorly, posteriorly, and laterally (Figure 3.1 and 3.2). If the mandibular angle is not pronounced, position the mandible with the angle facing upward. Gonion is positioned at the highest point of the curvature where the posterior margin of the ramus and the inferior border of the body meet. The point can also be found with the assistance of a mandibulometer by halving the mandibular angle and marking the point on the lateral border of the angle. When measuring the bigonial diameter the most lateral position of the angles should be chosen as measuring points (Martin and Knussmann 1988: 168).

15. **Infradentale (id)**: The point between the lower incisor teeth where the anterior margins of the alveolar processes are intersected by the mid-sagittal plane (Figure 3.1 and 3.2). (Martin and Knussmann 1988: 167).

16. **Lambda (l)**: The apex of the occipital bone at its junction with the parietals, in the midline (Figure 3.2). This is normally the meeting of the sagittal and lambdoid sutures, but must be placed in the midline.
    There is often an apical bone at the site, in which case lambda is to be found by extending the general curving course of each half of the lambdoid suture to their intersections with the midline; if these extensions do not meet the midline in a single point, lambda is halfway between such intersections.
    In occasional cases, the apex of the occipital makes an obvious forward excursion along the midline, away from the general course of the suture, and probably resulting from an apical bone joined to the occipital. The same procedure as immediately above is followed.
    The lambdoid suture itself may be very complex or composed largely of wormian bones. Trace a pencil line along the center of such an area on each side, to find lambda as above. (Howells 1973: 168; Martin and Knussmann 1988: 162).

17. **Mastoidale (ms)**: The most inferior point on the tip of the mastoid process (Figure 3.2). To determine the point, hold the skull base up with the lateral side facing you and mark the tip. (Martin and Knussmann 1988: 165).

18. **Nasion (n)**: The point of intersection of the naso-frontal suture and the mid-sagittal plane (Figure 3.1 and 3.2). As a general rule nasion is on the frontal bone. (Howells 1973: 169, Martin and Knussmann 1988: 165).

19. **Opisthocranion (op)**: The most distant point posteriorly from glabella on the occipital bone, located in the mid-sagittal plane (Figure 3.2). Opisthocranion almost always falls on the superior squama of the occipital bone, and only occasionally on the external occipital protuberance. Opisthocranion is established by obtaining the measurement of maximum cranial length (Martin and Knussmann 1988: 162).

20. **Opisthion (o)**: The point on the inner border of the posterior margin of the foramen magnum in the mid-sagittal plane (Figure 3.2). (Martin and Knussmann 1988: 163).

21. **Porion (po)**: The most superior point along the upper margin of the external acoustic meatus (Figure 3.2). (Martin and Knussmann 1988: 164).

22. **Prosthion (pr)**: The most anterior point on the alveolar border of the maxilla between the central incisors in the mid-sagittal plane (Figure 3.1 and 3.2) (Howells 1973: 169).

23. **Radiculare (ra)**: The point on the lateral aspect of the root of the zygomatic process at the deepest incurvature (Figure 3.2). (Martin and Knussmann 1988: 164).

24. **Zygion (zy)**: The most laterally positioned point on the zygomatic arches (Figure 3.1). The position of zygion is defined from the measurement of bizygomatic breadth (Martin and Knussmann 1988: 167).

25. **Zygomaxillare anterior (zma)**: The intersection of the zygomaxillary suture and the limit of the attachment of the masseter muscle, **on the facial surface** (Figure 3.1). If obliteration makes the facial part of the suture difficult to follow, inspection of its course, if present, on the internal surface of the arch may help. In very rare cases, and in association with an os japonicum, the suture runs more posteriorly, and over a centimeter lateral to the anterior end of the masseter attachment. Zygomaxillare anterior is then likely to be located beyond the angle of the malar; in such cases it is recommended that the point be placed on the facial surface, on the masseter limit, not more than 6-8 mm from the anterior end of the masseter area. (Howells 1973: 170).

26. **Zygoorbitale (zo)**: The intersection of the orbital margin and the zygomaxillary suture (Figure 3.1). Since the orbital border is usually softly rounded here, the point should be found midway between the facial and orbital surfaces. A small process or sliver of the malar may extend several millimeters medially from the rest of the bone just here, pushing the suture and point well inward along the orbital margin. As a convention, the point is never placed medial to the plane of the medial border of the infraorbital foramen. (Howells 1973: 170).

Cranial Measurements

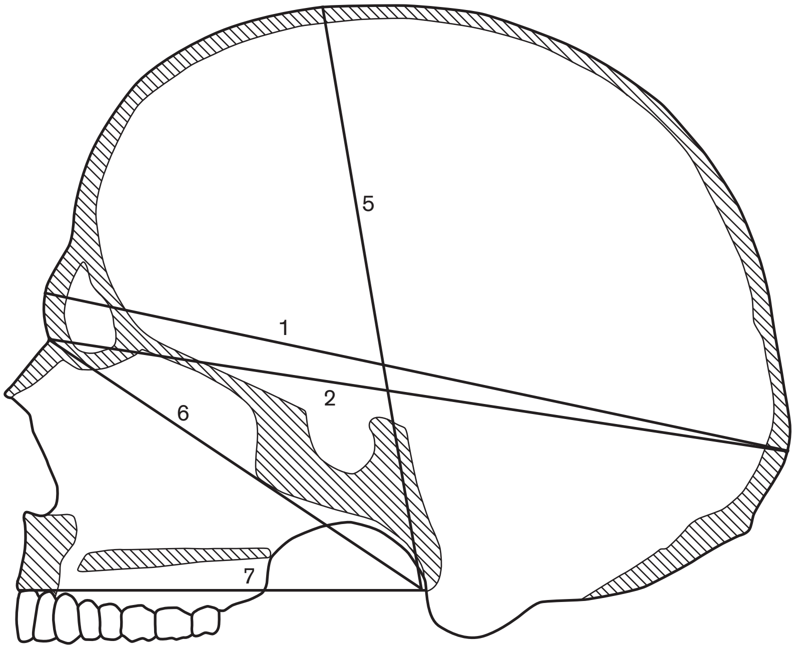

Figure 3.3. ***Selected cranial measurements in the mid-sagittal plane.***

1. **Maximum Cranial Length (g-op, GOL)**: The straight-line distance from glabella (g) to opisthocranion (op) in the mid-sagittal plane (Figure 3.3).

   Instrument: spreading caliper

   Comments: Set the skull on its base. Place one caliper point in the glabellar region in the midline and hold with fingers while the other caliper point is applied to the posterior portion of the skull in the mid-sagittal plane and moved up and down until the maximum length is obtained. On finding this, move the left point up and down slightly to make sure the reading is maximum. (Howells 1973: 170; Martin and Knussmann 1988: 168, #1).

2. **Nasio-occipital length (NOL)**: Maximum length in the mid-sagittal plane, measured from nasion (n) (Figure 3.3).

   Instrument: Spreading caliper

   Comments: With the skull in position as for glabello-occipital length, place the one caliper point at nasion and move the other point along the occiput in the midline for the maximum reading. (Howells 1973: 171).

3. **Maximum Cranial Breadth (eu-eu, XCB)**: The maximum width of the skull perpendicular to the mid-sagittal plane wherever it is located with the exception of the inferior temporal line and the immediate area surround the latter (i.e. the posterior roots of the zygomatic arches and supramastoid crest) (Figure 3.4).

   Instrument: spreading caliper

   Comments: Maximum Cranial Breadth is measured with the skull resting either on its base or on the occiput. The two measuring points (eurya) should lie in the same horizontal and frontal planes. Place the arms of the caliper at the same level while maintaining the hinge joint of the caliper in the mid-sagittal plane. Hold the ends of the caliper in each hand and apply to the lateral portions of the skull, making circular motions. Make sure you include areas below the squamosal suture, where the maximum is sometimes found. (Howells 1973:172; Hrdlicka 1920:14; Martin and Knussmann 1988: 170, #8).

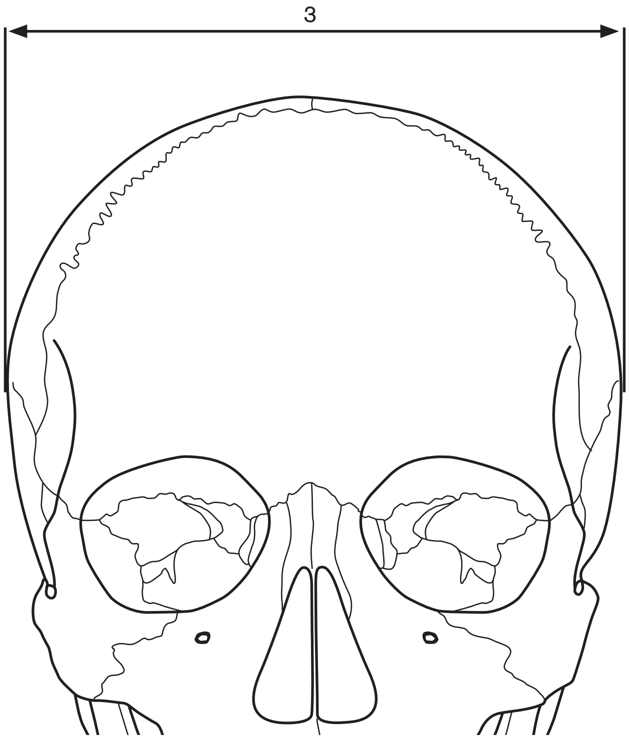

Figure 3.4. ***Maximum Cranial Breadth***

4. **Bizygomatic Breadth (zy-zy, ZYB)**: The maximum breadth across the zygomatic arches, wherever found, perpendicular to the mid-sagittal plane (Figure 3.5).

   Instrument: sliding or spreading caliper

   Comments: Place the skull on its occiput, base toward observer, and apply the blunt points of the caliper to the zygomatic arches and locate the maximum breadth perpendicular to the mid-sagittal plane. (Howells 1973: 173).

5. **Basion-Bregma Height (ba-b, BBH)**: The distance from basion (ba) to bregma (b) (Figure 3.3).

   Instrument: spreading caliper

   Comments: Place the skull on its occiput with the right side facing the observer. Apply the endpoint of one of the arms of the caliper at basion and support with fingers. Then apply the endpoint of the second arm of the caliper to bregma (Howells 1973: 172).

6. **Cranial Base Length (ba-n, BNL)**: The distance from nasion (n) to basion (ba) (Figure 3.3).

   Instrument: spreading caliper

   Comments: Rest the skull on the cranial vault and apply the endpoint of the one arm of caliper to nasion (n) while applying the other to basion (ba). (Howells 1973: 171-172, Martin and Knussmann 1988: 169, #5).

7. **Basion-Prosthion Length (ba-pr, BPL)**: The distance from basion (ba) to prosthion (pr) (Figure 3.3).

   Instrument: Spreading caliper or sliding caliper.

   Comments: Apply the fixed point of the sliding caliper or one tip of the spreading caliper to prosthion (pr). Then bring the movable point of the sliding caliper or the other tip of the spreading caliper to basion (ba) (Howells 1973: 174).

8. **Maxillo-Alveolar Breadth (ecm-ecm, MAB)**: The maximum breadth across the alveolar borders of the maxilla measured on the lateral surfaces at the location of the second maxillary molars (Figure 3.5).

   Instrument: spreading caliper

   Comments: The points of measurement (ecm) are not found on the alveolar processes when reactive alveolar bone is present; in these cases ecm is located on the bony segment superior to the second maxillary molars. Apply both arms of the caliper to the alveolar borders above the tooth row from an anterior position (Howells 1973:176; Martin and Knussmann 1988: 182 #61).

9. **Maxillo-Alveolar Length (pr-alv, MAL)**: The distance from prosthion (pr) to alveolon (alv) (Figure 3.5).

   Instrument: Spreading or sliding caliper. A sliding caliper is only useful if the central incisors have been lost.

   Comments: Place the skull so the base faces up. Apply a thin wire, rubber band, or other similar implement to the posterior borders of the alveolar arch and measure the distance from prosthion (pr) to the middle of the wire/band in the mid-sagittal plane (alv). (Martin and Knussmann 1988: 182 #60).

10. **Biauricular Breadth (ra-ra, AUB)**: The least exterior breadth across the roots of the zygomatic processes (Figure 3.5).

    Instrument: sliding caliper

    Comments: With the skull resting on the occiput and with the base toward the observer measure to the outside of the roots of the zygomatic processes at their deepest incurvature, generally slightly anterior to the external auditory meatus, with the sharp points of the caliper (Howells 1973:173; Martin and Knussmann 1988: 170, #11b).

11. **Nasion-Prosthion Height (n-pr, NPH)**: The distance from nasion (n) to prosthion (pr) (Figure 3.6).

    Instrument: sliding caliper

    Comments: Place the fixed point of the caliper on nasion and apply the movable point to prosthion. If the alveolar process exhibits slight resorption or erosion at the point of prosthion,

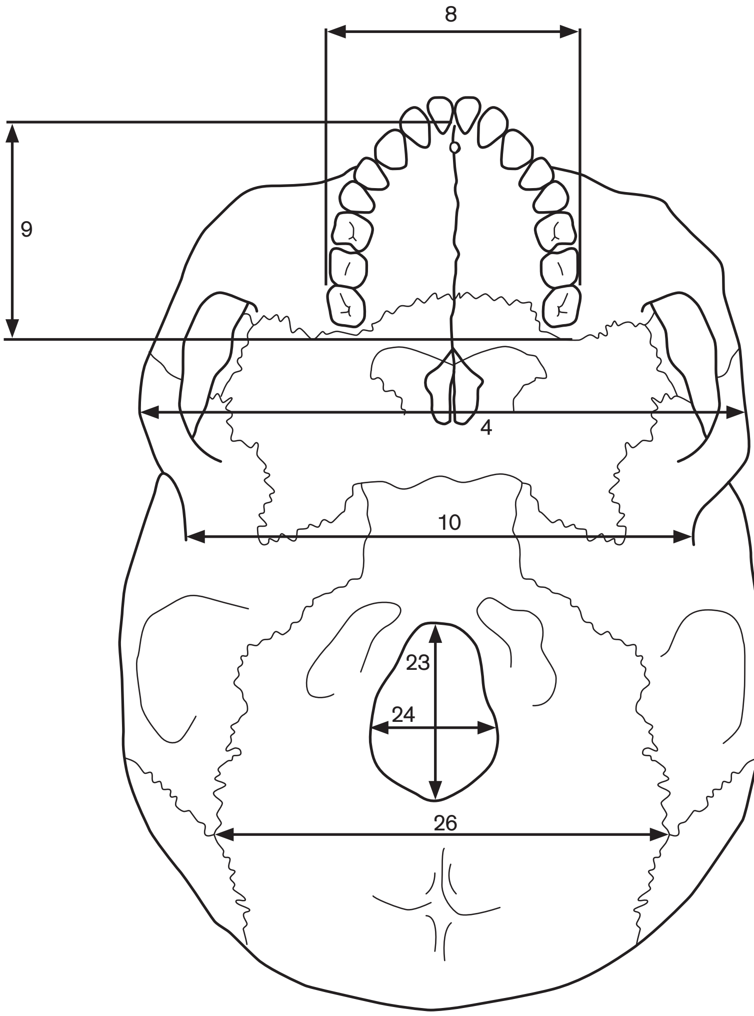

Figure 3.5. ***Measurements taken with the skull resting on bregma with the base facing up.***

the projection of the process may be estimated when the alveolar process of the lateral incisors is still intact. When resorption or erosion is more pronounced this measurement should not be taken (Howells 1973: 174).

12. **Minimum Frontal Breadth (ft-ft, WFB)**: The distance between the right and left frontotemporale (Figure 3.6).

    Instrument: sliding caliper

    Comments: Place the skull on its base. The two endpoints of the caliper are placed on the temporal ridges at the two frontotemporale. When taking this measurement make certain that the least distance between both temporal lines on the frontal bone is recorded (Hrdlicka 1920: 15; Martin and Knussmann 1988: 170, #9).

13. **Upper Facial Breadth (fmt-fmt)**: The distance between the right and left frontomalare temporale (Figure 3.6).

    Instrument: sliding caliper

    Comments: The measurement is taken between the two external points on the frontomalar suture (Martin and Knussmann 1988: 179, #43).

14. **Nasal Height (NLH)**: The average height from nasion (n) to the lowest point on the border of the nasal aperture on either side (Figure 3.6).

    Instrument: sliding caliper

    Comments: Place the skull on its occiput, base to the right, and measure the distance from nasion to the inferior border of the nasal aperture on each side and take the average of these two measurements to the nearest whole millimeter. The lower border of the aperture is well defined in most populations. It is not always the most anterior edge, but the beginning of the actual floor of the nasal cavity. It is the hinder border, not the forward border, of any prenasal gutter or fossa. (Howells 1973: 175).

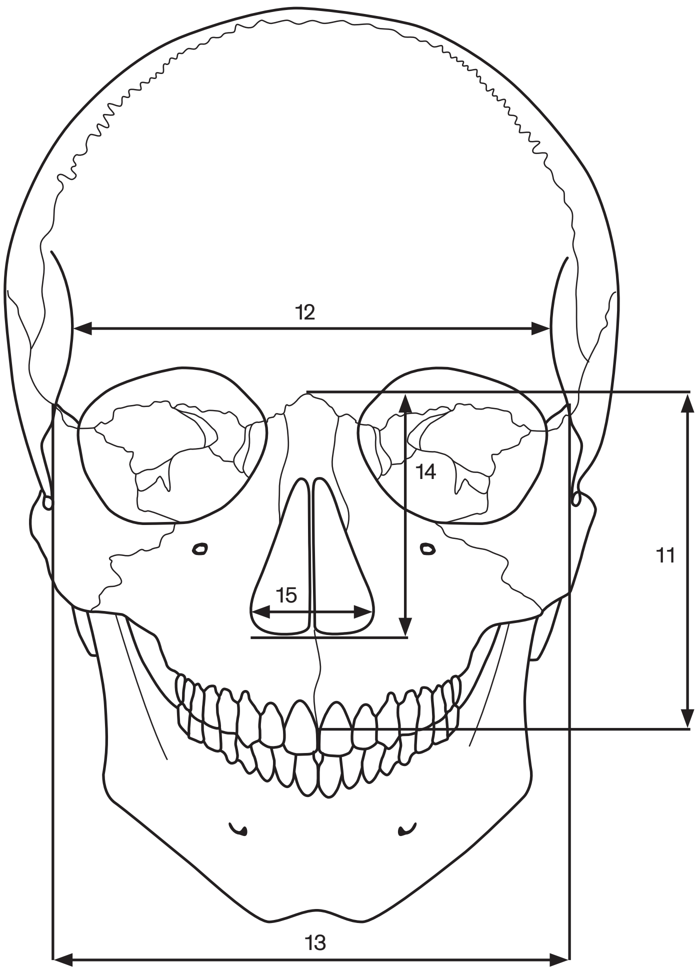

Figure 3.6. ***Selected cranial measurements*** *(anterior view).*

15. **Nasal Breadth (NLB)**: The maximum breadth of the nasal aperture (Figure 3.6).

    Instrument: sliding caliper

    Comments: Carefully place the points of the instrument on the sharp lateral margins of the nasal aperture at its most lateral curvature; this is not an inside measurement. The measurement is perpendicular to the mid-sagittal plane and recorded to the nearest millimeter (Howells 1973: 176; Martin and Knussmann 1988: 181 #54).

16. **Orbital Breadth (d-ec, 0BB)**: The distance from dacryon (d) to ectoconchion (ec) (Figure 3.7).

    Instrument: sliding caliper

    Comments: The inside jaws of the calipers are useful for this measurement. For standardization and practical reasons measure the left orbit. Measure the right orbit if the left orbit is damaged and record the side measured on the recording sheet. (Howells 1973: 175; Martin and Knussmann l988: 181, #51a).

17. **Orbital Height (OBH)**: The distance between the superior and inferior orbital margins perpendicular to orbital breadth and bisecting the orbit into equal medial and lateral halves. (Figure 3.7).

    Instrument: sliding caliper

    Comments: Orbital height is measured perpendicular to orbital breadth. Any notches or depressions on either superior or inferior borders should be avoided; if there is deep notching, move the caliper medially slightly. The inside jaws of the calipers are useful for this measurement. (Howells 1973: 175; Martin and Knussmann 1988: 181, #52).

18. **Biorbital Breadth (ec-ec, EKB)**: The distance from left to right ectoconchion (ec) (Figure 3.7). (Howells 1973:178)

    Instrument: sliding caliper

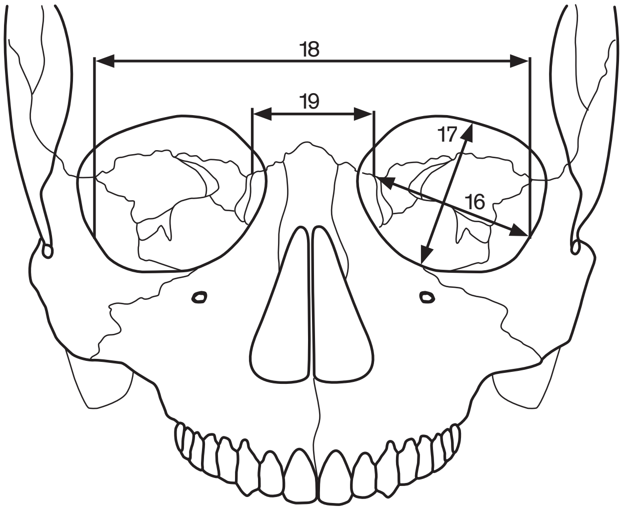

Figure 3.7. ***Selected measurements of the orbital region.***

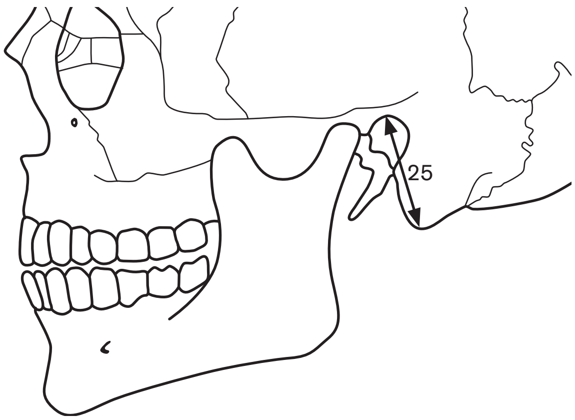

Figure 3.8. ***Mastoid height.***

19. **Interorbital Breadth (d-d, DKB)**: The distance between right and left dacryon (Figure 3.7). (Howells 1973: 178)

    Instrument: sliding caliper

20. **Frontal Chord (n-b, FRC)**: The distance from nasion (n) to bregma (b) taken in the mid-sagittal plane (Figure 3.9).

    Instrument: sliding caliper

    Comments: Place the tips of the instrument on the bone surface or at the level of the bone surface and not in a suture or other depression (Howells 1973:181; Martin and Knussmann 1988: 174, #29).

21. **Parietal Chord (b-l, PAC)**: The distance from bregma (b) to lambda (l) taken in the mid-sagittal plane (Figure 3.9).

    Instrument: sliding caliper

    Comments: Place the tips of the instrument on the bone surface or at the level of the bone surface and not in a suture or other depression (Howells 1973:182; Martin and Knussmann 1988: 175, #30).

22. **Occipital Chord (l-o, OCC)**: The distance from lambda (l) to opisthion (o) taken in the mid-sagittal plane (Figure 3.9). (Howells 1973:182; Martin and Knussmann 1988: 175 #31)

    Instrument: sliding caliper

23. **Foramen Magnum Length (FOL)**: The mid-sagittal distance from the most anterior point on the foramen magnum margin to opisthion (o) (Figure 3.5).

    Instrument: sliding caliper

    Comments: Measure with the skull base up, using the inside jaws of the calipers. (Martin and Knussmann 1988: 169 #7).

24. **Foramen Magnum Breadth (FOB)**: The distance between the lateral margins of the foramen magnum at the point of greatest lateral curvature (Figure 3.5).

    Instrument: sliding caliper

    Comments: Measure with the skull base up using the inside jaws of the calipers.
    (Martin and Knussmann 1988: 171, #16).

25. **Mastoid Height (MDH)**: The direct distance between porion and mastoidale (Figure 3.8).

    Instrument: sliding caliper

    Comments: Note that both right and left sides are measured. Place the fixed arm of the caliper on porion and move the movable arm until it touches mastoidale. This may be most easily accomplished by holding the calipers in a coronal plane. Howells (1973: 176) and Martin and Knussmann (1988: 172, #19a) define this measurement similarly, though their measurement technique differs from that described

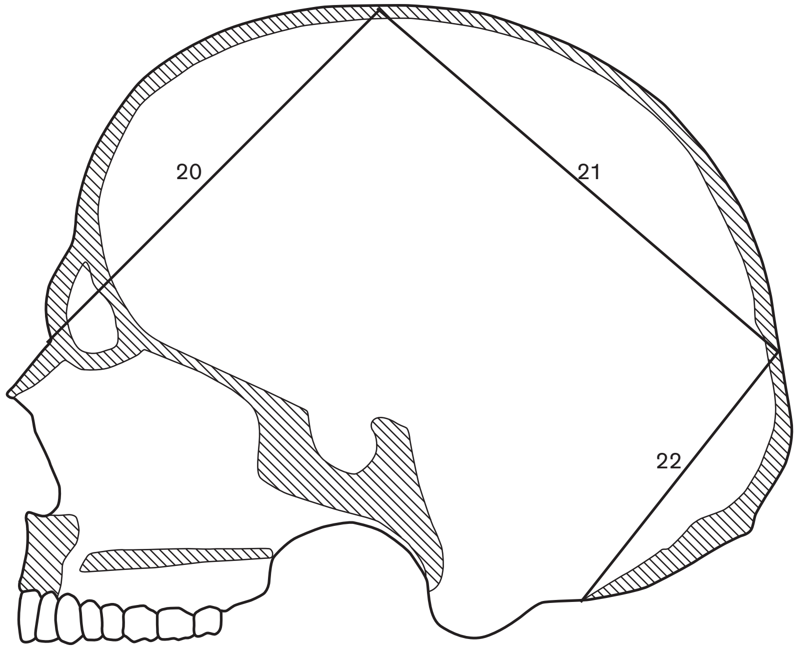

Figure 3.9. ***Frontal, Parietal, and Occipital Chord measurements***

here. When only one value is needed, average the right and left measurements. If the discrepancy between the sides is more than 2mm repeat as a check.

26. **Biasterionic Breadth (ast-ast, ASB)**: Straight-line distance from left to right asterion (as) (Figure 3.5).

    Instrument: Sliding caliper

    Comments: With the skull resting so that the occiput is facing the observer, measure the distance using the sharp points of the calipers. (Howells 1973: 174; Martin and Knussmann 1988: 171, #12).

27. **Bimaxillary breadth (zma-zma, ZMB)**: The breadth across the maxillae, from the left to right zygomaxillare anterior (zma) (Figure 3.10).

    Instrument: sliding caliper

    Comments: The endpoints of the measurement are located on the facial surface and not on the inferior aspect of the zygomaxillary suture. (Howells 1973: 177).

28. **Zygoorbitale breadth (zo-zo, ZOB)**: The distance between right and left zygoorbitale (zo) (Figure 3.10).

    Instrument: sliding caliper

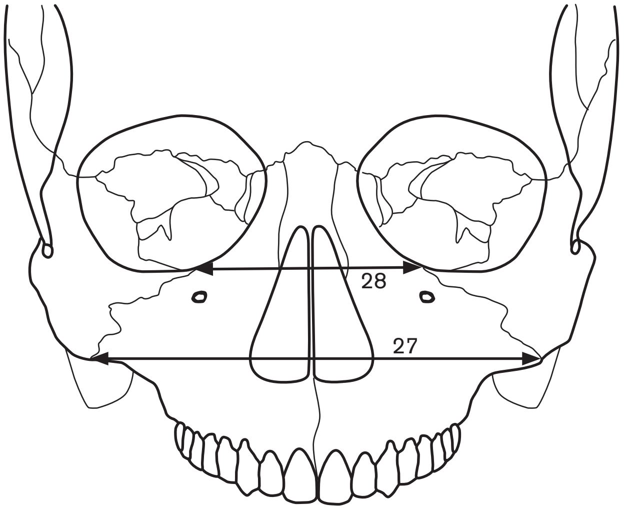

Figure 3.10. ***Bimaxillary breadth and zygoorbitale breadth.***

Mandibular Measurements

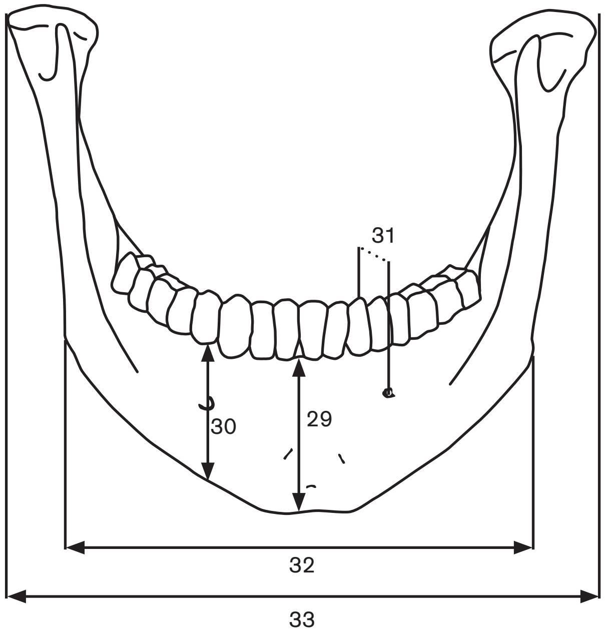

Figure 3.11. ***Selected measurements of the mandible*** *(anterior view).*

29. **Chin Height (id-gn)**: The distance from infradentale (id) to gnathion (gn) (Figure 3.11).

    Instrument: sliding caliper

    Comments: When the mandibular alveolar process is damaged between the central incisors, estimates of this measurement are recorded by using the intact alveolar process at the position of the lateral incisors. Any estimate or adjustment should be indicated on the recording form. In mandibles where the alveolar process exhibit pronounced erosion or resorption, this measurement is not taken (Martin and Knussmann 1988: 183, #69).

30. **Height of the Mandibular Body**: The distance from the alveolar process to the inferior border of the mandible at the level of the mental foramen (Figure 3.11 and 3.12). (Martin and Knussmann 1988: 183, #69.1).

31. **Breadth of Mandibular Body**: The maximum breadth measured at the level of the mental foramen perpendicular to the long axis of the mandibular body (Figure 3.11). (Martin and Knussmann 1988: 183, #69.3).

    Instrument: sliding caliper

32. **Bigonial Breadth (go-go)**: The distance between the right and left gonion (go) (Figure 3.11).

    Instrument: sliding caliper

    Comments: Apply the blunt points of the caliper arms to the most prominent external points at the mandibular angles (Martin and Knussmann 1988: 182, #66).

33. **Bicondylar Breadth (cdl-cdl)**: The distance between the most lateral points on the mandibular condyles (cdl) (Figure 3.11).

    Instrument: sliding caliper

    Comments: Place the blunt point of the fixed end of the instrument against the right condyle and apply the movable end to the left condyle (Martin and Knussmann 1988: 182, #65).

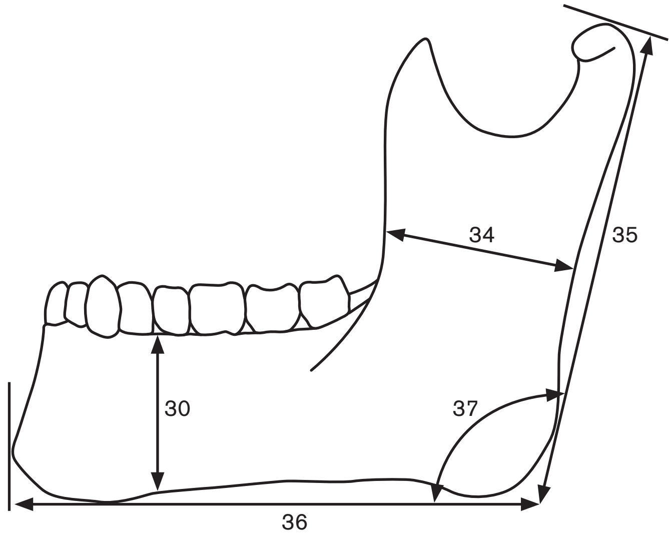

Figure 3.12. ***Selected measurements of the mandible*** *(lateral view).*

34. **Minimum Ramus Breadth**: The minimum breadth of the mandibular ramus measured perpendicular to the height of the ramus (Figure 3.12).

    Instrument: sliding caliper

    Comments: The measurement is taken at the waist of the ramus (the point where the distance between the anterior and posterior margins of the ramus is the smallest). (Martin and Knussmann 1988: 183, #71a).

35. **Maximum Ramus Height**: The distance from gonion (go) to the highest point on the mandibular condyle (Figure 3.12).

    Instrument: mandibulometer

    Comments: Apply movable board of the mandibulometer to the posterior borders of the mandibular rami and the fixed board against the most anterior point of the chin (Martin and Knussmann 1988: 183, #70).

36. **Mandibular Length**: The distance from the anterior margin of the chin to the midpoint of a straight line extending from the posterior border of the right and left mandibular angles (Figure 3.12).

    Instrument: mandibulometer

    Comments: (Martin and Knussmann 1988: 182, #68).

37. **Mandibular Angle**: The angle formed by inferior border of the corpus and the posterior border of the ramus (Figure 3.12).

    Instrument: mandibulometer

    Comment: In case of "rocker jaws", the bone is held in place by pressing down on the second molars (Martin and Knussmann 1988: 185, #79).

Postcranial Measurements

NOTE: When recording postcranial elements be sure to indicate if epiphyses are absent.

NOTE: Do not include lipping in measurements of articular surfaces. If original articular surface cannot be approximated, do not take the measurement.

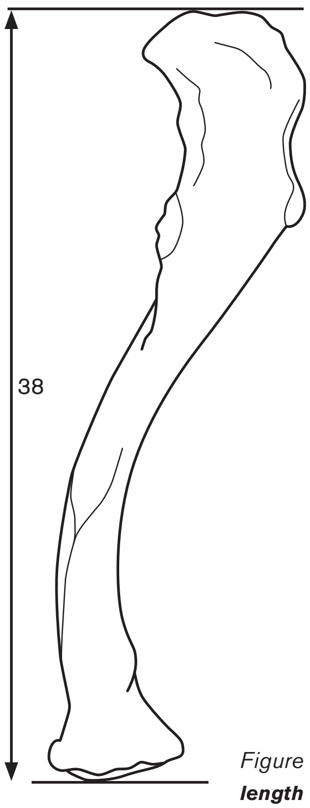

Figure 3.13. ***Maximim length of the clavicle.***

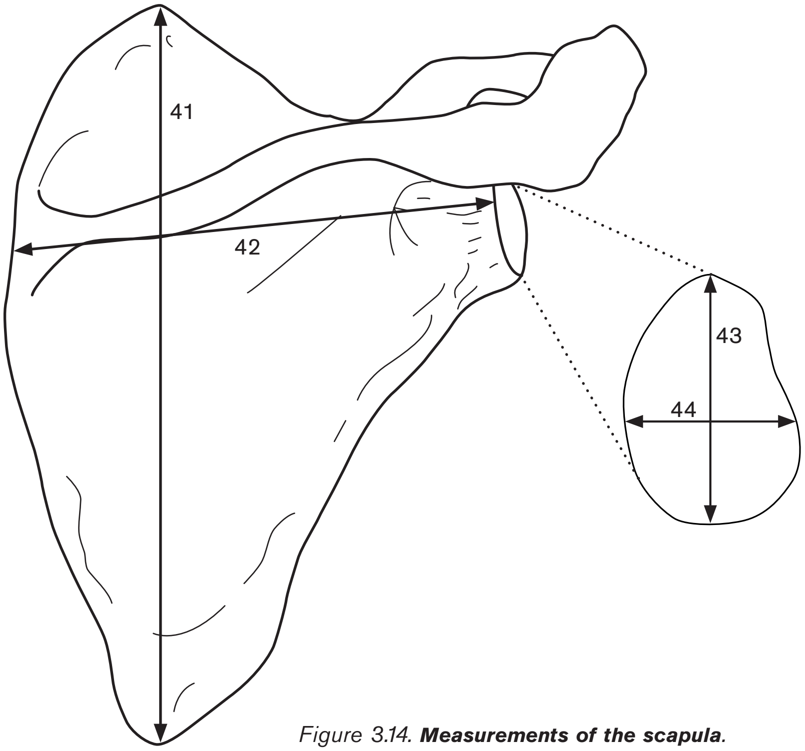

Figure 3.14. ***Measurements of the scapula.***

38. **Maximum Length of the Clavicle**: The maximum distance between the most extreme ends of the clavicle (Figure 3.13).

    Instrument: osteometric board

    Comment: Place the bone on the osteometric board and place the sternal end of the clavicle against the vertical end board. Press the movable upright against the acromial end and move the bone up, down and sideways until the maximum length is obtained (Martin and Knussmann 1988: 197, #1).

39. **Maximum Diameter of the Clavicle at Midshaft**: The maximum diameter of the bone measured at midshaft.

    Instrument: sliding caliper

    Comment: Determine the midpoint of the diaphysis on the osteometric board and mark it with a pencil. Place the bone between the two arms of the caliper and rotate the bone until the maximum diameter is obtained.

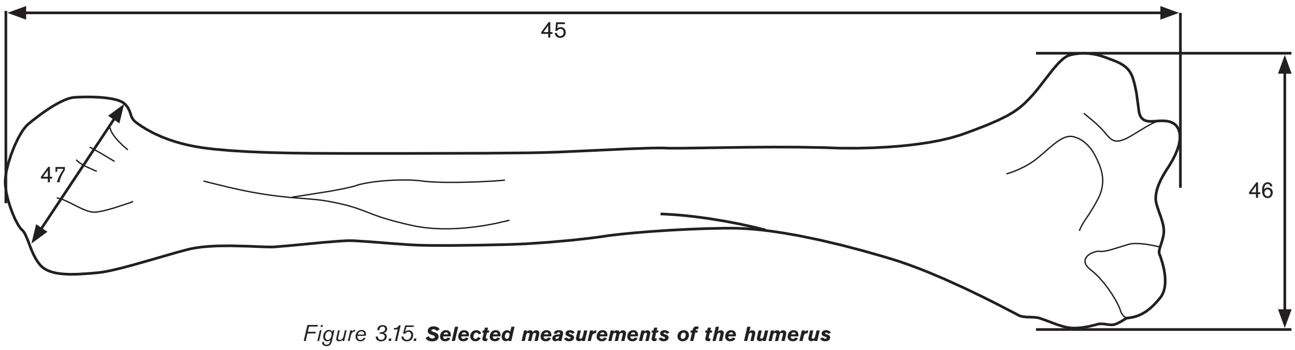

Figure 3.15. ***Selected measurements of the humerus***

40. **Minimum Diameter of the Clavicle at Midshaft**: The minimum diameter of the bone measured at midshaft.

    Instrument: sliding caliper

    Comment: Determine the midpoint of the diaphysis on the osteometric board and mark it with a pencil. Place the bone between the two arms of the caliper and rotate the bone until the minimum diameter is obtained.

41. **Height of the Scapula**: The distance from the most superior point of the cranial angle to the most interior point on the caudal angle (Figure 3.14).

    Instrument: sliding caliper or osteometric board

    Comment: (Martin and Knussmann 1988:197 #1).

42. **Breadth of the Scapula**: The distance from the midpoint on the dorsal border of the glenoid fossa to midway between the two ridges of the scapular spine on the vertebral border (Figure 3.14).

    Instrument: sliding or spreading caliper

    Comment: Project a line through the obtuse angle of a triangle formed by the vertebral border and the two ridges of the spine, dividing it into two equal halves. The medial measuring point is located where this line intersects the vertebral border (Hrdlicka 1920: 131).

43. **Glenoid Cavity Breadth**: Maximum distance from the ventral to dorsal margins of the glenoid cavity, taken perpendicular to glenoid cavity height. In cases of severe lipping, this measurement should not be taken (Figure 3.14). (Martin and Knussmann 1988: 198, #13).

    Instrument: sliding caliper

44. **Glenoid Cavity Height**: The distance from the most superiorly located point on the margin of the glenoid cavity to the most inferiorly located point on the margin, taken perpendicular to glenoid cavity breadth. In cases of severe lipping, this measurement should not be taken (Figure 3.14). (Martin and Knussmann 1988: 198, #12).

    Instrument: sliding caliper

45. **Maximum Length of the Humerus**: The distance from the most superior point on the head of the humerus to the most inferior point on the trochlea (Figure 3.15).

    Instrument: osteometric board

    Comment: Place the humerus on the osteometric board so that its long axis parallels the instrument. Place the head of the humerus against the vertical end board and press the movable upright against the trochlea. Move the bone up, down and sideways to determine the maximum distance (Hrdlicka 1920:126).

46. **Epicondylar Breadth of the Humerus**: The distance from the most laterally protruding point on the lateral epicondyle to the corresponding projection on the medial epicondyle (Figure 3.15). (Martin and Knussmann 1988: 199, #4).

    Instrument: osteometric board or sliding calipers

47. **Maximum Vertical Diameter of the Head of the Humerus**: The distance between the most superior and inferior points on the border of the articular surface (Figure 3.15).

    Instrument: sliding caliper

    Comment: Do not include arthritic lipping which may be present on the perimeter of the joint surface. This diameter is not necessarily the maximum head diameter (Martin and Knussmann 1988: 200, #10).

48. **Maximum Diameter of the Humerus at Midshaft**: The maximum diameter of the humeral shaft at midshaft.

    Instrument: sliding caliper

    Comment: Determine the midpoint of the diaphysis on the

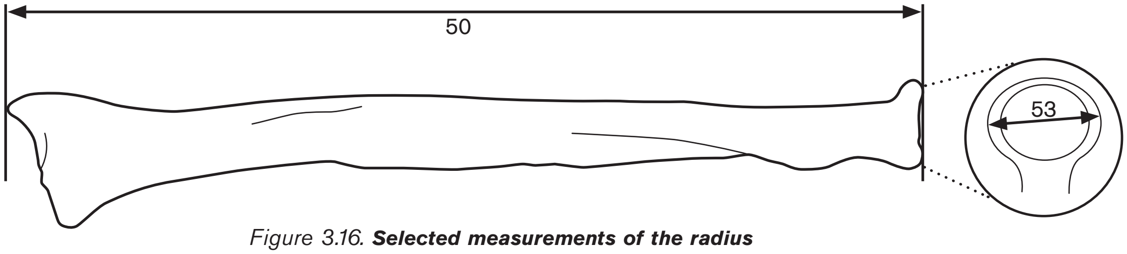

Figure 3.16. ***Selected measurements of the radius***

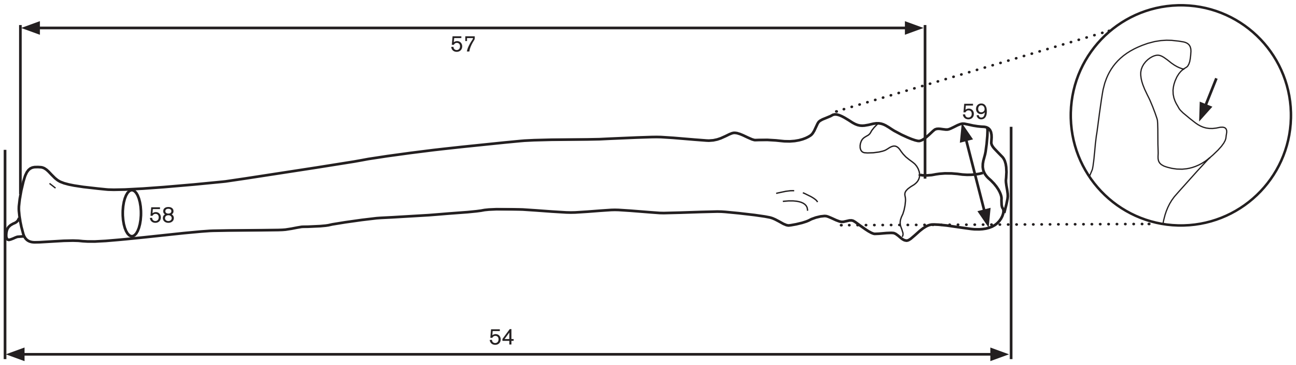

Figure 3.17. ***Selected measurements of the ulna***

osteometric board and mark with a pencil. Using sliding calipers to measure with one hand, rotate the bone with the other hand until the maximum diameter is obtained. (Martin and Knussmann 1988: 199, #5).

49. **Minimum Diameter of the Humerus at Midshaft**: The minimum diameter of the humeral shaft at midshaft.

    Instrument: sliding caliper

    Comment: Determine the midpoint of the diaphysis on the osteometric board and mark with a pencil. Using sliding calipers to measure with one hand, rotate the bone with the other hand until the minimum diameter is obtained. (Martin and Knussmann 1988: 199, #6).

50. **Maximum Length of the Radius**: The distance from the most proximally positioned point on the head of the radius to the tip of the styloid process without regard to the long axis of the bone (Figure 3.16).

    Instrument: osteometric board

    Comment: Place the proximal end against the vertical upright of the osteometric board and press the movable upright against the distal end. Move the bone up, down and sideways to obtain the maximum length (Martin and Knussmann 1988: 201, #1; Hrdlicka 1920: 127).

51. **Maximum Diameter of the Radius at Midshaft**: The maximum diameter of the radial shaft taken at midshaft.

    Instrument: sliding caliper

    Comment: Mark the midshaft of the bone with a pencil. Using sliding calipers to measure with one hand, rotate the bone with the other hand until the maximum diameter is obtained.

52. **Minimum Diameter of the Radius at Midshaft**: The minimum diameter of the radial shaft taken at midshaft.

    Instrument: sliding caliper

    Comment: Mark the midshaft of the bone with a pencil. Using sliding calipers to measure with one hand, rotate the bone with the other hand until the minimum diameter is obtained.

53. **Maximum Diameter of the Radial Head**: The maximum diameter of the radial head measured on the margin of the head that articulates with the ulna. The bone is rotated until the maximum distance is obtained (Figure 3.16) (Montagu 1960: 68).

    Instrument: sliding caliper

54. **Maximum Length of the Ulna**: The distance between the most proximal point on the olecranon and the most distal point on the styloid process (Figure 3.16).

    Instrument: osteometric board

    Comment: Place the proximal end of the ulna against the vertical end board. Press the movable upright against the

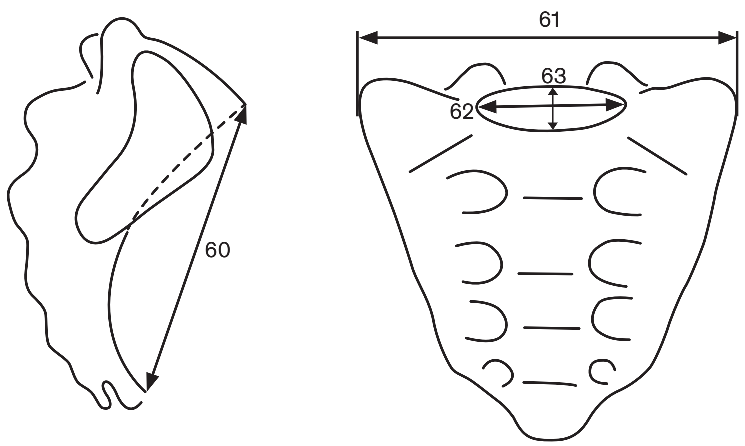

Figure 3.18. ***Selected measurements of the sacrum.***

distal end while moving the bone up, down and sideways to obtain the maximum length (Hrdlicka 1920; 127; Martin and Knussmann 1988: 204, #1).

55. **Maximum Midshaft Diameter of the Ulna**: The maximum diameter of the diaphysis at midshaft.

    Instrument: sliding caliper

    Comment: Mark the midshaft of the bone with a pencil. Using sliding calipers to measure with one hand, rotate the bone with the other hand until the maximum diameter is obtained.

56. **Minimum Midshaft Diameter of the Ulna**: The minimum diameter of the diaphysis at midshaft.

    Instrument: sliding caliper

    Comment: Mark the midshaft of the bone with a pencil. Using sliding calipers to measure with one hand, rotate the bone with the other hand until the minimum diameter is obtained.

57. **Physiological Length of the Ulna**: The distance between the deepest point on the articular surface of the coronoid process on the guiding ridge and the most inferior point on the distal articular surface of the ulna (Figure 3.17).

    Instrument: spreading caliper

    Comment: Do not include the styloid process or the groove between the styloid process and the distal articular surface (Martin and Knussmann 1988: 204, #2).

58. **Minimum Circumference of the Ulna**: The least circumference near the distal end of the bone (Figure 3.17).

    Instrument: tape

    Comment: (Martin and Knussmann 1988: 204, #3).

59. **Olecranon Breadth**: The maximum breadth of the olecranon process, taken perpendicular to the longitudinal axis of the semilunar notch (Figure 3.17). (Martin and Knussmann 1988: 206, #6)

    Instrument: sliding caliper

NOTE: Record the number of segments composing the sacrum and disregarding the coccyx.

60. **Anterior Height of the Sacrum**: The distance from the point on the promontory in the mid-sagittal plane to the corresponding point on the anterior border of the distal tip of the sacrum (Figure 3.18).

    Instrument: sliding caliper

    Comment: Place the pointed tips of the caliper on the promontory and the anterior inferior border of the fifth sacral vertebra. The measurement is taken in the mid-sagittal plane. If a sacrum exhibits more than five segments, measure to the bottom segment and indicate the number of segments on the recording form. **If the coccyx is fused to the sacrum do not include in the measurement.** (Martin and Knussmann 1988: 195, #2).

61. **Anterior Breadth of the Sacrum**: The maximum transverse breadth of the sacrum at most anterior projection of the auricular surfaces (Figure 3.18). (Martin and Knussmann 1988: 195, #5)

    Instrument: sliding caliper

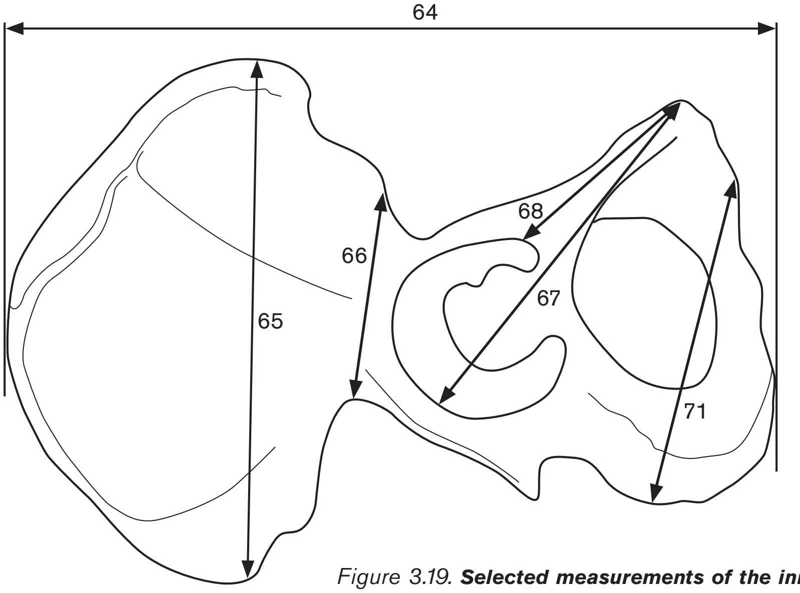

Figure 3.19. ***Selected measurements of the innominate***

62. **Transverse Diameter of Sacral Segment 1**: The distance between the two most lateral points on the superior articular surface measured perpendicular to the mid-sagittal plane (Figure 3.19). (Martin and Knussmann 1988: 195, #19).

    Instrument: sliding caliper

    Comment: The measurement should be taken on the superior articular surface of S1.
    When lipping of the articular surface is present approximate the original articular borders. This measurement is perpendicular to the AP Diameter of S1.

63. **Anterior-Posterior Diameter of Sacral Segment 1**: The distance between the anterior and posterior borders of the superior articular surface of S1, taken in the mid-sagittal plane. (Martin and Knussmann 1988: 195, #18).

    Comment: The measurement should be taken on the superior articular surface of S1. When lipping of the articular surface is present approximate the original articular borders. This measurement is perpendicular to the Transverse Diameter of S1.

64. **Maximum Innominate Height**: The distance from the most superior point on the iliac crest to the most inferior point on the ischial tuberosity (Figure 3.19). (Martin and Knussmann 1988: 213, #1)

    Instrument: osteometric board or spreading caliper

    Comment: When using an osteometric board, place the ischium against the vertical end board and press the movable upright against the iliac crest. Move the ilium sideways and up and down to obtain the maximum distance (Hrdlicka 1920: 135).

65. **Maximum Iliac Breadth**: The distance from the anterior superior iliac spine to the posterior superior iliac spine (Figure 3.19). (Martin and Knussmann 1988: 213, #2).

    Instrument: spreading caliper

66. **Minimum Iliac Breadth (WIB)**: The minimum distance measured from the area below the anterior inferior iliac spine to the most inward curvature of the greater sciatic notch (Figure 3.19).

67. **Maximum Pubis Length (XPL)**: The distance between symphysion (the most superior point on the symphyseal face) to the farthest point on the acetabular rim.

    Comment: The measurement is taken to the rim itself (not inside or outside of the rim).

68. **Minimum Pubis Length (WPL)**: The distance between symphysion (the most superior point on the symphyseal face) to the closest point on the acetabular rim (Figure 3.19).

    Comment: The measurement is taken to the rim itself (not inside or outside of the rim).

69. **Ischial Length (ISL)**: The distance from the point on the acetabular rim where the iliac blade meets the acetabulum to the most medial point on the epiphysis of the ischial tuberosity (Figure 3.20).

    Comment: If the borders of the epiphysis cannot be determined, do not take this measurement.

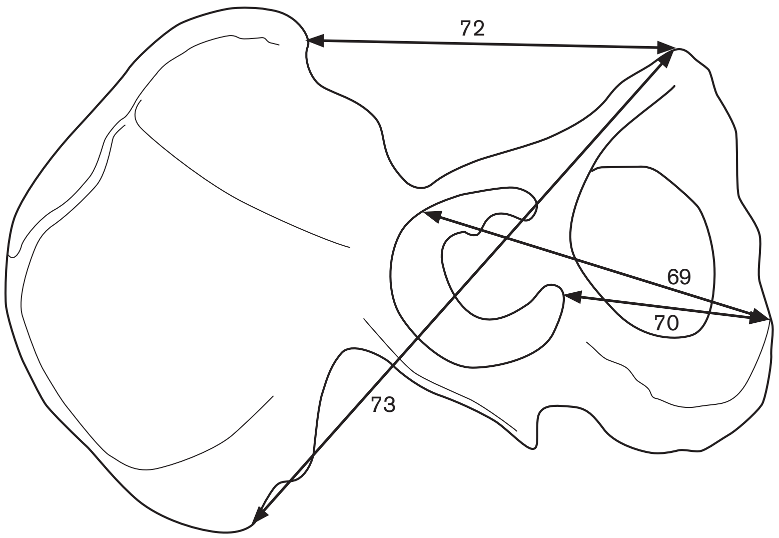

Figure 3.20. ***Lateral view of innominate showing selected measurements.***

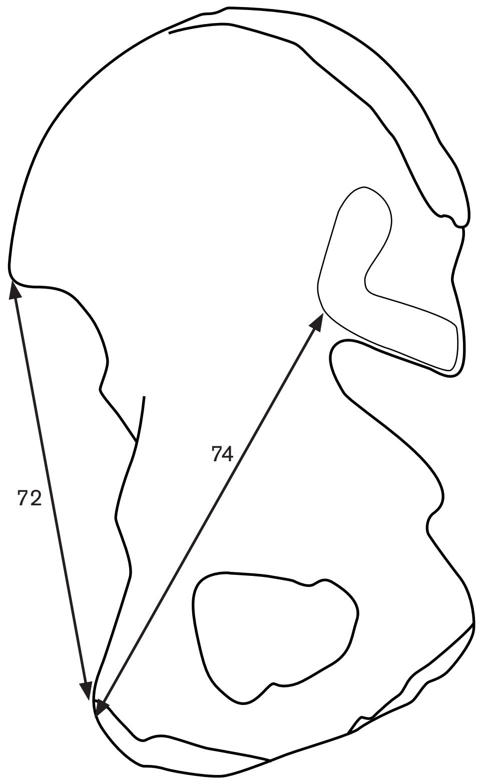

Figure 3.21. ***Dorsal view of the innominate with selected measurements.***

70. **Minimum Ischial Length (WISL)**: The distance from the most medial point on the epiphysis of the ischial tuberosity to the closest point on the acetabular rim (Figure 3.20).

    Comment: If the borders of the epiphysis cannot be determined, do not take this measurement.

71. **Maximum Ischiopubic Ramus Length (XIRL)**: The distance from the most inferior point on the symphyseal face to the most distant point on the ischial tuberosity (Figure 3.19).

72. **Anterior Superior Iliac Spine to Symphysion (ASISS)**: Measurement from the apex of the anterior superior iliac spine (most projecting area or point) to symphysion (Figure 3.20).

73. **Maximum Posterior Superior Iliac Spine to Symphysion (PSISS)**: Maximum measurement from the posterior border of the posterior superior iliac spine to symphysion (Figure 3.20).

74. **Minimum Apical Border to Symphysion (WAS)**: Minimum measurement from symphysion to the apex (anterior border) of the auricular surface (Figure 3.21).

75. **Maximum Length of the Femur**: The distance from the most proximal point on the head of the femur to the most distal point on the medial or lateral femoral condyle (Figure 3.22). (Martin and Knussmann 1988: 216, #1).

    Instrument: osteometric board

    Comment: Place the femur parallel to the long axis of the osteometric board and resting on its posterior surface. Press the medial or lateral condyle against the vertical end board while applying the movable upright to the femoral head. Move the bone up, down, and sideways until the maximum length is obtained (Hrdlicka 1920: 128).

76. **Bicondylar Length of the Femur**: The distance from the most proximal point on the head of the femur to a plane drawn between the inferior surfaces of the distal condyles (Figure 3.23).

    Instrument: osteometric board

    Comment: Place the femur on the osteometric board so that the bone is resting on its posterior surface. Press both distal condyles against the vertical end board while applying the movable upright to the head of the femur (Martin and Knussmann 1988: 216, #2; Hrdlicka 1920: 128).

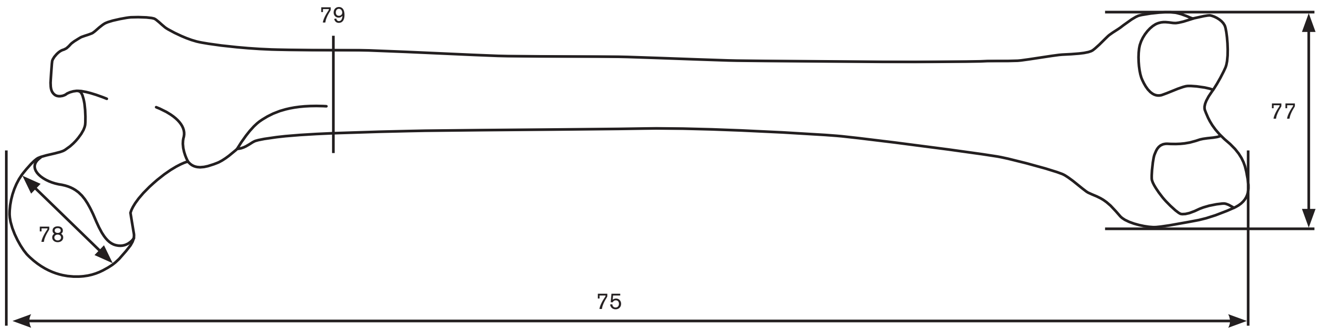

Figure 3.22. ***Maximum length of femur + Epicondylar breadth of femur.***

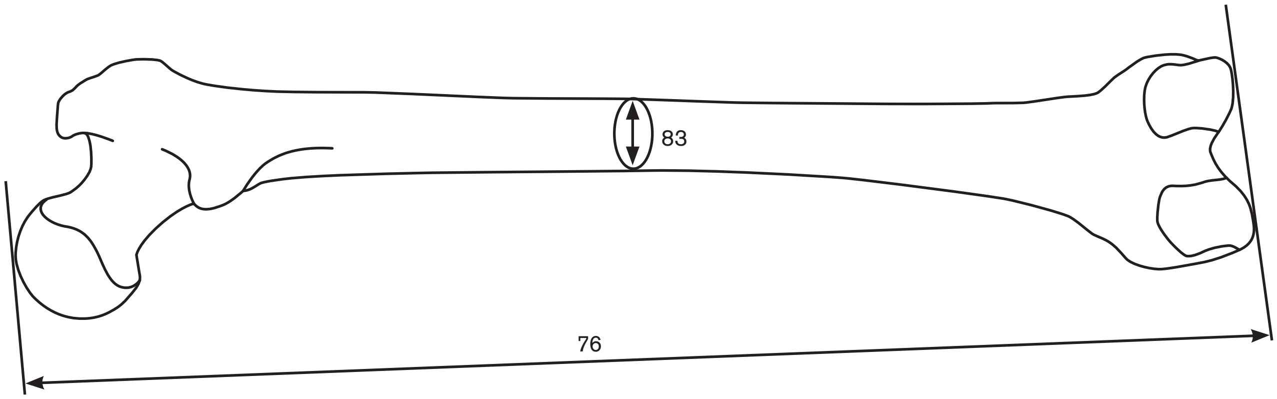

Figure 3.23. ***Bicondylar length of the femur + Circumference of femur at midshaft.***

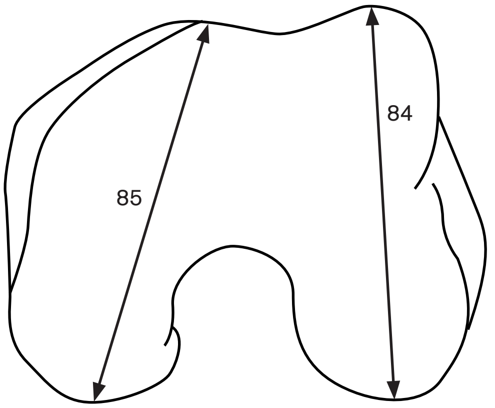

Figure 3.24. ***Lengths of femoral condyles.***

77. **Epicondylar Breadth of the Femur**: The distance between the two most projecting points on the epicondyles (Figure 3.22).

    Instrument: osteometric board

    Comment: Place the femur on the osteometric board so that it is resting on its posterior surface. Press one of the epicondyles against the vertical end board while applying the movable upright to the other condyle. (Martin and Knussmann 1988: 218, #21).

78. **Maximum Diameter of the Femur Head**: The maximum diameter of the femur head measured on the border of the articular surface (Figure 3.22).

    Instrument: sliding caliper

    Comment: Rotate the arms of the caliper around the femur head to find the maximum diameter. (Dwight 1905: 21; Montagu 1960:70).

NOTE: Although the next two measurements (#79 and #80) have higher than acceptable interobserver error values, they provide useful information for certain analyses, so they are included here.

79. **Transverse Subtrochanteric Diameter of the Femur**: The transverse diameter of the proximal portion of the diaphysis at the point of its greatest lateral expansion (see Figure 3.22 for approximate location on the femoral shaft for this measurement).

    Instrument: sliding caliper

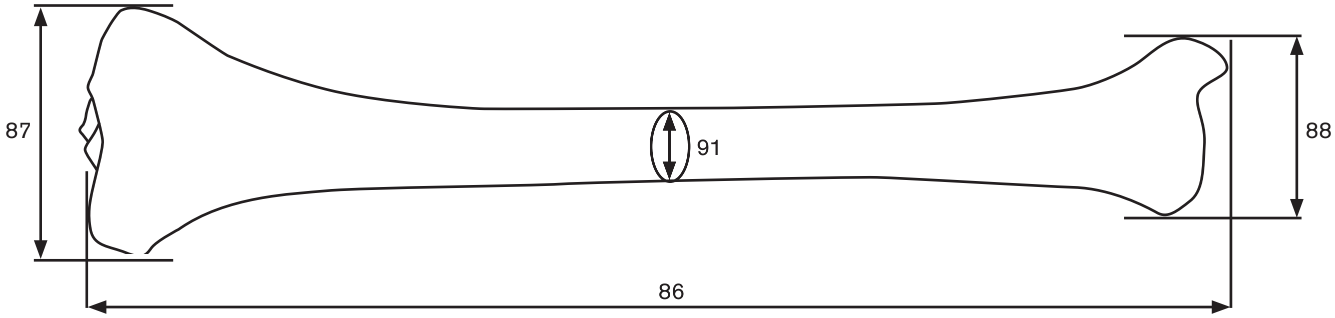

Figure 3.25. ***Selected measurements of the tibia.***

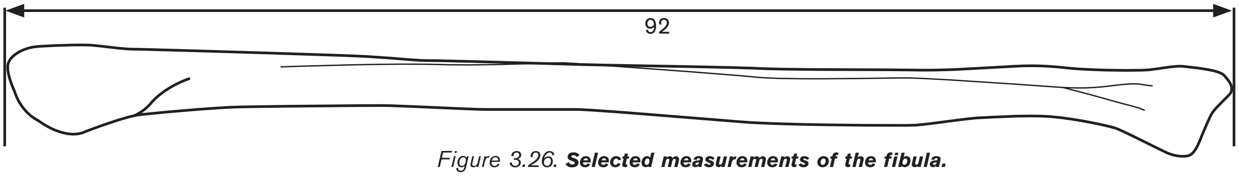

Figure 3.26. ***Selected measurements of the fibula.***

Comment: The transverse diameter is oriented parallel to the anterior surface of the femur neck. Close attention should be paid to assessing this plane in femoral necks with a significant degree of torsion. In cases where this cannot be determined (e.g. where the lateral surfaces remain parallel) this measurement is recorded in the region 2-5 cm below the lesser trochanter. (Martin and Knussman 1988:217 #9).

80. **Anterio-posterior Subtrochanteric Diameter of the Femur**: The anterior-posterior diameter of the proximal end of the diaphysis measured perpendicular to the transverse diameter at the point of the greatest lateral expansion (See definition #79 and Figure 3.22 for approximate location on the femoral shaft for this measurement.). This diameter is oriented perpendicular to the anterior surface of the femur neck. (Martin and Knussman 1988:217 #10).

    Instrument: sliding caliper

81. **Maximum Midshaft Diameter of the Femur**: The maximum diameter of the femoral shaft taken at midshaft.

    Instrument: sliding caliper

    Comment: Mark the midshaft of the bone with a pencil. Using sliding calipers to measure with one hand, rotate the bone with the other hand until the maximum diameter is obtained.

82. **Minimum Midshaft Diameter of the Femur**: The minimum diameter of the femoral shaft taken at midshaft.

    Instrument: sliding caliper

    Comment: Mark the midshaft of the bone with a pencil.

    Using sliding calipers to measure with one hand, rotate the bone with the other hand until the minimum diameter is obtained.

83. **Circumference of the Femur at Midshaft**: The circumference measured at the midshaft (Figure 3.23).

    Instrument: tape

    Comment: If the linea aspera is unusually hypertrophied at midshaft, this measurement should be recorded approximately 10 mm above the midshaft (Martin and Knussmann 1988:217 #8).

84. **Maximum Antero-posterior Length of the Lateral Condyle**: The distance between the most anterior and posterior points on the articular surface of the lateral condyle (Figure 3.24). (Martin and Knussmann 1988:219 #23).

    Instrument: sliding caliper

    Comment: Holding the femur with the distal end up, orient the measuring arms of the caliper with the long axis of the bone to obtain this measurement.

85. **Maximum Antero-posterior Length of the Medial Condyle**: The distance between the most anterior and posterior points on the articular surface of the medial condyle (Figure 3.24). (Martin and Knussmann 1988:219 #24).

    Instrument: sliding caliper

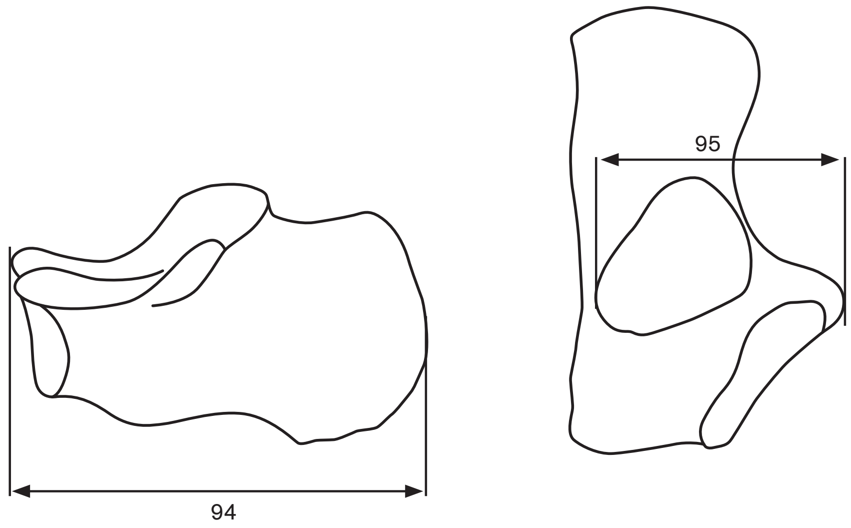

Figure 3.27. ***Maximum Length and middle breadth of the calcaneus.***

Comment: Holding the femur with the distal end up, orient the measuring arms of the caliper with the long axis of the bone to obtain this measurement.

86. **Length of the Tibia**: The distance from the superior articular surface of the lateral condyle of the tibia to the tip of the medial malleolus (Martin and Knussmann 1988: 220, #1) (Figure 3.25).

    Instrument: osteometric board

    Comment: An osteometric board with a hole for the intercondylar eminence makes this measurement easier to take. Place the tibia on the osteometric board resting on its posterior surface with the longitudinal axis of the bone parallel to the board (Hrdlicka 1920: 129). If using an osteometric board without a hole, place the tibia on the osteometric board so that it the long axis is parallel to the board. The measurement is take from the lateral condyle to the tip of the medial malleolus.

87. **Maximum Proximal Epiphyseal Breadth of the Tibia**: The maximum distance between the two most projecting point on the margins of the medial and lateral condyles of the proximal epiphysis (Figure 3.25).

    Instrument: osteometric board

    Comment: Place the tibia on the osteometric board resting on its posterior surface. Press the lateral condyle against the vertical end board, and place the movable upright against the medial condyle. Tibiae exhibiting marked torsion may have to be rotated to obtain the maximum breadth (Martin and Knussmann 1988: 221, #3).

88. **Distal Epiphyseal Breadth of the Tibia**: The distance between the most medial point on the medial malleolus and the lateral surface of the distal epiphysis (Figure 3.25).

    Instrument: osteometric board

    Comment: Place the two lateral protrusions of the distal epiphysis against the fixed side of the osteometric board and move the sliding board until it contacts the medial malleolus (Martin and Knussmann 1988: 221, #6).

89. **Maximum Midshaft Diameter of the Tibia**: The maximum diameter of the tibial shaft taken at midshaft.

    Instrument: sliding caliper

    Comment: Mark the midshaft of the bone with a pencil.

    Using sliding calipers to measure with one hand, rotate the bone with the other hand until the maximum diameter is obtained.

90. **Minimum Midshaft Diameter of the Tibia**: The minimum diameter of the tibial shaft taken at midshaft.

    Instrument: sliding caliper

    Comment: Mark the midshaft of the bone with a pencil. Using sliding calipers to measure with one hand, rotate the bone with the other hand until the minimum diameter is obtained. (Martin and Knussmann 1988:573 #9a).

91. **Circumference of the Tibia at the Midshaft**: The circumference measured at the level at the midshaft (Figure 3.25). (Martin and Knussmann 1988:574 #10a).

Instrument: tape

92. **Maximum Length of the Fibula**: The maximum distance between the most superior point on the fibular head and the most inferior point on the lateral malleolus (Figure 3.26). (Martin and Knussmann 1988: 222, #1).

Instrument: osteometric board

Comment: Place the fibula on the osteometric board and place the tip of the lateral malleolus against the vertical end board. Press the movable upright against the proximal end of the bone while moving it up and down and sideways to obtain the maximum length.

93. **Maximum Diameter of the Fibula at Midshaft**: The maximum diameter at the midshaft. (Martin and Knussmann 1988: 222, #2).

Instrument: sliding caliper

Comment: Find the midpoint on the osteometric board and mark with a pencil. Place the diaphysis of the fibula between the two arms of the caliper while turning the bone to obtain the maximum diameter.

94. **Maximum Length of the Calcaneus**: The distance between the most posteriorly projecting point on the calcaneal tuberosity and the most anterior point on the superior margin of the articular facet for the cuboid measured in the sagittal plane (Figure 3.27). (Martin and Knussmann 1988:225 #1).

Instrument: sliding caliper or osteometric board

Comment: Excessive calcaneal spurs should be omitted from this measurement.

95. **Middle Breadth of the Calcaneus**: The distance between the most laterally projecting point on the dorsal articular facet and the most medial point on the sustentaculum tali (Figure 3.27).

Instrument: sliding caliper

Comment: The two measuring points lie at neither the same level, nor in a plane perpendicular to the sagittal plane. Span the calcaneus from behind with the blunt arms of the caliper so that the caliper is positioned in a flat and transverse plane across the bone (Martin and Knussmann 1988: 225, #2).

REFERENCES

Adams BJ, and Byrd JE.
2002. Interobserver variation of selected postcranial skeletal measurements. J Forensic Sci 47(6):1193-1202.

Ayers, HG, RL Jantz, and PH Moore-Jansen
1990 Giles and Elliot race Discriminant Functions Revisited: A Test Using Recent Forensic Cases. In: Skeletal Attribution of Race, G.W. Gill and S. Rhine, editors, pgs. 65-71. Maxwell Museum of Anthropology, Albuquerque.

Baumgarten SE, Ousley SD, Decker SJ, and Shirley NR.
2015 A transparent method for sex estimation using refined measurements of the innominate. Am J Phys Anthropol 156(S60

Bennett, KA
1993 A Field Guide for Human Skeletal Identification. Second Edition. Springfield: Charles C. Thomas.

Boldsen JL, GR Milner, LW Konigsberg, JW Wood
2002 Transition Analysis: A New Method for Estimating Age from Skeletons. In Paleodemography: Age Distributions from Skeletal Samples. Hoppa RD and Vaupel JW (eds), pp. 73-106. Cambridge University Press, Cambridge.

Committee on Identifying the Needs of the Forensic Sciences Community NRC.
2009 Strengthening Forensic Science in the United States: A Path Forward.

Dwight, T
1905 The size of the articular surfaces of the long bones as characteristics of sex; an anthropological study. American Journal of Anatomy 4: 19-32.

Erickson, MF
1982 How “Representative” is the Terry Collection? Evidence from the Proximal Femur. American Journal of Physical Anthropology 59:345-350.

Howells, WW
1973 Cranial Variation in Man. Papers of the Peabody Museum of Archaeology and Ethnology, 67. Harvard University, Cambridge Massachusetts.

Hrdlicka, A
1920 Anthropometry. The Wistar Institute of Anatomy and Biology, Philadelphia.

Jantz, RL
1994 The Mismeasure of the Tibia. Paper presented at the 46th Annual Meeting of the American Academy of Forensic Sciences, San Antonio, February 14-1 9.

Jantz, RL, and PH Moore-Jansen
1988 A Data Base for Forensic Anthropology: Structure, Content, and Analysis. Report of Investigations no. 47, Department of Anthropology, The University of Tennessee, Knoxville.

1990 Stature Estimation from Long Bone Lengths of a Recent Forensic Sample: A Modification of Existing Standards. Paper presented at the 44th Annual Meeting of the American Academy of Forensic Sciences, Cincinnati, February 19-24.

Jantz, RL, and SD Ousley
1993 FORDISC 1.O: Computerized Forensic Discriminant Functions. The University of Tennessee, Knoxville.

Jantz, RL, and SD Ousley
1996 FORDISC 2.O: Computerized Forensic Discriminant Functions. The University of Tennessee, Knoxville.

Jantz R, and Ousley S.
2005 FORDISC 3.0: Computerized Forensic Discriminant Functions. Knoxville: University of Tennessee.

Lovejoy CO, RS Meindl, TR Pryzbeck, RP Mensforth
1985 Chronological Metamorphosis of the Auricular Surface of the Ilium: A New Method for the Determination of Adult Skeletal Age at Death. American Journal of Physical Anthropology 68:15-28.

Mann RW, SA Symes, WM Bass
1987 Maxillary Suture Obliteration: Aging the Human Skeleton Based on Intact or Fragmentary Maxilla. Journal of Forensic Sciences 32:148-157.

Marks, MK
1990 Testing Confidence Intervals for Stature Estimation in a Modern Forensic Anthropology Sample. Paper presented at the 44th Annual Meeting of the American Academy of Forensic Sciences, Cincinnati, February 19-24.

Martin, R
1956 Lehrbuch der Anthropologie. Revised Third Edition, Volume 3, edited by Karl Sailer. Gustav Fischer Verlag, Stuttgart.

1957 Lehrbuch der Anthropologie. Revised Third Edition, Volume 4, edited by Karl Saller. Gustav Fischer Verlag, Stuttgart.

Martin, R and R Knussmann
1988 Anthropologie: Handbuch der vergleichenden Biologie des Menschen. Stuttgart: Gustav Fischer.

McKern TW, andTD Stewart
1957 Skeletal Age Changes in Young American Males. Technical Report EP-45. Quartermaster Research and Development Center, US Army, Natick, Massachusetts.

Meadows, L, and RL Jantz
1992 Secular Changes in Lower Limb Bone Proportions and their implications in Stature Estimation. Paper presented at the 44th Annual Meeting of the American Academy of Forensic Sciences, New Orleans, February 17-22.

Milner GR and JL Boldsen
2012 Transition Analysis: A Validation Study with Known-Age Modern American Skeletons. American Journal of Physical Anthropology 148:98-110.

Moore-Jansen, PH
1991 Morphometric Variation in the Human Radius. Paper presented at the 43th Annual Meeting of the American Academy of Forensic Sciences, Anaheim, February 18-23.

PH Moore-Jansen and RL Jantz
1986 A computerized skeletal data bank for forensic anthropology - Knoxville: University of Tennessee

1989 Data collection procedures for forensic skeletal material. Report of investigations, 48, Knoxville.

Montagu, MFA
1960 A Handbook of Anthropometry. Charles C. Thomas, Springfield, Illinois.

Murray, KA
1990 Statistical test of the Auricular Surface Ageing Technique on the Terry Collection. Paper presented at the 44th Annual Meeting of the American Academy of Forensic Sciences, Cincinnati, February 19-24.

Ousley, SD
1994 Should we estimate “Biological” or Forensic Stature? Paper presented at the 46th Annual Meeting of the American Academy of Forensic Sciences, San Antonio, February 14-19.

Ousley, SD, and RL Jantz
1992 The Forensic Data Bank: Some Results after 5 years and 1,000 cases. Paper presented at the 44th Annual Meeting of the American Academy of Forensic Sciences, New Orleans, February 17-22.

1993 Postcranial Racial Determination: Discriminant Functions from the Forensic Data Bank. Paper presented at the 45th Annual Meeting of the American Academy of Forensic Sciences, Boston, February 15-20.

Singh, P, and MK Bhasin
1968 Anthropometry. Barthi Bhawan, Delhi, India.

Todd, TW
1920 Age changes in the pubic bone. I. The male white pubis. American Journal of Physical Anthropology 3.3: 285-334.

Webb, PA, and JM Suchey
1985 Epiphyseal Union of the Anterior Iliac Crest and Medial Clavicle in a Modern Multiracial Sample of American Males and Females. American Journal of Physical Anthropology 68:457-466.

Willcox, AW, PS Sledzik, WF McCarthy, and MS Miccozzi
1992 Determination of Sex from the Distal Humerus. Paper presented at the 44th Annual Meeting of the American Academy of Forensic Sciences, New Orleans, February 17-22.

RECORDING FORM INFORMATION

Case Information

The forensic recording forms consist of four pages with nine sections. The sections are for recording 1) General information including age, sex, race, state of preservation, pathological lesions, medical history, and time and manner of death (this section cannot be fully completed unless the case is a positive identification) 2) Skeletal inventory and research materials 3) Epiphyseal closure 4) Transition Analysis Age Information 5) Forensic Measurements. An optional dental recording form is include for use (Appendix B).

Several cases indentifying items are listed at the top of each page of the recording form for each forensic case. These items should be completed on each page to ensure that the data sheets of one case belong together in case of separation.

Collection I.D. /Case# : The label or number that uniquely references a particular forensic case and any associated records and research materials.

Curator/Address: List the name and/or address of the agency or institution responsible for processing and curating the forensic case in question. This should refer to the address where any records and research materials associated with the case may be located. However, if an individual is returned for burial, as is often the case, record the address of the agency or institution responsible for completing the forensic recording sheet.

Recorder: Enter the name of the person or persons participating in the observation and recording of the information provided on the forensic recording sheet.

Date: Enter the month, day, and year when the data are collected.

I.D. Name: List the name of the identified subject if in compliance with regulations for the release of such data.

Means of Identification: List the method or methods used to make a positive identification (visual, soft tissue, skeletal, fingerprints, dental records, dental or skeletal radiographs). Note that this may apply to positive, tentative, ad known sex and race identifications.

Positive Identification: Record the status of identification of the case in question. Write in “YES” or a checkmark to show that the subject has been positively identified. Some cases may warrant recording a tentative identification. Write in “NO” or leave blank to show that the subject has been neither positively nor tentatively identified. Several levels of identification are possible. While positive identification means the identity of the person is known, tentative identifications are applied to cases if sex and/or race can be determined from soft tissues or hair. For example, a case may be recorded as *Sex: M, Source: soft tissue; Race: N Source: Hair;*. Cases with a definite sex and race can be as valuable for research as positive identification.

Date of Positive Identification: Enter the month, day, and year if the subject was positively identified.

General Information

The first section of the recording form is designed to provide a profile of the subject (Appendix A: page 1). While some observations pertain to all cases, others (Medical History) may require that the forensic case has been positively identified. The individual items are listed on page 1 of the recording form (1-23) and are referenced by number and this manual.

Items 1 through 12 pertain primarily to positively identified subjects, whose medical and personal records, if available, may provide the information requested. Unidentified subjects including "tentative identifications," for whom age, sex, and race have been estimated should be recorded followed by a qualifying statement (e.g. skeletal estimate, soft tissue). Specific methods by which the estimates presented are obtained may be listed in the section for additional comments at the bottom of page 1 of the recording form.

The information requested in items 14 through 24 on page 1 of the recording form, pertain to the nature and circumstances of the forensic case whether or not positively identified and thus apply to all cases. Items 25-31 pertain to all cases to the extent the specified information is available.

The entries for General Information are to be completed as follows:

1. *Sex*: if the sex is definite, enter "M" or "F" accordingly. If not a positive identification, list the criteria on the line immediately following (*source: soft tissue-genitalia*). In cases of unidentified specimens record the specific cranial or postcranial observations that were used for sex estimation.

2. *Race*: If subject is positively identified, record the racial classification. Determination of racial affiliation is often a complex matter and it is recommended that major racial classification be followed by ethnic affiliation or components of ethnic affiliation (e.g. "Amerindian-Navaho"; "Mongoloid-South Korean"). Other "catch all" terms (Hispanic, Oriental, etc.), should also be explained further if possible, List the source of the race determination on the line immediately following. If positive data are unavailable, record the specific cranial or postcranial observations used for race classification.

3. *Age*: If subject is positively identified list the age of the subject as determined form available documentation (driver's license, missing person bulletin). list the age source on the line immediately following. If no documented age information is available, indicate the estimated skeletal age range, or the appropriate age group. A = Adult (*Age 18+*); S = subadult (*Age 12-17*); C=child (*Age 1-11*); I = infant(*Age 0-1*); F = fetus/unborn.

4. *Stature*: If subject is positively identified, enter the height of the individual from the records or other identification. When possible, specify the age when height was recorded, and list the source of the stature determination (police records, drivers license) on the line immediately following. We are aware that driver's license data may be inaccurate. Driver's licenses provide the most common source of forensic stature available, however, and should not be ignored (Ousley 1994). Multiple sources of stature are welcomed here and may be recorded on this line or in additional comments. Cadaver length should be included if available. Indicate cadaver length by writing "CADAVER" immediately following the measurement, and state method by which the length is obtained if known (Tape). If both living and cadaver measurements are available, include cadaver length in additional comments (#31).

5. *Weight in lbs.*: If subject is positively identified, list the appropriate live weight of the individual from documentation. Record the source of the weight determination on the line immediately following. Cadaver weight may be substituted for live weight if indicated.

6. *Handedness*: List if known whether the subject was right or left handed, For our purposes, handedness is indicated by the favored writing hand. List the source of this information on the line immediately following.

7. *Date of Birth*: Pertains to positively identified subjects. Enter the date of birth determined from available records (driver's license, birth certificate). Do not estimate date from chronological age. Indicate the source of this information on the line immediately following.

8. *Place of Birth*: List the appropriate country, state, county and municipality where born. This pertains to positively identified subjects only. A more general statement of "Place of Origin" may be substituted for " Place of Birth" when the latter is not clearly stated in any available documentation (State, Country, or Region). Record the information source on the line immediately following.

9. *Occupation*: Record the occupation of the subject and the number of years in this occupation if known. List the source of this information on the line immediately following. Pertains primarily to positively identified subjects.

10. *Blood Type*: List the blood type of the subject (e.g. ABA, Rh) if known and list the information source on the line immediately following.

11. *Number of Births*: This and the following line pertain to positively identifed female subjects only. Enter the number of recorded births from available documentation.If a specific number of births is unavailable, indicate if known, whether subject is "PAROUS" as opposed to "NULLIPAROUS". Note that number of children does not necessarily reflect number of births or pregnancies, list source of this information on the line immediately following.

12. *Number of Pregnancies*: Record the number of documented pregnancies. Record source of this information on the line immediately following.

NOTE: List source documents used to determine Items 1-12 on the lines immediately following them. If space does not permit the listing of all source records in this item enter the sources under Additional Comments.

13. *Date Reported Missing*: Enter the month, day and year when subject was reported missing or last seen.

14. *Date of Discovery*: Enter the month, day, and year when body was discovered.

15. *Date of Death*: Enter the date of death of the subject if documented by witnesses.

16. *Time since Death*: List the estimated period of time since death in days, weeks, months, or years as determined by a medical examiner or coroner. Use any of the following terms: Homicide, Suicide, Accidental, Natural, and Unknown.

18. *Deposit/Exposure*: State if subject was found buried, in water, or above ground, and whether exposed or unexposed.

19. *Depth of Deposit*: Id subject was buried, list burial depth below surface.

20. *Estimated Period of Decay*: List the estimated period of decay as determined from the extent of decomposition of the subject and presence or absence of seasonal indicators (parasites, beetles). Record estimation method used.

21. *Nature of Remains*: Describe the material compromising the forensic case under examination. Record whether it has soft tissue or "Skeletal Remains,: and specify the extent of the remains ("Skull only", "Nearly complete skeleton", "Partial skeleton - no skull"). A more detailed skeletal inventory is in item 32.

22. *Place of Discovery*: Describe briefly the location where the subject was recovered (woods, open field, river bank, etc.).

23. *State*: Enter the name of the state in which the subject was recovered.

24. *County*: Enter the name of the county in which the subject was recovered.

25. *Municipality*: Enter the name of the city, town, village, or other municipality in which the subject was recovered.

26. *Medical History*: List an aberrant condition exhibited by the individual (drug or alcohol abuse, diabetes, heart disease, mental diseases, stress disorders, trauma, etc.) according to available medical records. This data comes only from positively identified remains.

27. *Congential Malformations*: Follow procedure for Item 25. List any congenital or developmental defects (cleft palate, congenital hip dislocation, polydactylies, spina bifida).

28. *Dental Records*: List any dental defects, antemortem tooth loss, or alveolar resorption observed directly on the subject. Record anomalies (supermumerary teeth, congenitally absent teeth, shovel-shaped incisors,, Carabelli's cusp, etc.), extent of tooth wear and decay. Item 28 pertains to all forensic cases exhibiting the dentition or portions thereof. If subject is positively identified, list any appropriate information which may derived from existing dental charts, or x-rays. Alternatively, dental records including fillings may be illustrated on the recording sheets provided in Appendix B. See also Item 32.

29. *Bone Lesions*: Describe and list the location of any pathological lesions, healed fractures, injuries, or degenerative changes observed directly on the bone. The bone lesions listed should not reflect perimortem injuries (see Item 30).

30. *Perimortem Injuries*: Describe and list the location of any visible fractures or injuries incurred around time of death (stab wounds, gunshot wounds, fractures, etc.).

31. *Additional Comments*: List any additional comments regarding the forensic case or use this space to elaborate on specific items (e.g. date of birth of children). Label each comment with the appropriate item number.

Inventory

Item 32 concerns the general inventory of skeletal and dental remains. Items 33 to 37 refer to the inventory of other materials including radiographs, photographs, casts and tissue samples.

32. *Skeletal Inventor*y: Enter one of the codes listed at the top of page 2 of the recording form which best describes the status of a bone or tooth (enter codes for both the right and left side where appropriate). The codes are as follows:

    1 - present, complete
    2 - present, fragmented
    3 - absent (In case of the dentition, this code pertains to "post mortem" or "undetermined" tooth loss.
    4 - absent, antemortem (This code pertains primarily to the dentition and indicates antemortem tooth loss.)
    5 - unerupted (pertain to dentition only)
    6 - congenitally missing

    If there is a complete crania, mandible, or postcrania you can enter the appropriate code for each of these general categories rather than specifying the code for each bone (*Cranium: 1*)

33-37 *Other Materials*: List any available skeletal or other research materials associated with the forensic case in the space provided at the bottom of page 2 of the recording form (Appendix A). Specify the nature of the available materials and record the address to which inquiries regarding such materials maybe made, if different from the Curator/Address list at the top of the recording form. Where radiographs or photographs are reported, indicate proper orientation. Also list whether radiographs are antemortem or postmortem.

APPENDIX A

Forensic Recording Forms

COLLECTION ID CASE #:______________________________ ID NAME:__________________________________

CURATOR/ADDRESS: _________________________________ MEANS OF ID:______________________________

RECORDER:_______________________DATE:__________POSITIVE IDENTIFICATION?_______ DATE:_________

-- GENERAL INFORMATION --

	SOURCE		*SOURCE*
1. SEX:______	___________________________	7. DATE OF BIRTH:__________	_______________________
2. RACE:______	_________________________	8. PLACE OF BIRTH:__________	______________________
3. AGE:______	__________________________	9. OCCUPATION:______________	_____________________
4. STATURE:______	__________________________	10. BLOOD TYPES:______	__________________________
5. WEIGHT :______	__________________________	11. BIRTHS :______	__________________________
6. HANDEDNESS:_______	__________________________	12. PREGNANCIES:______	__________________________

13. DATE REPORTED MISSING: ________________________

14. DATE OF DISCOVERY: _____________________________

15. DATE OF DEATH: __________________________________

16: TIME SINCE DEATH: ______________________________

17. MANNER OF DEATH: ______________________________

18. DEPOSIT/EXPOSURE: _____________________________

____

____

19. DEPTH IN CM (IF BURIED): ________

20. EST. PERIOD OF DECAY:_______________________

21. NATURE OF REMAINS: ____

22. PLACE OF DISCOVERY (Area): ____

____

23. STATE:______________________ 24. COUNTY:___________________________ 25. MUNICIPALITY________________

26. MEDICAL HISTORY: ____

____

27. CONGENITAL MALFORMATIONS: ____

____

28. DENTAL RECORDS (specify): ____

____

29. BONE LESIONS (Antemortem): ____

____

30. PERIMORTEUM INJURIES: ____

____

31. ADDITIONAL COMMENTS: ____

____

____

____

COLLECTION ID CASE #:______________________________ CURATOR/ADDRESS: ______________________________

------------------------------ SKELETAL INVENTORY --------------------------------

32. INVENTORY: Codes:
1 - present complete
2 - present fragmentary
3 - absent (postmortem)
4 – antemortem loss
5 - unerupted (dentition)
6 – congenitally missing

	LEFT		RIGHT		LEFT		RIGHT
**Cranium**:______							
Frontal:		______		Maxilla:	______		______
Parietal:	______		______	Nasal:	______		______
Occipital:		______		Ethmoid:		______	
Temporal:	______		______	Lacrimal:	______		______
Zygomatic:	______		______	Vomer:		______	
Palate:	______		______	Sphenoid:		______	
**Mandible**: ______							
Body:	______		______	Ramus:	______		______
**Dentition**: ______							
Max. I1:	______		______	Mand. I1:	______		______
Max. I2:	______		______	Mand. I2:	______		______
Max. C:	______		______	Mand. C:	______		______
Max. P1:	______		______	Mand. P1:	______		______
Max. P2:	______		______	Mand. P2:	______		______
Max. M1:	______		______	Mand. M1:	______		______
Max. M2:	______		______	Mand. M2:	______		______
Max. M3:	______		______	Mand. M3:	______		______
**Postcranium**: ______							
Hyoid:		______		Thoracic 1-12:		______	
Clavicle:	______		______	Lumbar 1-5:		______	
Scapula:	______		______	Sacrum:		______	
Humerus:	______		______	Ilium:	______		______
Radius:	______		______	Pubis:	______		______
Ulna:	______		______	Ischium:	______		______
Hand:	______		______	Femur:	______		______
Manubrium:		______		Patella:	______		______
Sternal Body:		______		Tibia:	______		______
Ribs:	______		______	Fibula:	______		______
Atlas:		______		Calcaneus:	______		______
Axis:		______		Talus:	______		______
Cervical 3-7:		______		Foot:	______		______

------------------------------- RESEARCH MATERIALS -------------------------------

33. SKELETAL MATERIALS: ____
34. DENTAL CASTS: ____
35. HISTOLOGICAL SECTIONS: ____
36. RADIOGRAPHSIPHOTOS: ____
37. OTHER (hair, etc.): ____

COLLECTION ID CASE #:______________________________ ID NAME:____

CURATOR/ADDRESS: _________________________________ RECORDER:_______________________DATE:___________

------------------------------ PUBIC SYMPHYSIS --------------------------------

	LEFT	RIGHT
1. SYMPHYSEAL RELIEF	______	______
2. DORSAL SYMPHYSEAL TEXTURE	______	______
3. SUPERIOR PROTUBERANCE	______	______
4. VENTRAL SYMPHYSEAL MARGIN	______	______
5. DORSAL SYMPHYSEAL MARGIN	______	______

------------------------------ SACROILIAC JOINT ------------------------------

	LEFT	RIGHT
1. SUPERIOR DEMIFACE TOPOGRAPHY	______	______
2. INFERIOR DEMIFACE TOPOGRAPHY	______	______
3. SUPERIOR SURFACE CHARACTERISTICS	______	______
4. MIDDLE SURFACE CHARACTERISTICS	______	______
5. INFERIOR SURFACE CHARACTERISTICS	______	______
6. INFERIOR SURFACE TEXTURE	______	______
7. SUPERIOR POSTERIOR ILIAC EXOSTOSES	______	______
8. INFERIOR POSTERIOR ILIAC EXOSTOSES	______	______
9. POSTERIOR EXOSTOSES	______	______

------------------------------ CRANIAL SUTURES ------------------------------

	LEFT		RIGHT
1. CORONAL PTERICA	______		______
2. SAGITTAL OBELICA		______	
3. LAMBDOIDAL ASTERICA	______		______
4. ZYGOMATICOMAXILLARY	______		______
5. INTERPALALATINE		______	

COLLECTION ID CASE #:________________________ RECORDER:________________________ DATE:___________

------------------------------ CRANIAL MEASUREMENTS ------------------------------

Measurement		Measurement	LEFT		RIGHT
1. MAXIMUM CRANIAL LENGTH (g-op):	______	15. NASAL BREADTH:		______	
2. NASIO-OCCIPITAL LENGTH (n-op):	______	16. ORBITAL BREADTH (d-ec):	______		______
3. MAXIMUM CRANIAL BREADTH (eu-eu)	______	17. ORBITAL HEIGHT:	______		______
4. BIZYGOMATIC BREADTH (zy-zy):	______	18. BIORBITAL BREADTH (ec-ec):		______	
5. BASION-BREGMA HEIGHT (ba-b):	______	19. INTERORBITAL BREADTH (d-d):		______	
6. CRANIAL BASE LENGTH (ba-n):	______	20. FRONTAL CHORD (n-b):		______	
7. BASION-PROSTHION LENGTH (ba-pr):	______	21. PARIETAL CHORD (b-l):		______	
8. MAXILLO-ALVEOLAR BREADTH (ecm-ecm):	______	22. OCCIPITAL CHORD (l-o):		______	
9. MAXILLO-ALVEOLAR LENGTH (pr-alv):	______	23. FORAMEN MAGNUM LENGTH:		______	
10. BIAURICULAR BREADTH (ra-ra):	______	24. FORAMEN MAGNUM BR:		______	
11. NASION-PROSTHION HEIGHT (n-pr):	______	25. MASTOID HEIGHT (po-ms):		______	
12. MINIMUM FRONTAL BREADTH (ft-ft):	______	26. BIASTERIONIC BR. (ast-ast):		______	
13. UPPER FACIAL BREADTH (fmt-fmt):	______	27. BIMAXILLARY BR. (zma-zma):		______	
14. NASAL HEIGHT:	______	28. ZYGOORBITALE BR. (zo-zo):		______	

------------------------------ MANDIBULAR-MEASUREMENTS ------------------------------

Measurement	LEFT		RIGHT
29. CHIN. HEIGHT (id-gn):		______	
30. MANDIBULAR BODY HEIGHT:	______		______
31. MANDIBULAR BODY BREADTH:	______		______
32. BIGONIAL BREADTH (go-go):		______	
33. BICONDYLAR BR. (cdl-cdl):		______	

Measurement	LEFT		RIGHT
34. MAX. RAMUS HEIGHT:	______		______
35. *MAX. RAMUS HEIGHT:		______	
36. *MAND. LENGTH:		______	
37. *MAND. ANGLE:		______	

**Record only if mandibulometer if used*

------------------------------ POSTCRANIAL MEASUREMENTS ------------------------------

Measurement	LEFT	RIGHT
**CLAVICLE:**		
38. MAXIMUM LENGTH:	______	______
39. MAX. MIDSHAFT DIAM:	______	______
40. MIN. MIDSHAFT DIAM:	______	______
**SCAPULA:**		
41. HEIGHT:	______	______
42. BREADTH:	______	______
43. GLENOID CAVITY BREADTH:	______	______
44. GLENOID CAVITY HEIGHT:	______	______
**HUMERUS:**		
45. MAXIMUM LENGTH:	______	______
46. EPICONDYLAR BREADTH:	______	______
47. MAX. VERT. HEAD DIAM.:	______	______
48. MAX. MIDSHAFT DIAM.:	______	______
49. MIN. MIDSHAFT DIAM.:	______	______

Measurement	LEFT	RIGHT
**RADIUS:**		
50. MAXIMUM LENGTH:	______	______
51. MAX. MIDSHAFT DIAM.:	______	______
52. MIN. MIDSHAFT DIAM.:	______	______
53. MAX. HEAD DIAMETER:	______	______
**ULNA:**		
54. MAXIMUM LENGTH:	______	______
55. MAX. MIDSHAFT DIAM.:	______	______
56. MIN. MIDSHAFT DIAM.:	______	______
57. PHYSIOLOGICAL LENGTH:	______	______
58. MIN. CIRCUMFERENCE:	______	______
59. OLECRANON BREADTH:	______	______

COLLECTION ID CASE #:_______________________ RECORDER:______________________ DATE:__________

------------------------------ POSTCRANIAL MEASUREMENTS ------------------------------

SACRUM:

60. ANTERIOR HEIGHT:	______
61. ANTERIOR BREADTH:	______
62. TRANSVERSE DIAM. S1:	______
63. A-P DIAMETER S1:	______

	LEFT	RIGHT
**INNOMINATE:**		
64. MAXIMUM HEIGHT:	______	______
65. MAX. ILIAC BREADTH:	______	______
66. MIN. ILIAC BREADTH:	______	______
67. MAX. PUBIS LENGTH:	______	______
68. MIN. PUBIS LENGTH:	______	______
69. ISCHIAL LENGTH:	______	______
70. MIN. ISCHIAL LENGTH:	______	______
71. MAX. ISCHIOPUB RAM L.:	______	______
72. ASIS – SYMPHYSION:	______	______
73. MAX. PSIS – SYMPHYSION:	______	______
74. MIN. APEX – SYMPHYSION:	______	______
**FEMUR:**		
75. MAXIMUM LENGTH:	______	______
76. BICONDYLAR LENGTH:	______	______
77. EPICONDYLAR BREADTH:	______	______
78. MAX. HEAD DIAMETER:	______	______
79. TRANS. SUBTROCH DIAM:	______	______
80. A-P SUBTROCH DIAM:	______	______
81. MAX. MIDSHAFT DIAM.	______	______
82. MIN. MIDSHAFT DIAM.	______	______
83. MIDSHAFT CIRCUM.	______	______
84. MAX AP L. LAT. CONDYLE:	______	______
85. MAX AP L. MED. CONDYLE:	______	______

	LEFT	RIGHT
**TIBIA:**		
86. CONDYLO-MALLEOLAR L.:	______	______
87. MAX. PROX. EPIP. BR.:	______	______
88. DISTAL EPIP. BREADTH:	______	______
89. MAX. MIDSHAFT DIAM.:	______	______
90. MIN. MIDSHAFT DIAM.:	______	______
91. MIDSHAFT CIRCUM.:	______	______
**FIBULA:**		
92. MAXIMUM LENGTH:	______	______
93. MAX. MIDSHAFT DIAM.:	______	______
**CALCANEUS:**		
94. MAXIMUM LENGTH:	______	______
95. MIDDLE BREADTH:	______	______

APPENDIX B

Dental Recording Form

DENTAL CHART

8			9
7			10
6			11
5			12
4			13
3			14
2			15
1			16

RIGHT

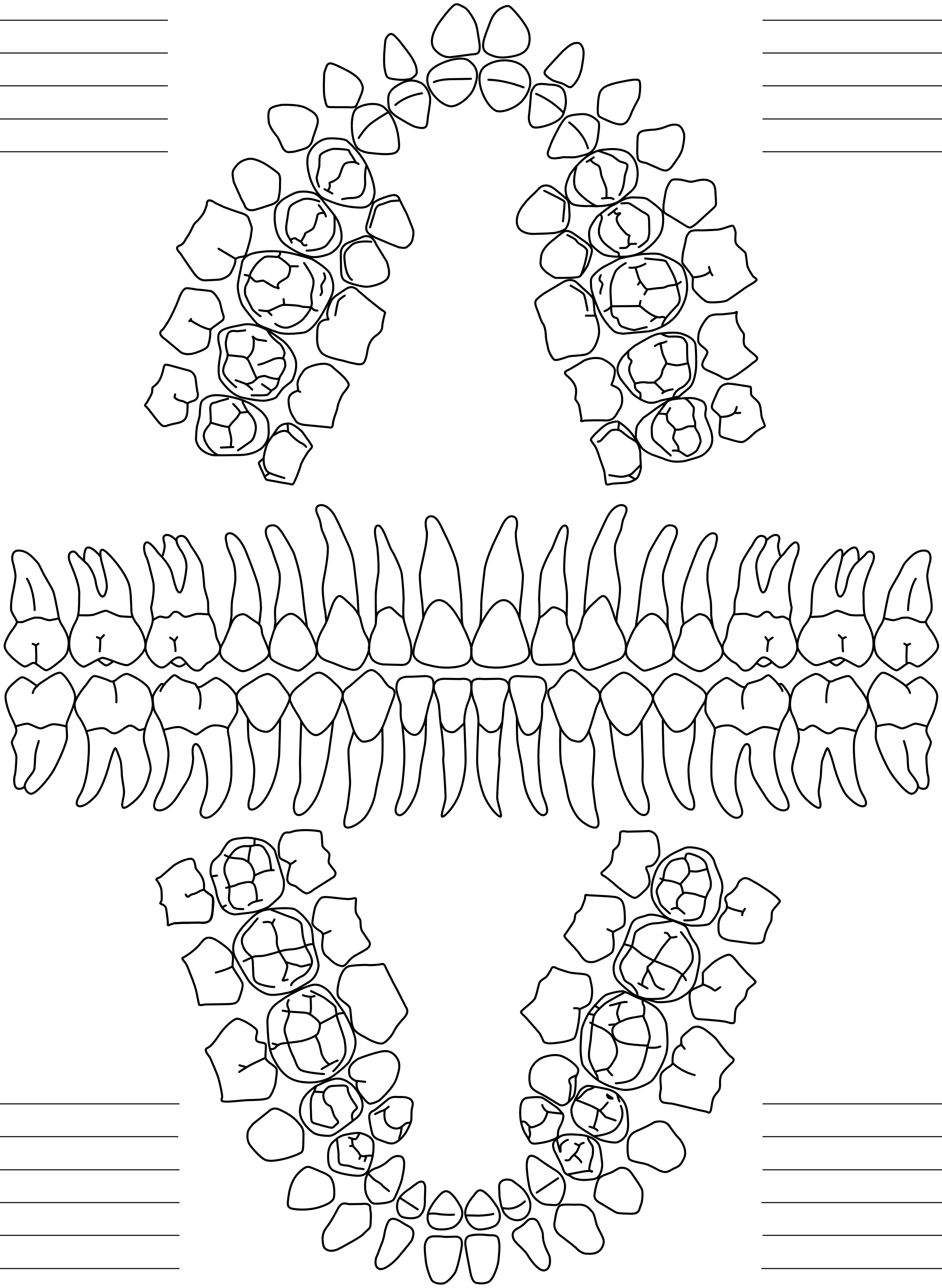

LEFT

32			17
31			18
30			19
29			20
28			21
27			22
26			23
25			24

DECIDUOUS DENTAL RECORD

E ____________________
D ____________________
C ____________________
B ____________________
A ____________________

____________________ F
____________________ G
____________________ H
____________________ I
____________________ J

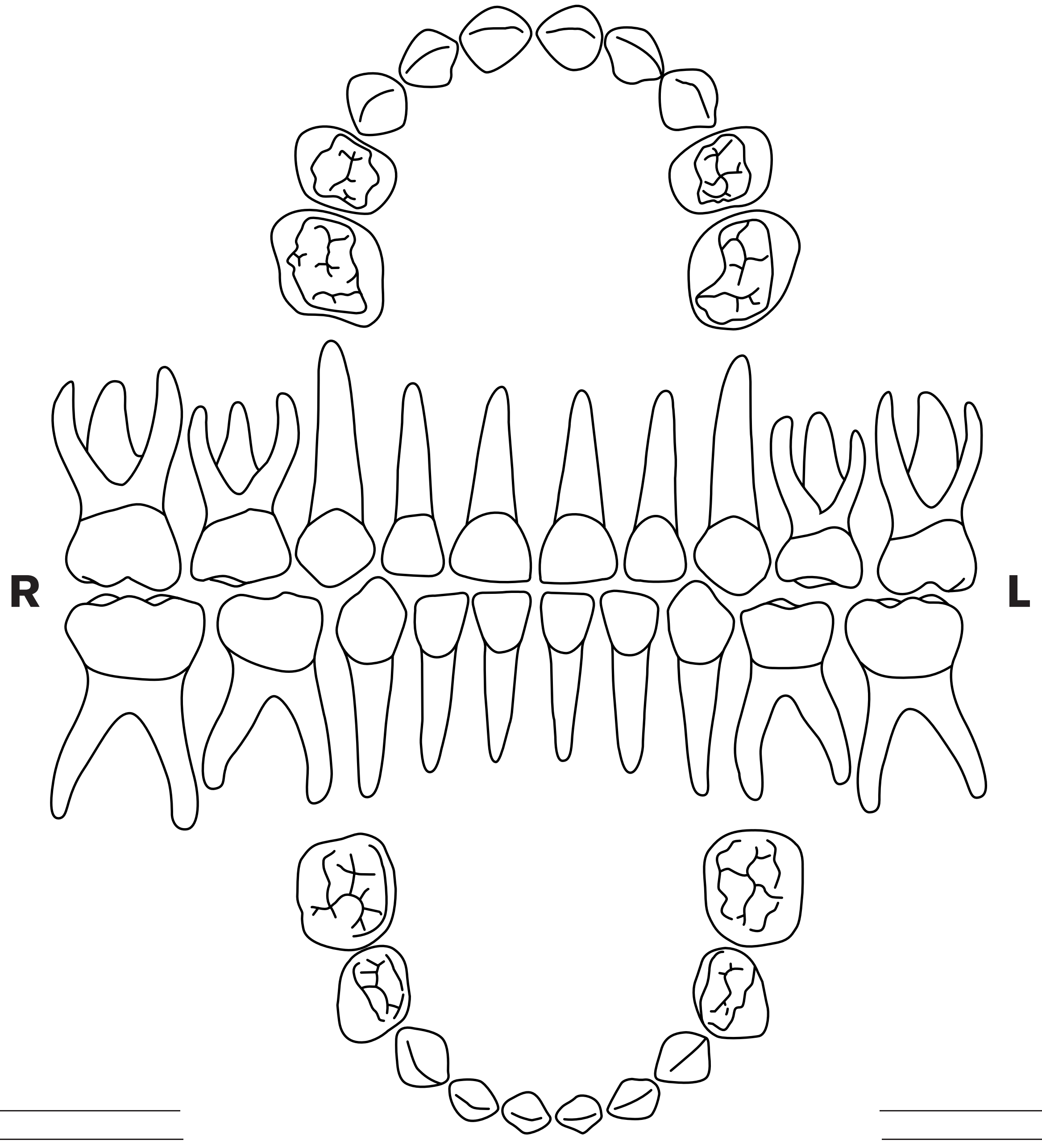

T ____________________
S ____________________
R ____________________
Q ____________________
P ____________________

____________________ K
____________________ L
____________________ M
____________________ N
____________________ O

APPENDIX C : MEASUREMENT ERROR TABLES

MEASUREMENT ABBREVIATIONS (°definition revised, *new measurement)

ABBREVIATION	MEASUREMENT
GOL	maximum cranial length (glabella-opisthocranion)
XCB	maximum cranial breadth (euryon-euryon)
ZYB	bizygomatic breadth (zygion-zygion)
BBH°	basion-bregma height
BNL°	cranial base length (basion-nasion length)
BPL°	basion-prosthion length
MAB	maxillo-alveolar breadth (ectomalare-ectomalare)
MAL	maxillo-alveolar length (prosthion-alveolon)
AUB°	biauricular breadth (radiculare-radiculare)
UFHT°	upper facial height (nasion-prosthion)
WFB	minimum frontal breadth (frontotemporale-frontotemporale)
UFBR	upper facial breadth (frontomalare temporale-frontomalare temporale)
NLH°	nasal height
NLB°	nasal breadth
OBB°	orbital breadth (dacryon-ectoconchion)
OBH	orbital height
EKB	biorbital breadth (ectoconchion-ectoconchion)
DKB°	interorbital breadth (dacryon-dacryon)
FRC	frontal chord (nasion-bregma)
PAC	parietal chord (bregma-lambda)
OCC	occipital chord (lambda-opisthion)
FOL°	foramen magnum length (basion-opisthion)
FOB	foramen magnum breadth
MOW*	mid-orbital width (zygorbitale-zygorbitale)
ASB*	biasterionic breadth (asterion-asterion)
ZMB*	zygomaxillary breadth
MDH°	mastoid length (porion-mastoidale)
MANDIBULAR MEASUREMENTS	mastoid length (porion-mastoidale)
CHIN HT	chin height
MAND BODY HT	mandibular body height
MAND BODY BR	mandibular body breadth
BIGONIAL BR	bigonial breadth of mandible
BICOND BR	bicondylar breadth of mandible
WRBR°	minimum ramus breadth
XRHT	maximum ramus height
MAND L	mandibular length
MAND >	mandibular angle
POSTCRANIAL MEASURMENTS	maximum diameter of clavicle at midshaft
X CLAV L	maximum clavicle length
X MID DIAM CLAV*	maximum diameter of clavicle at midshaft
W MID DIAM CLAV*	minimum diameter of clavicle at midshaft
SCAP HT	height of scapula

ABBREVIATION	MEASUREMENT
SCAP BR	breadth of scapula
GLEN CAV BR*	glenoid cavity breadth
GLEN CAV HT*	glenoid cavity height
X HUM L	maximum length of humerus
EPICOND BR HUM	epicondylar breadth of humerus
VHD HUM	vertical diameter of humeral head
X MID DIAM HUM	maximum diameter of humerus at midshaft
W MID DIAM HUM	minimum diameter of humerus at midshaft
X RAD L	maximum length of radius
X DIAM MID RAD*	maximum diameter of radius at midshaft
W DIAM MID RAD*	minimum diameter of radius at midshaft
X RHD*	maximum diameter of the radial head
X ULNA L	maximum length of the ulna
X MID DIAM ULNA*	maximum midshaft diameter of the ulna
W MID DIAM ULNA*	minimum midshaft diameter of the ulna
PHYS L ULNA°	physiological length of the ulna
W CIRCUM ULNA	minim circumference of the ulna
X BR OLEC ULNA*	maximum breadth of the olecranon process
ANT HT SAC	anterior height of sacrum
ANT BR SAC	anterior breadth of sacrum
TRANS DIAM S1°	transverse diameter of sacral segment 1
AP DIAM S1*	antero-posterior diameter of sacral segment 1
MAX INNOM HT	maximum innominate height
MAX INNOM BR	maximum iliac breadth
X FEM L	maximum femur length
BICOND FEM L	bicondylar length of femur
EPICOND BR FEM	distal epicondylar breadth of femur
X FHD	maximum diameter of femur head
MAX DIAM FEM MID*	maximum diameter of femur at midshaft
MIN DIAM FEM MID*	minimum diameter of femur at midshaft
MID CIRCUM FEM	midshaft circumference of femur
MAX AP DIAM LAT COND FEM*	maximum antero-posterior diameter of lateral femoral condyle
MAX AP DIAM MED COND FEM*	maximum antero-posterior diameter of medial femoral condyle
TIB L	length of tibia
EPICOND BR PROX TIB	proximal epiphyseal breadth of tibia
EPICOND BR DIST TIB°	distal epiphyseal breadth of tibia
X DIAM MID TIB*	maximum midshaft diameter of the tibia
W DIAM MID TIB*	minimum midshaft diameter of tibia
CIRCUM MID TIB*	circumference of tibia at midshaft
X FIB L	maximum length of the fibula
X DIAM MID FIB	maximum midshaft diameter of the fibula
CALC L	maximum length of the calcaneus
CALC BR	middle breadth of the calcaneus

INTEROBSERVER ERROR
Cranial and mandibular measurements.

Repeated measures ANOVA and technical error of measurement (TEM).
Measurements are in alphabetical order.

	REPEATED MEASURES ANOVA				% TEM
	**Sum of Squares**	**Mean Square**	**F-Ratio**	**p-value**	
**ASB**	26.60	2.96	0.11	1.00	0.44
**AUB**	90.93	10.10	0.42	0.93	0.05
**BBH**	9.12	1.01	0.03	1.00	0.25
**BICOND BR**	99.14	11.02	0.28	0.98	0.23
**BIGONIAL BR**	14.78	1.64	0.04	1.00	0.24
**BNL**	25.12	2.79	0.10	1.00	0.50
**BPL**	30.30	3.37	0.07	1.00	0.25
**CHIN HT**	33.66	3.74	0.11	1.00	1.51
**DKB**	119.54	13.28	0.73	0.68	3.45
**EKB**	254.74	28.30	1.01	0.43	0.48
**FOB**	15.37	1.71	0.32	0.97	0.47
**FOL**	3.54	0.39	0.07	1.00	0.39
**FRC**	10.12	1.12	0.04	1.00	0.25
**GOL**	21.57	2.40	0.03	1.00	0.29
**MAB**	153.35	17.04	0.40	0.94	1.12
**MAL**	29.64	3.29	0.17	1.00	0.82
**MAND >**	62.67	6.96	0.11	1.00	2.04
**MAND BODY BR**	36.39	4.04	1.05	0.40	2.61
**MAND BODY HT**	46.21	5.13	0.15	1.00	1.73
**MAND L**	24.16	2.68	0.08	1.00	1.36
**MDH**	339.56	37.73	2.69	0.0044	2.57
**MOW**	44.68	4.96	0.18	1.00	2.20
**NLB**	94.11	10.46	1.15	0.32	0.38
**NLH**	40.17	4.46	0.31	0.97	1.92
**OBB**	36.75	4.08	0.89	0.53	1.73
**OBH**	3.51	0.39	0.09	1.00	0.51
**OCC**	10.98	1.22	0.05	1.00	0.63
**PAC**	351.37	39.04	0.42	0.92	1.33
**UFBR**	3.79	0.42	0.02	1.00	0.15
**UFHT**	99.39	11.04	0.31	0.97	0.38
**WFB**	72.88	8.10	0.27	0.98	0.20
**WRBR**	12.75	1.42	0.09	1.00	0.19
**XCB**	17.49	1.94	0.08	1.00	0.36
**XRHT**	87.52	9.72	0.25	0.99	2.29
**ZMB**	69.32	7.70	0.22	0.99	0.79
**ZYB**	2.11	0.23	0.01	1.00	0.07

INTEROBSERVER ERROR: Postcranial measurements.

Measurement	REPEATED MEASURES ANOVA				% TEM
	Sum of Squares	Mean Square	F-Ratio	p-value	
X CLAV L	21.12	2.35	0.02	1.00	0.24
X MID DIAM CLAV	1.32	0.15	0.07	1.00	0.44
W MID DIAM CLAV	0.63	0.07	0.06	1.00	0.39
SCAP HT	501.64	55.74	0.25	0.99	0.23
SCAP BR	12.27	1.36	0.02	1.00	0.41
GLEN CAV BR	82.57	9.17	1.22	0.28	2.06
GLEN CAV HT	70.75	7.86	0.76	0.65	2.67
X HUM L	20.79	2.31	0.01	1.00	0.13
EPICOND BR HUM	111.69	12.41	0.26	0.98	0.92
VHD HUM	12.20	1.36	0.09	1.00	0.70
X MID DIAM HUM	307.03	34.11	0.89	0.54	0.51
W MID DIAM HUM	0.44	0.05	0.01	1.00	0.22
X RAD L	189.57	21.06	0.07	1.00	0.14
X DIAM MID RAD	4.28	0.48	0.16	1.00	0.32
W DIAM MID RAD	1.38	0.15	0.09	1.00	0.37
X RHD	1.41	0.16	0.03	1.00	0.27
X ULNA L	14.90	1.66	0.01	1.00	0.15
X MID DIAM ULNA	237.27	26.36	0.79	0.63	0.19
W MID DIAM ULNA	1.25	0.14	0.06	1.00	0.77
W CIRCUM ULNA	63.85	7.09	0.64	0.76	2.58
PHYS L ULNA	169.13	18.79	0.08	1.00	0.98
X BR OLEC ULNA	5.27	0.59	0.06	1.00	0.28
ANT HT SAC	33.39	3.71	0.03	1.00	0.37
ANT BR SAC	1346.54	149.62	2.77	0.0035*	12.67
TRANS DIAM S1	598.54	66.50	3.70	0.00015*	14.60
AP DIAM S1	7.44	0.83	0.09	1.00	0.95
INNOM HT	168.42	18.71	0.09	1.00	0.60
INNOM BR	853.60	94.84	0.82	0.60	6.34
X FEM L	980.83	108.98	0.14	1.00	0.12
BICOND FEM L	6101.81	677.98	0.59	0.80	0.13
EPICOND BR FEM	1.12	0.12	0.02	1.00	0.66
X FHD	2.27	0.25	0.09	1.00	0.26
TRANS SUB TROCH	543.69	60.41	1.10	0.36	1.87
AP DIAM SUB TROCH	205.85	22.87	1.90	0.05	1.94
MID CIRCUM FEM	507617.20	56401.91	0.99	0.44	0.35
AP DIAM LAT COND FEM	20.21	2.25	0.06	1.00	0.65
AP DIAM MED COND FEM	11.09	1.23	0.07	1.00	3.03
TIB L	280110.80	31123.42	0.99	0.45	3.01
EPICOND BR PROX TIB	40.28	4.48	0.17	1.00	0.94
EPICOND BR DIST TIB	26.27	2.92	0.17	1.00	10.30*
X DIAM MID TIB	8.47	0.94	0.10	1.00	0.11
W DIAM MID TIB	2.88	0.32	0.02	1.00	0.22
X FIB L	1587.24	176.36	0.26	0.99	0.09
X DIAM MID FIB	38.36	4.26	0.11	1.00	0.33
CALC L	54.45	6.05	0.13	1.00	0.47
CALC BR	26.50	2.94	0.23	0.99	0.68

Repeated measures ANOVA and technical error of measurement (TEM).

*high error due to measurement definition problem; these have been addressed in the revised definitions in DCP 2.0

INTRAOBSERVER ERROR: Cranial and mandibular measurements.

Scaled Error of Measurement (SEM; after Adams and Byrd, 2002). SEI is a measure of repeatability.
Measurement rounds (1-4) are compared for each observer using the median from all rounds.
Measurements are in alphabetical order.   SEI = |MeasurementMedian|Median X 100

	OBSERVER 1				OBSERVER 2				OBSERVER 3				OBSERVER 4			
	SEI 1	SEI 2	SEI 3	SEI 4	SEI 1	SEI 2	SEI 3	SEI 4	SEI 1	SEI 2	SEI 3	SEI 4	SEI 1	SEI 2	SEI 3	SEI 4
**ASB**	0.60	0.48	0.21	0.29	0.75	0.61	0.62	0.52	0.74	0.41	0.46	0.46	0.57	0.91	0.36	0.51
**AUB**	0.06	0.08	0.05	0.06	0.25	0.18	0.15	0.15	0.08	0.05	0.11	0.07	0.23	0.05	0.07	0.06
**BBH**	0.17	0.12	0.17	0.11	0.30	0.35	0.28	0.35	0.29	0.22	0.25	0.17	0.32	0.13	0.13	0.19
**BICOND BR**	0.20	0.05	0.06	0.15	0.33	0.33	0.26	0.31	0.32	0.22	0.30	0.24	0.46	0.18	0.23	0.20
**BIGONIAL BR**	0.39	0.37	0.30	0.28	0.42	0.21	0.28	0.28	0.23	0.21	0.19	0.22	0.24	0.15	0.11	0.21
**BNL**	0.41	0.18	0.16	0.22	0.30	0.36	0.45	0.40	0.42	0.40	0.40	0.32	0.40	0.25	0.24	0.25
**BPL**	0.28	0.22	0.19	0.24	0.39	0.20	0.33	0.22	0.35	0.20	0.15	0.15	0.32	0.23	0.26	0.20
**CHIN HT**	2.02	0.96	0.82	0.78	1.15	0.77	0.49	0.94	1.31	1.39	0.84	0.86	1.13	0.90	1.07	1.19
**DKB**	0.86	0.92	0.95	0.64	5.98	2.52	1.91	1.51	3.67	1.57	2.98	2.08	5.91	2.48	2.36	1.73
**EKB**	0.37	0.27	0.23	0.28	0.38	0.22	0.26	0.46	0.46	0.32	0.43	0.34	1.80	0.31	0.25	0.53
**FOB**	0.94	0.63	0.38	0.44	1.15	0.66	0.67	0.85	1.91	0.87	0.29	0.23	0.36	0.27	0.23	0.25
**FOL**	0.45	0.39	0.53	0.33	1.00	0.44	0.66	0.74	0.66	0.53	0.56	0.32	0.48	0.18	0.18	0.19
**FRC**	0.30	0.20	0.26	0.18	0.38	0.29	0.18	0.19	0.25	0.19	0.19	0.25	0.43	0.50	0.33	0.43
**GOL**	0.25	0.22	0.19	0.14	0.29	0.20	0.21	0.30	0.26	0.25	0.18	0.28	0.24	0.14	0.17	0.17
**MAB**	0.61	0.43	0.46	0.54	0.98	0.66	0.41	0.64	0.84	0.85	0.62	1.06	1.28	0.47	0.64	0.76
**MAL**	0.69	0.69	0.52	0.34	0.83	0.70	0.70	0.67	1.21	0.89	0.76	1.15	0.81	0.56	0.50	0.49
**MAND >**	0.49	0.39	0.43	0.59	0.77	0.77	0.82	0.80	2.95	2.26	2.14	4.50	0.77	0.81	0.56	0.79
**MAND BODY BR**	7.12	2.73	1.33	1.82	7.62	2.51	2.28	2.44	1.67	1.84	3.31	3.72	2.33	0.94	0.84	1.34
**MAND BODY HT**	1.50	1.17	0.88	0.93	5.09	1.07	0.86	2.18	1.43	1.11	1.31	1.10	1.26	1.31	1.11	1.42
**MAND L**	0.51	0.31	0.51	0.50	0.90	0.70	1.15	0.78	1.09	1.20	0.97	1.29	0.71	0.64	0.81	0.60
**MDH**	2.55	1.43	1.74	1.56	10.82	2.33	2.17	1.89	2.11	1.56	1.51	1.74	2.09	1.91	2.13	1.63
**MOW**	1.29	1.05	1.05	1.02	1.33	1.69	1.78	1.23	0.88	0.65	0.88	1.15	1.97	1.58	1.52	1.73

NLB	0.52	0.52	0.50	0.59	0.79	0.96	0.81	0.95	1.40	0.85	2.77	0.53	0.68	0.24	0.26	0.32
NLH	0.79	0.88	0.76	0.53	0.81	0.23	0.27	0.66	0.84	0.87	0.87	0.62	1.24	0.74	0.66	0.71
OBB	0.94	0.45	0.55	0.46	0.85	0.47	0.80	0.77	1.45	1.06	0.88	2.85	2.23	0.93	0.67	1.05
OBH	0.45	0.35	0.32	0.31	0.56	0.61	0.91	0.90	0.48	0.51	0.55	0.43	0.52	0.50	0.28	0.23
OCC	0.57	0.30	0.35	0.32	0.66	0.52	0.44	0.66	0.57	0.81	0.50	0.50	0.73	0.45	0.42	0.38
PAC	0.68	0.46	0.35	0.45	1.05	0.72	0.57	0.72	0.52	0.42	0.41	0.35	0.99	0.64	0.55	0.58
UFBR	0.18	0.12	0.06	0.08	0.23	0.19	0.25	0.23	0.12	0.13	0.11	0.14	0.18	0.11	0.17	0.13
UFHT	0.37	0.38	0.24	0.31	1.13	0.47	0.39	0.48	0.53	0.29	0.39	0.36	0.54	0.38	0.25	0.40
WFB	0.21	0.13	0.14	0.15	0.30	0.16	0.25	0.29	0.18	0.29	0.26	0.22	0.24	0.15	0.17	0.19
WRBR	0.73	0.30	0.30	0.39	0.41	0.35	0.66	0.65	0.23	0.22	0.29	0.23	0.56	0.26	0.24	0.22
XCB	0.20	0.19	0.19	0.10	0.30	0.31	0.28	0.31	0.44	0.31	0.31	0.41	0.32	0.16	0.17	0.21
XRHT	0.37	0.32	0.50	0.50	1.44	0.90	1.22	1.41	1.45	1.72	1.79	1.35	1.28	1.16	1.30	1.45
ZMB	0.39	0.30	0.35	0.40	0.44	0.37	0.55	0.64	0.47	0.44	0.38	0.47	0.49	0.45	0.60	0.42
ZYB	0.09	0.06	0.05	0.04	0.22	0.23	0.27	0.35	0.09	0.07	0.08	0.07	0.16	0.07	0.05	0.04

INTRAOBSERVER ERROR: Postcranial measurements.

Scaled Error of Measurement-median (SEM; after Adams and Byrd, 2002). SEI is a measure of repeatability.

Measurement rounds (1-4) are compared for each observer using the median from all rounds.

SEI = |MeasurementMedian|Median X 100

	OBSERVER 1				OBSERVER 2				OBSERVER 3				OBSERVER 4			
	SEI 1	SEI 2	SEI 3	SEI 4	SEI 1	SEI 2	SEI 3	SEI 4	SEI 1	SEI 2	SEI 3	SEI 4	SEI 1	SEI 2	SEI 3	SEI 4
X CLAV L	0.13	0.04	0.08	0.12	0.14	0.21	0.29	0.23	0.13	0.34	0.24	0.38	0.14	0.06	0.11	0.07
X MID DIAM CLAV	0.53	0.27	0.27	0.24	1.62	0.58	1.62	1.48	1.10	0.64	0.70	0.59	0.61	0.54	0.50	0.57
W MID DIAM CLAV	0.68	0.43	0.29	0.40	1.33	0.51	0.96	1.46	0.59	1.62	0.68	0.68	0.76	0.75	0.53	0.46
SCAP HT	0.13	0.06	0.05	0.09	0.30	0.27	0.22	0.19	0.25	0.36	0.23	0.59	0.27	0.07	0.09	0.07
SCAP BR	0.28	0.19	0.19	0.20	0.29	0.29	0.23	0.20	0.30	0.39	0.33	0.48	0.28	0.17	0.20	0.19
GLEN CAV BR	1.61	0.71	0.98	1.01	1.07	0.60	0.53	0.81	1.61	1.01	0.94	1.11	5.51	0.92	0.47	0.39
GLEN CAV HT	1.64	1.16	0.95	1.63	1.84	1.40	0.75	1.85	1.65	1.06	0.90	1.39	1.21	0.91	0.69	0.93
X HUM L	0.04	0.03	0.02	0.02	0.08	0.09	0.07	0.07	0.08	0.14	0.06	0.18	0.14	0.03	0.02	0.02
EPICOND BR HUM	0.25	0.27	0.24	0.23	1.13	0.84	1.00	0.81	0.69	1.02	0.93	1.10	0.41	0.29	0.33	0.39
VHD HUM	0.75	0.48	0.33	0.49	0.79	0.49	0.34	2.54	0.70	0.40	0.39	0.57	1.07	0.80	0.81	0.74
X MID DIAM HUM	0.52	0.44	0.39	0.40	1.57	0.96	0.72	1.05	0.99	0.79	0.85	1.01	0.81	0.61	0.45	0.49
W MID DIAM HUM	0.31	0.23	0.17	0.16	1.40	0.53	0.50	0.75	0.54	0.59	0.85	0.51	0.42	0.21	0.21	0.13
X RAD L	0.06	0.06	0.04	0.07	0.13	0.12	0.06	0.10	0.12	0.12	0.10	0.25	0.12	0.06	0.06	0.04
X DIAM MID RAD	0.43	0.38	0.26	0.30	1.30	1.31	0.94	1.48	0.94	0.61	0.45	0.70	0.73	0.42	0.42	0.68
W DIAM MID RAD	0.63	0.26	0.22	0.19	0.24	0.30	0.64	0.95	0.61	1.35	0.59	0.51	1.02	0.36	0.54	0.43
X RHD	0.26	0.22	0.24	0.26	0.50	0.63	0.50	0.81	0.45	0.33	0.37	0.37	0.62	0.47	0.76	0.37
X ULNA L	0.02	0.02	0.05	0.05	0.08	0.11	0.09	0.07	0.12	0.11	0.11	0.17	0.17	0.05	0.05	0.11
X MID DIAM ULNA	0.47	0.30	0.30	0.24	1.26	0.84	1.42	1.28	1.11	0.77	0.46	0.80	0.62	0.44	0.39	0.41
W MID DIAM ULNA	0.68	0.24	0.30	0.29	1.69	1.51	1.27	1.75	1.27	0.66	0.91	0.67	1.52	1.12	1.60	0.64
PHYS L ULNA	0.17	0.06	0.08	0.05	0.37	0.19	0.19	0.56	0.37	0.42	0.31	0.28	0.19	0.16	0.11	0.18
W CIRCUM ULNA	1.32	1.23	0.97	1.39	3.07	2.61	2.53	2.38	2.33	1.97	2.41	1.29	1.01	0.78	0.83	1.47
X BR OLEC ULNA	0.45	0.37	0.39	0.37	0.93	0.69	0.90	0.97	0.53	1.06	0.45	0.69	0.63	0.29	0.34	0.36
ANT HT SAC	0.45	0.31	0.69	0.32	0.89	0.56	0.58	0.62	0.46	0.93	0.46	0.67	0.68	0.46	0.50	0.43

**ANT BR SAC**	1.89	1.18	0.76	0.88	1.25	1.17	1.13	1.66	1.64	1.18	0.96	1.03	0.59	0.59	1.07	1.58
**TRANS DIAM S1**	1.43	0.80	0.78	0.70	1.44	0.86	0.80	2.83	1.45	1.22	0.94	1.08	6.27	2.41	2.52	2.72
**AP DIAM S1**	0.60	0.57	0.40	0.44	1.39	0.93	0.86	0.62	1.38	0.98	0.91	0.98	0.70	0.61	0.47	0.80
**INNOM HT**	0.14	0.13	0.14	0.11	0.29	0.25	0.34	0.21	0.43	0.45	0.37	0.54	0.74	0.35	0.35	0.33
**INNOM BR**	0.30	0.43	0.21	0.17	0.66	0.73	0.75	0.54	0.47	0.69	0.58	0.61	1.39	0.49	0.60	0.53
**X FEM L**	0.02	0.03	0.03	0.05	0.08	0.08	0.09	0.10	0.08	0.06	0.05	0.11	0.16	0.03	0.04	0.04
**BICOND FEM L**	0.04	0.04	0.03	0.04	0.03	0.08	0.05	0.06	0.07	0.09	0.09	0.16	0.15	0.04	0.03	0.05
**EPICOND BR FEM**	0.19	0.07	0.07	0.15	0.58	0.69	0.67	0.68	0.45	0.65	0.57	0.91	0.45	0.05	0.17	0.20
**X FHD**	0.37	0.26	0.27	0.46	0.54	0.30	0.22	0.40	0.39	0.29	0.34	0.43	0.37	0.23	0.29	0.25
**AP DIAM SUB TROCH**	2.26	1.02	1.18	0.89	1.23	0.95	5.90	1.08	2.03	2.04	1.57	3.26	4.44	1.29	1.47	1.13
**TRANS SUB TROCH**	2.20	1.56	1.46	1.46	1.11	1.21	0.90	1.11	2.93	1.53	1.24	1.97	4.71	1.26	1.98	1.16
**MID CIRCUM FEM**	0.51	0.47	0.45	0.66	1.12	0.66	0.47	0.50	0.47	0.50	0.69	0.54	0.32	0.28	0.20	0.50
**AP DIAM LAT COND FEM**	0.59	0.30	0.35	0.41	0.73	0.40	0.43	0.65	0.46	0.47	0.54	0.62	0.44	0.21	0.24	0.31
**AP DIAM MED COND FEM**	0.68	0.66	0.65	0.46	0.89	0.64	0.55	0.55	0.75	0.88	0.78	0.88	0.74	0.50	0.37	0.56
**TIB L**	0.11	0.08	0.07	0.08	0.09	0.11	0.12	0.09	0.21	0.10	0.09	0.16	0.39	0.28	0.27	0.23
**EPICOND BR PROX TIB**	0.38	0.23	0.14	0.19	0.82	1.00	0.90	0.81	0.86	0.84	0.64	1.24	0.78	0.26	0.24	0.40
**EPICOND BR DIST TIB**	0.69	0.43	0.30	0.59	1.47	1.29	1.05	1.09	1.32	1.66	1.17	1.99	0.72	0.49	0.50	0.54
**X DIAM MID TIB**	0.26	0.18	0.19	0.14	0.56	0.63	0.38	0.36	0.54	0.31	0.46	0.39	0.51	0.35	0.30	0.40
**W DIAM MID TIB**	0.33	0.25	0.27	0.19	0.83	0.76	0.94	0.72	1.35	1.10	1.11	0.99	1.07	0.60	0.52	0.74
**X FIB L**	0.04	0.03	0.04	0.03	0.09	0.15	0.09	0.05	0.11	0.06	0.07	0.11	0.11	0.05	0.04	0.03
**X DIAM MID FIB**	0.61	0.45	0.37	0.27	1.14	0.71	1.01	0.56	1.28	1.13	0.95	1.15	0.91	0.49	0.42	0.49
**CALC L**	0.36	0.46	0.28	0.40	0.86	0.49	0.53	0.55	0.85	0.80	0.62	0.81	0.88	0.34	0.26	0.46
**CALC BR**	0.80	0.49	0.31	0.48	0.64	0.71	0.80	0.56	0.70	0.55	0.56	0.43	1.41	0.72	0.66	0.49

SEI Cranial

	OBSERVER 1				OBSERVER 2				OBSERVER 3				OBSERVER 4			
	SEI 1	SEI 2	SEI 3	SEI 4	SEI 1	SEI 2	SEI 3	SEI 4	SEI 1	SEI 2	SEI 3	SEI 4	SEI 1	SEI 2	SEI 3	SEI 4
GOL	0.25	0.22	0.19	0.14	0.29	0.20	0.21	0.30	0.26	0.25	0.18	0.28	0.24	0.14	0.17	0.17
XCB	0.20	0.19	0.19	0.10	0.30	0.31	0.28	0.31	0.44	0.31	0.31	0.41	0.32	0.16	0.17	0.21
ZYB	0.09	0.06	0.05	0.04	0.22	0.23	0.27	0.35	0.09	0.07	0.08	0.07	0.16	0.07	0.05	0.04
BBH	0.17	0.12	0.17	0.11	0.30	0.35	0.28	0.35	0.29	0.22	0.25	0.17	0.32	0.13	0.13	0.19
BNL	0.41	0.18	0.16	0.22	0.30	0.36	0.45	0.40	0.42	0.40	0.40	0.32	0.40	0.25	0.24	0.25
BPL	0.28	0.22	0.19	0.24	0.39	0.20	0.33	0.22	0.35	0.20	0.15	0.15	0.32	0.23	0.26	0.20
MAB	0.61	0.43	0.46	0.54	0.98	0.66	0.41	0.64	0.84	0.85	0.62	1.06	1.28	0.47	0.64	0.76
MAL	0.69	0.69	0.52	0.34	0.83	0.70	0.70	0.67	1.21	0.89	0.76	1.15	0.81	0.56	0.50	0.49
AUB	0.06	0.08	0.05	0.06	0.25	0.18	0.15	0.15	0.08	0.05	0.11	0.07	0.23	0.05	0.07	0.06
UFHT	0.37	0.38	0.24	0.31	1.13	0.47	0.39	0.48	0.53	0.29	0.39	0.36	0.54	0.38	0.25	0.40
WFB	0.21	0.13	0.14	0.15	0.30	0.16	0.25	0.29	0.18	0.29	0.26	0.22	0.24	0.15	0.17	0.19
UFBR	0.18	0.12	0.06	0.08	0.23	0.19	0.25	0.23	0.12	0.13	0.11	0.14	0.18	0.11	0.17	0.13
NLH	0.79	0.88	0.76	0.53	0.81	0.23	0.27	0.66	0.84	0.87	0.87	0.62	1.24	0.74	0.66	0.71
NLB	0.52	0.52	0.50	0.59	0.79	0.96	0.81	0.95	1.40	0.85	2.77	0.53	0.68	0.24	0.26	0.32
OBB	0.94	0.45	0.55	0.46	0.85	0.47	0.80	0.77	1.45	1.06	0.88	2.85	2.23	0.93	0.67	1.05
OBH	0.45	0.35	0.32	0.31	0.56	0.61	0.91	0.90	0.48	0.51	0.55	0.43	0.52	0.50	0.28	0.23
EKB	0.37	0.27	0.23	0.28	0.38	0.22	0.26	0.46	0.46	0.32	0.43	0.34	1.80	0.31	0.25	0.53
DKB	0.86	0.92	0.95	0.64	5.98	2.52	1.91	1.51	3.67	1.57	2.98	2.08	5.91	2.48	2.36	1.73
FRC	0.30	0.20	0.26	0.18	0.38	0.29	0.18	0.19	0.25	0.19	0.19	0.25	0.43	0.50	0.33	0.43
PAC	0.68	0.46	0.35	0.45	1.05	0.72	0.57	0.72	0.52	0.42	0.41	0.35	0.99	0.64	0.55	0.58
OCC	0.57	0.30	0.35	0.32	0.66	0.52	0.44	0.66	0.57	0.81	0.50	0.50	0.73	0.45	0.42	0.38
FOL	0.45	0.39	0.53	0.33	1.00	0.44	0.66	0.74	0.66	0.53	0.56	0.32	0.48	0.18	0.18	0.19
FOB	0.94	0.63	0.38	0.44	1.15	0.66	0.67	0.85	1.91	0.87	0.29	0.23	0.36	0.27	0.23	0.25
MOW	1.29	1.05	1.05	1.02	1.33	1.69	1.78	1.23	0.88	0.65	0.88	1.15	1.97	1.58	1.52	1.73
ASB	0.60	0.48	0.21	0.29	0.75	0.61	0.62	0.52	0.74	0.41	0.46	0.46	0.57	0.91	0.36	0.51
ZMB	0.39	0.30	0.35	0.40	0.44	0.37	0.55	0.64	0.47	0.44	0.38	0.47	0.49	0.45	0.60	0.42

**MDH (SIGHT)**	2.57	1.09	1.57	1.78	13.04	2.38	2.01	2.62	1.70	1.30	1.63	1.82	2.46	1.89	1.91	2.45
**MDH (TIP)**	2.55	1.43	1.74	1.56	10.82	2.33	2.17	1.89	2.11	1.56	1.51	1.74	2.09	1.91	2.13	1.63
**CHIN HT**	2.02	0.96	0.82	0.78	1.15	0.77	0.49	0.94	1.31	1.39	0.84	0.86	1.13	0.90	1.07	1.19
**MAND BODY HT**	1.50	1.17	0.88	0.93	5.09	1.07	0.86	2.18	1.43	1.11	1.31	1.10	1.26	1.31	1.11	1.42
**MAND BODY BR**	7.12	2.73	1.33	1.82	7.62	2.51	2.28	2.44	1.67	1.84	3.31	3.72	2.33	0.94	0.84	1.34
**BIGONIAL BR**	0.39	0.37	0.30	0.28	0.42	0.21	0.28	0.28	0.23	0.21	0.19	0.22	0.24	0.15	0.11	0.21
**BICOND BR**	0.20	0.05	0.06	0.15	0.33	0.33	0.26	0.31	0.32	0.22	0.30	0.24	0.46	0.18	0.23	0.20
**WRBR**	0.73	0.30	0.30	0.39	0.41	0.35	0.66	0.65	0.23	0.22	0.29	0.23	0.56	0.26	0.24	0.22
**XRBR**	1.26	0.97	0.80	1.26	1.60	0.59	0.52	0.60	1.10	1.03	0.74	0.97	0.79	0.40	0.52	0.31
**XRHT**	0.37	0.32	0.50	0.50	1.44	0.90	1.22	1.41	1.45	1.72	1.79	1.35	1.28	1.16	1.30	1.45
**MAND L**	0.51	0.31	0.51	0.50	0.90	0.70	1.15	0.78	1.09	1.20	0.97	1.29	0.71	0.64	0.81	0.60
**MAND** >	0.49	0.39	0.43	0.59	0.77	0.77	0.82	0.80	2.95	2.26	2.14	4.50	0.77	0.81	0.56	0.79

TEM Cranial

Measurement	REPEATED MEASURES ANOVA				% TEM
	Sum of Squares	Mean Square	F-Ratio	p-value	
AUB	90.93	10.10	0.42	0.93	0.05
ZYB	2.11	0.23	0.01	1.00	0.07
UFBR	3.79	0.42	0.02	1.00	0.15
WRBR	12.75	1.42	0.09	1.00	0.19
WFB	72.88	8.10	0.27	0.98	0.20
BICOND BR	99.14	11.02	0.28	0.98	0.23
BIGONIAL BR	14.78	1.64	0.04	1.00	0.24
BBH	9.12	1.01	0.03	1.00	0.25
FRC	10.12	1.12	0.04	1.00	0.25
BPL	30.30	3.37	0.07	1.00	0.25
GOL	21.57	2.40	0.03	1.00	0.29
XCB	17.49	1.94	0.08	1.00	0.36
NLB	94.11	10.46	1.15	0.32	0.38
UFHT	99.39	11.04	0.31	0.97	0.38
FOL	3.54	0.39	0.07	1.00	0.39
ASB	26.60	2.96	0.11	1.00	0.44
FOB	15.37	1.71	0.32	0.97	0.47
EKB	254.74	28.30	1.01	0.43	0.48
BNL	25.12	2.79	0.10	1.00	0.50
OBH	3.51	0.39	0.09	1.00	0.51
OCC	10.98	1.22	0.05	1.00	0.63
ZMB	69.32	7.70	0.22	0.99	0.79
MAL	29.64	3.29	0.17	1.00	0.82
MAB	153.35	17.04	0.40	0.94	1.12
PAC	351.37	39.04	0.42	0.92	1.33
MAND L	24.16	2.68	0.08	1.00	1.36
CHIN HT	33.66	3.74	0.11	1.00	1.51
MAND BODY HT	46.21	5.13	0.15	1.00	1.73
OBB	36.75	4.08	0.89	0.53	1.73
NLH	40.17	4.46	0.31	0.97	1.92
MAND >	62.67	6.96	0.11	1.00	2.04
MOW	44.68	4.96	0.18	1.00	2.20
XRHT	87.52	9.72	0.25	0.99	2.29
MDH (TIP)	339.56	37.73	2.69	0.0044*	2.57
MAND BODY BR	36.39	4.04	1.05	0.40	2.61
XRBR	431.64	47.96	1.52	0.14	2.85
DKB	119.54	13.28	0.73	0.68	3.45
MDH (SIGHT)	497.68	55.30	3.92	0.000068*	7.06
